# Association Between Atopic Eczema and Cancer in England and Denmark

**DOI:** 10.1001/jamadermatol.2020.1948

**Published:** 2020-06-24

**Authors:** Kathryn E. Mansfield, Sigrún A. J. Schmidt, Bianka Darvalics, Amy Mulick, Katrina Abuabara, Angel Y. S. Wong, Henrik Toft Sørensen, Liam Smeeth, Krishnan Bhaskaran, Isabel dos Santos Silva, Richard J. Silverwood, Sinéad M. Langan

**Affiliations:** 1 Department of Non-communicable Disease Epidemiology, London School of Hygiene and Tropical Medicine, London, United Kingdom; 2 Department of Clinical Epidemiology, Aarhus University Hospital, Aarhus N, Denmark; 3 Department of Dermatology, Aarhus University Hospital, Aarhus N, Denmark; 4 Department of Dermatology, University of California, San Francisco; 5 Department of Medical Statistics, London School of Hygiene and Tropical Medicine, London, United Kingdom; 6 Centre for Longitudinal Studies, Department of Social Science, University College London, London, United Kingdom; 7 Health Data Research UK, London, United Kingdom

## Abstract

**Importance:**

Associations between atopic eczema and cancer are unclear, with competing theories that increased immune surveillance decreases cancer risk and that immune stimulation increases cancer risk. Establishing baseline cancer risk in people with atopic eczema is important before exploring the association between new biologic drugs for atopic eczema and cancer risk.

**Objective:**

To investigate whether atopic eczema is associated with cancer.

**Design, Setting, and Participants:**

Matched cohort studies were conducted from January 2, 1998, to March 31, 2016, in England and from January 1, 1982, to June 30, 2016, in Denmark. We conducted our analyses between July 2018 and July 2019. The setting was English primary care and nationwide Danish data. Participants with atopic eczema (adults only in England and any age in Denmark) were matched on age, sex, and calendar period (as well as primary care practice in England only) to those without atopic eczema.

**Exposure:**

Atopic eczema.

**Main Outcomes and Measures:**

Overall cancer risk and risk of specific cancers were compared in people with and without atopic eczema.

**Results:**

In England, matched cohorts included 471 970 individuals with atopic eczema (median [IQR] age, 41.1 [24.9-60.7] years; 276 510 [58.6%] female) and 2 239 775 individuals without atopic eczema (median [IQR] age, 39.8 [25.9-58.4] years; 1 301 074 [58.1%] female). In Denmark, matched cohorts included 44 945 individuals with atopic eczema (median [IQR] age, 13.7 [1.7-21.1] years; 22 826 [50.8%] female) and 445 673 individuals without atopic eczema (median [IQR] age, 13.5 [1.7-20.8] years; 226 323 [50.8%] female). Little evidence was found of associations between atopic eczema and overall cancer (adjusted hazard ratio [HR], 1.04; 99% CI, 1.02-1.06 in England and 1.05; 99% CI, 0.95-1.16 in Denmark) or for most specific cancers. However, noncutaneous lymphoma risk was increased in people with atopic eczema in England (adjusted HR, 1.19; 99% CI, 1.07-1.34 for non-Hodgkin lymphoma [NHL] and 1.48; 99% CI, 1.07-2.04 for Hodgkin lymphoma). Lymphoma risk was increased in people with greater eczema severity vs those without atopic eczema (NHL adjusted HR, 1.06; 99% CI, 0.90-1.25 for mild eczema; 1.24; 99% CI, 1.04-1.48 for moderate eczema; and 2.08; 99% CI, 1.42-3.04 for severe eczema). Danish point estimates also showed increased lymphoma risk in people with moderate to severe eczema compared with those without atopic eczema (minimally adjusted HR, 1.31; 99% CI, 0.76-2.26 for NHL and 1.35; 99% CI, 0.65-2.82 for Hodgkin lymphoma), but the 99% CIs were wide.

**Conclusions and Relevance:**

The findings from 2 large population-based studies performed in different settings do not support associations between atopic eczema and most cancers. However, an association was observed between atopic eczema and lymphoma, particularly NHL, that increased with eczema severity. This finding warrants further study as new immunomodulatory systemic therapeutics are brought to market that may alter cancer risk.

## Key Points

**Question:**

Is atopic eczema associated with increased cancer risk?

**Findings:**

In 2 large cohort studies conducted in England (471 970 and 2 239 775 individuals with and without atopic eczema, respectively) and Denmark (44 945 and 445 673 individuals with and without atopic eczema, respectively), no evidence was found of an increased risk of most cancers among people with atopic eczema compared with those without eczema. However, atopic eczema was associated with an increased risk of lymphoma, particularly non-Hodgkin lymphoma, with risk increasing with greater eczema severity.

**Meaning:**

The findings in this study did not support an association between atopic eczema and most cancers; however, there was evidence of higher lymphoma risk with increasing eczema severity.

## Teaser

Two large matched cohort studies using health care data from England and Denmark investigate cancer risk in people with and without atopic eczema, including variation in risk associated with eczema severity and activity.

## Introduction

Atopic eczema is the most common inflammatory skin disease, affecting 2% to 10% of adults^1,2,3^ and up to 20% of children.^4^ Eczema may be associated with cancer for many reasons, including immune dysregulation related to eczema, unhealthy lifestyle choices (eg, smoking and alcohol consumption as coping mechanisms for persistent itch), stress, low self-esteem, and sleep deprivation.^5,6^ Although most eczema is managed with topical treatment, systemic treatments (eg, glucocorticoids, azathioprine, and cyclosporine) for severe disease could also affect cancer risk.^7^

Cancer is 1 of the leading causes of death globally.^8^ Skin disease is the most common reason for a new primary care practice consultation in England,^9,10^ and eczema accounts for a substantial proportion of that burden.^9^ Hence, any association between atopic eczema and cancer is potentially relevant to public health. Furthermore, as new therapeutics (that may alter cancer risk) are introduced, it is important to establish baseline cancer risk in people with atopic eczema.^11^

A recent systematic review and meta-analysis^12^ highlighted that existing evidence of cancer risk in atopic eczema is conflicting, with studies limited by lack of consideration of eczema severity and treatment and by the inability to adjust for key confounders. Studies have reported reductions in specific brain cancers and increased lymphoma risk in people with eczema.^5,6,13,14,15,16,17,18,19^ Two competing theories may explain the complex association between atopic eczema and cancer, namely, that increased immune surveillance decreases cancer risk and that immune stimulation increases cancer risk. Immunosuppressive therapy (including topical calcineurin inhibitors) and an impaired skin barrier may also increase risk, particularly of skin cancer, but recent findings are inconclusive.^20^ Experimental mouse models support reduced skin cancer in individuals with atopic eczema,^21^ but population-based studies are conflicting.^6,22,23,24,25^

We used health care data from matched cohort studies conducted in England and Denmark to address uncertainties identified by the recent systematic review and meta-analysis.^12^ We explored cancer risk in persons with atopic eczema compared with those without atopic eczema, including variation in risk with eczema severity and activity.

## Methods

This investigation was composed of 2 matched cohort studies conducted from January 2, 1998, to March 31, 2016, in England and from January 1, 1982, to June 30, 2016, in Denmark. We conducted our analyses between July 2018 and July 2019. Participants with atopic eczema (adults only in England and any age in Denmark) were matched on age, sex, and calendar period (as well as primary care practice in England only) to people without atopic eczema. We compared overall cancer risk and risk of specific cancers in those with and without atopic eczema. The English study was approved by the London School of Hygiene and Tropical Medicine Research Ethics Committee and by the United Kingdom Clinical Practice Research Datalink (CPRD) Independent Scientific Advisory Committee (protocol 17_108). The English trial protocol is available at Supplement 1. The Danish study was approved by the Danish Data Protection Agency (record 2015-57-0002; AU-2016-051-000001; 632). The Danish trial protocol is available at Supplement 2. These are deidentified data and do not require explicit consent; individual consent for use of electronic health record data for research is implied. In England, individuals are offered the right to opt out of the use of their anonymized data. Danish legislation does not require approval by an ethical review board or informed consent from participants in registry-based studies.

**Figure 1.**
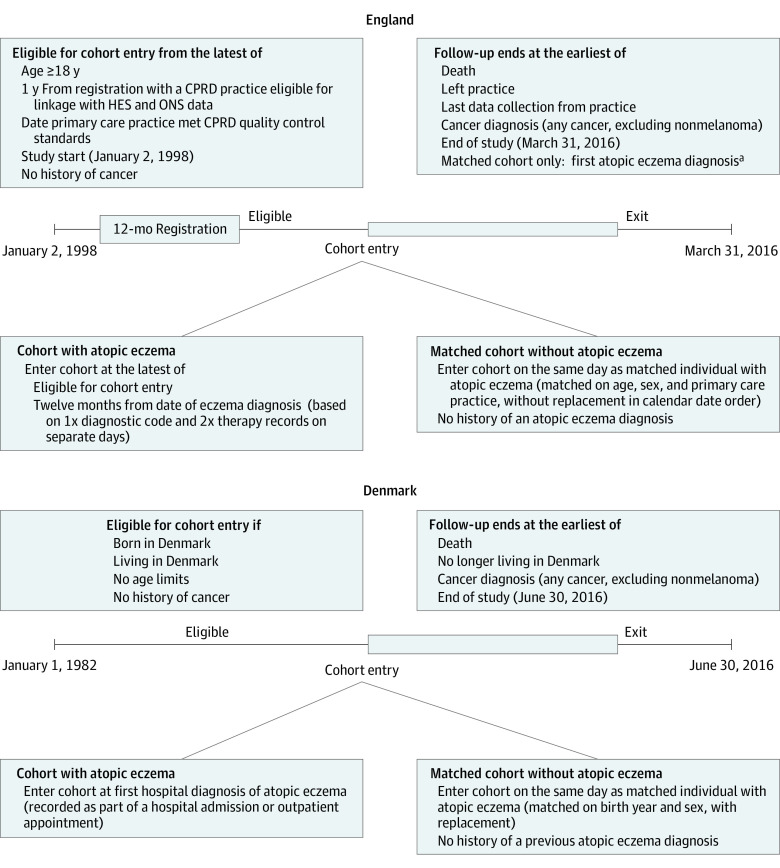
Two Matched Cohorts of People With vs Without Atopic Eczema in England and Denmark CPRD indicates Clinical Practice Research Datalink; HES, Hospital Episode Statistics; and ONS, Office for National Statistics. ^a^In England, individuals from the matched cohort without atopic eczema were censored if they had a diagnosis of atopic eczema and were then included in the atopic eczema cohort if they had 2 records for atopic eczema therapies.

Two matched cohorts of people with and without atopic eczema in England and Denmark were identified (Table 1 and Figure 1). We did not include children in the English study (unlike the Danish study, in which we did not restrict age) because of limited power to study childhood cancers. Childhood cancer is rare, and the median follow-up in the CPRD is only 5 years.^26^

**Table 1.** Summary of English and Danish Study Designs

Variable	England	Denmark
Sampling frame	Individuals ≥18 y registered with primary care practices contributing to the CPRD GOLD and eligible for linkage with hospital record data between January 2, 1998, and March 31, 2016. The CPRD is broadly representative of the whole population. We did not include children in the English study (unlike the Danish study, in which we did not restrict on age) because of limited power to study childhood cancers (childhood cancer is rare, and the median follow-up in the CPRD is only 5 y^26^).	All individuals (no age restriction) born and living in Denmark between January 1, 1982, and June 30, 2016
Atopic eczema definition	Atopic eczema identified based on a record of 1 atopic eczema diagnostic code recorded in primary or secondary care and ≥2 primary care records (diagnosis code or prescription) for atopic eczema therapy	Atopic eczema identified based on an atopic eczema diagnosis, recorded as part of a hospital admission, emergency department contact, or an outpatient appointment. Therefore, the Danish cohort included people with more severe disease than the English cohort because it was based on hospital diagnoses only.
Comparison population	A cohort of individuals without atopic eczema matched on age (within 15 y), sex, calendar period, and primary care practice. We randomly matched (without replacement) up to 5 individuals without atopic eczema for every individual with atopic eczema in calendar date order (ie, individuals in the matched cohort were assigned first to those with earliest cohort entry to avoid time-related bias). Individuals were censored and no longer included in the comparison population on the date of their first atopic eczema diagnosis (they were subsequently included in the atopic eczema cohort if they also had ≥2 records for atopic eczema therapies).	A cohort of up to 10 individuals matched (with replacement) on sex and exact birth year to each individual with atopic eczema. Individuals included in the matched cohort were Danish residents who were born in Denmark and had no previous atopic eczema diagnosis on the date of the first recorded atopic eczema diagnosis (index date) of their matched atopic eczema–exposed individual. In Denmark, we were able to match with up to 10 individuals (rather than up to 5 in England) because we matched with replacement and did not match on primary care practice.
Cohort entry date (index date)	Prevalent cohort of atopic eczema-exposed individuals entered the cohort (index date) on the latest of 1 y after the date of registration with their primary care practice, the date that the practice met CPRD quality control standards, the start of the study (January 2, 1998), their 18th birthday, or 12 mo from the date of atopic eczema diagnosis (to limit potential reverse causality)	New hospital diagnosis cohort individuals entered the cohort (index date) on the date of their first atopic eczema diagnosis, recorded either as part of a hospital admission or an outpatient appointment
Cohort exit date	Earliest of the following events: death, individual no longer registered with the practice, practice no longer contributing data to the CPRD, end of study (March 31, 2016), diagnosis of atopic eczema (matched cohort only), or first-ever cancer diagnosis (excluding nonmelanoma skin cancer)	Earliest of the following events: death, emigration, diagnosis of atopic eczema (matched cohort only), first-ever cancer diagnosis (excluding nonmelanoma skin cancer), or end of study (June 30, 2016)
Cancer definition	Identified based on morbidity codes recorded in primary care (CPRD) or as part of a hospital admission (HES) and cause of death coding in ONS data	Identified based on diagnosis codes recorded in the Danish Cancer Registry
Confounders considered	Age, sex, calendar period, and IMD (as a proxy for socioeconomic deprivation)	Age and sex. Highest educational level, partner status, and gross personal income were used as proxies for socioeconomic status in people 30 y or older only (because these measures are likely to be incomplete for those aged <30 y).
Mediators considered	Lifestyle factors, including BMI, smoking, and harmful alcohol use	Because data on lifestyle variables were not available in Denmark, we used diagnoses or treatments for diseases associated with lifestyle factors as proxies, including chronic obstructive pulmonary disease, hyperlipidemia, hypertension, alcohol-related conditions, ischemic heart disease, hospital-diagnosed obesity, and type 2 diabetes

Abbreviations: BMI, body mass index (calculated as weight in kilograms divided by height in meters squared); CPRD, Clinical Practice Research Datalink; HES, Hospital Episode Statistics; IMD, Index of Multiple Deprivation; ONS, Office for National Statistics.

### Data Sources

In England, electronic health records of adults attending primary care practices contributing to the CPRD GOLD were used along with linked hospital admissions (Hospital Episode Statistics [HES]) and death registration (Office for National Statistics [ONS]) data. The CPRD includes data from participating primary care practices covering 7% of the UK population.^26,27,28^ The HES covers all admissions for National Health Service–funded patients treated in England.^29^ Most English primary care practices contributing to the CPRD (75%) are linked to the HES.^26^ The ONS mortality data include cause of death for all deaths in the UK.

In Denmark, linked nationwide data from the Danish National Patient Registry (hospital admissions, outpatient clinic appointments, and emergency department contacts),^30,31^ the Danish Cancer Registry (incident cancers),^32^ the Civil Registration System (demographics),^33^ the Danish National Prescription Registry (prescriptions filled at community pharmacies),^34^ and socioeconomic data were used. The socioeconomic data included highest educational level and gross personal income gathered by Statistics Denmark.^35,36^

Morbidity code lists and additional details of all variable definitions are provided in eAppendix 1 and eTables 16, 17, and 18 in Supplement 3. Complete code lists for all variables used in the English study are available online.^37^

### Study Cohorts, Exposure, and Outcomes

#### England

All adults (aged ≥18 years) registered with primary care practices contributing data meeting CPRD quality control standards between 1998 and 2016 were eligible for inclusion (Figure 1 and eAppendix 2 in Supplement 3). Individuals needed at least 1 year of registration before cohort entry. Individuals with atopic eczema were identified based on a previously validated algorithm (eAppendix 1 in Supplement 3).^38^ We randomly selected an individually matched cohort (without replacement) of up to 5 people for each individual with atopic eczema by age, sex, and primary care practice (eAppendix 2 in Supplement 3).

#### Denmark

All individuals (any age) born in Denmark with a hospital-based (outpatient and inpatient records) diagnosis of atopic eczema (mostly moderate to severe eczema) between 1982 and 2016 were identified (Figure 1 and eAppendix 2 in Supplement 3). Also identified was a comparison cohort (with replacement) of up to 10 people without atopic eczema matched to each individual with atopic eczema by sex and exact birth year.^39^

#### Exclusions

In England and Denmark, we excluded individuals with any history of cancer (except nonmelanoma skin cancer [NMSC] or keratinocyte cancer). In analyses focused specifically on skin cancer, we further excluded individuals with a previous NMSC record.

#### Outcomes

**Figure 2.**
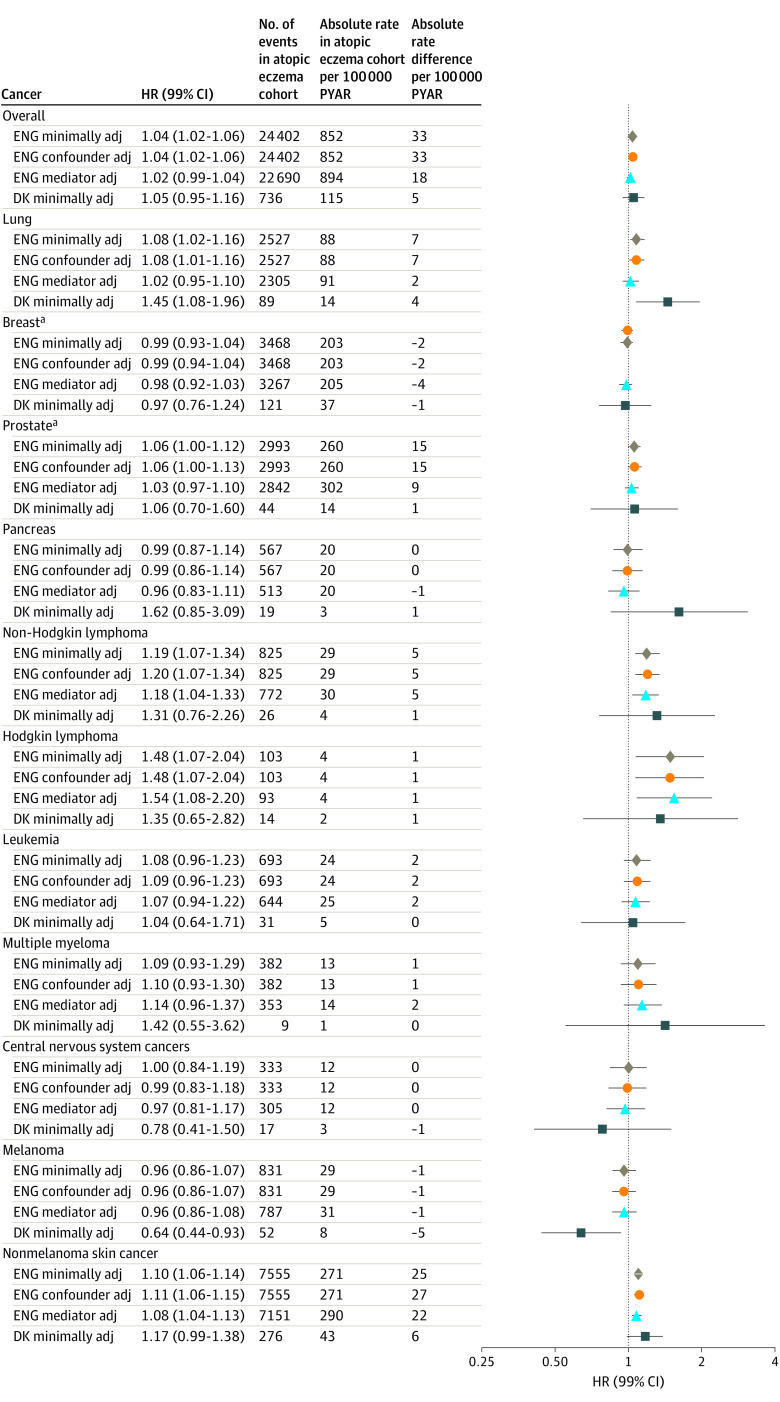
Associations Between Atopic Eczema and Cancer Outcomes in England and Denmark in People With vs Without Atopic Eczema All models implicitly adjusted for sex, primary care practice (England [ENG] only) and date at cohort entry (because of stratification by matched set), and age (because of the underlying timescale). Confounders: Index of Multiple Deprivation (IMD) and calendar period. Potential mediators: body mass index, smoking, and harmful alcohol use. Adj indicates adjusted; DK, Denmark; HR indicates hazard ratio; and PYAR, person-years at risk. ^a^Analysis of breast cancer was limited to women. Analysis of prostate cancer was limited to men.

We investigated the association between atopic eczema and cancer, overall and the site-specific cancers (Figure 2), chosen a priori because they are common or were previously associated with atopic eczema or atopy.^5^ Overall cancer excluded NMSCs because of possible ascertainment bias (frequent skin examinations in atopic eczema) and possible poor capture because NMSCs may be treated topically (eg, photodynamic therapy) without histological confirmation.^40^ We excluded cutaneous lymphomas from lymphoma definitions to reduce ascertainment bias because of frequent skin examinations or misdiagnosis of cutaneous lymphoma as eczema. In site-specific cancer analyses, we censored individuals at diagnosis of any cancer (excluding NMSC) and additionally at first NMSC diagnosis in skin cancer analyses.

#### Covariates

We considered the following variables as potential confounders: age, sex, calendar period, and socioeconomic status. In England, we used the Index of Multiple Deprivation (IMD) as a measure of socioeconomic deprivation of residential neighborhood.^41,42,43^ In Denmark, we used highest educational level, partner status, and gross personal income as measures of socioeconomic status.

Also considered were factors potentially on the pathway between atopic eczema and cancer (ie, potential mediators), specifically body mass index (BMI), smoking, and harmful alcohol use (in England). Data on these variables were not available in Denmark; we relied instead on diagnoses or treatments for diseases associated with lifestyle factors as proxies, including chronic obstructive pulmonary disease, hyperlipidemia, hypertension, alcohol-related conditions, ischemic heart disease, hospital-diagnosed obesity, and type 2 diabetes.

### Statistical Analysis

#### Main Analysis

We considered descriptive characteristics for individuals with and without atopic eczema. We used Cox proportional hazards regression, stratified by matched set, to estimate hazard ratios (HRs) comparing overall cancer risk and risk of specific cancers in people with and without atopic eczema. In England, we used age as the underlying timescale. In Denmark, because those with atopic eczema entered the cohort at first hospital atopic eczema diagnosis, we used time since first atopic eczema diagnosis for individuals with atopic eczema and the corresponding cohort entry date for matched individuals.

The minimally adjusted models implicitly accounted for matching and timescale factors, including age, sex, cohort entry date, and (in England only) primary care practice. In England, we followed with sequential models adjusting for (1) confounders (IMD and calendar period) to estimate the potential association between atopic eczema and cancer and then (2) potential mediators that might be on the pathway between atopic eczema and cancer. Sequential models, adjusting first for confounders and then for potential mediators, allowed us to differentiate between possible direct (eg, inflammatory) and indirect (ie, via mediating variables [eg, lifestyle factors]) associations of atopic eczema with cancer.^44^ In Denmark, we were only able to adjust for potential confounders (ie, socioeconomic status) in sensitivity analyses restricted to individuals 30 years or older at cohort entry (socioeconomic status may be unreliable in younger people, who are less likely to have attained their highest educational level and have partner status and gross personal income recorded).

We repeated our main analyses in a series of sensitivity analyses assessing the robustness of our findings. Details are listed in eTable 1 in Supplement 3.

#### Secondary Analyses

In our secondary analyses, we investigated whether the association between atopic eczema and cancer was (1) more pronounced in individuals with severe or active atopic eczema (unfeasible in Denmark because of small event numbers) and (2) modified by age, sex, or presence of asthma. Details are available in eAppendix 3 in Supplement 3.

We assessed the proportional hazards assumption using Schoenfeld residual plots. In all analyses, as per our approved protocols (listed in the Methods section), we used 99% CIs to reduce the risk of type I error in the context of multiple analyses.^45^ For data management and analyses, Stata, version 15 (StataCorp LLC), and SAS, version 9.4 (SAS Institute Inc), were used.

## Results

In England, 2 629 640 individuals remained in valid matched sets and were included in analyses (eFigure 1 and eTable 2 in Supplement 3). The matched cohorts in England included 471 970 individuals with atopic eczema (median [IQR] age, 41.1 [24.9-60.7] years; 276 510 [58.6%] female) and 2 239 775 individuals without atopic eczema (median [IQR] age, 39.8 [25.9-58.4] years; 1 301 074 [58.1%] female).

In Denmark, 490 618 individuals remained in valid matched sets and were included in the analyses (eFigure 2 in Supplement 3). The Danish matched cohorts included 44 945 individuals with atopic eczema (median [IQR] age, 13.7 [1.7-21.1] years; 22 826 [50.8%] female) and 445 673 individuals without atopic eczema (median [IQR] age, 13.5 [1.7-20.8] years; 226 323 [50.8%] female).

In England, the median follow-up was 4.5 years (IQR, 1.7-9.0 years). The cohorts with and without atopic eczema had broadly similar age, sex, and IMD (Table 2). Prevalence of smoking or overweight/obesity was higher in individuals with atopic eczema than in those without (46.7% vs 43.5% for smoking and 44.6% vs 40.7% for overweight/obesity). However, smoking and BMI data were less likely to be missing in people with atopic eczema compared with those without, and those with missing BMI or smoking status were more likely to be young and male (eTable 2 in Supplement 3).

**Table 2.** Characteristics of the English and Danish Study Populations at Cohort Entry^a^

Variable	England		Denmark	
	With atopic eczema (n = 471 970)	Without atopic eczema (n = 2 239 775)	With atopic eczema (n = 44 945)	Without atopic eczema (n = 445 673)
Follow-up				
Total person-years	2 864 446	12 601 393	639 121	6 358 286
Duration of follow-up, median (IQR), y	4.8 (1.9-9.2)	4.2 (1.7-8.6)	14.3 (6.3-20.8)	14.3 (6.3-20.8)
Female sex	276 510 (58.6)	1 301 074 (58.1)	22 826 (50.8)	226 323 (50.8)
Age, y				
<18	NA	NA	31 772 (70.2)	316 396 (71.0)
18-44	262 119 (55.5)	1 292 565 (57.7)	9875 (22.0)	98 181 (22.0)
45-64	115 510 (24.5)	563 375 (25.2)	2418 (5.4)	23 308 (5.2)
≥65	94 341 (20.0)	383 835 (17.1)	880 (2.0)	7788 (1.7)
IMD quintile^b^				
1, Least deprived	113 598 (24.1)	531 707 (23.7)	NA	NA
2	107 613 (22.8)	509 274 (22.7)	NA	NA
3	92 864 (19.7)	441 451 (19.7)	NA	NA
4	90 112 (19.1)	428 193 (19.1)	NA	NA
5, Most deprived	67 783 (14.4)	329 150 (14.7)	NA	NA
BMI^c^				
Underweight, <20	34 065 (7.2)	165 212 (7.4)	NA	NA
Normal, 20-24	153 822 (32.6)	721 037 (32.2)	NA	NA
Overweight, 25-29	127 144 (26.9)	570 179 (25.5)	NA	NA
Obese, ≥30	83 169 (17.6)	341 122 (15.2)	NA	NA
Missing	73 770 (15.6)	442 225 (19.7)	NA	NA
Smoking^c^				
Nonsmoker	239 072 (50.7)	1 132 798 (50.6)	NA	NA
Current smoker or ex-smoker	220 331 (46.7)	975 355 (43.5)	NA	NA
Missing	12 567 (2.7)	131 622 (5.9)	NA	NA
Harmful alcohol use^d^	12 812 (2.7)	49 844 (2.2)	NA	NA
Lifestyle-related diseases				
Chronic obstructive pulmonary disease	NA	NA	555 (1.2)	1568 (0.4)
Hyperlipidemia	NA	NA	533 (1.2)	4389 (1.0)
Hypertension	NA	NA	2012 (4.5)	16 601 (3.7)
Alcohol-related conditions	NA	NA	591 (1.3)	4747 (1.1)
Ischemic heart disease	NA	NA	274 (0.6)	2011 (0.5)
Hospital-diagnosed obesity	NA	NA	474 (1.1)	3733 (0.8)
Type 2 diabetes	NA	NA	248 (0.6)	2325 (0.5)

Abbreviations: BMI, body mass index (calculated as weight in kilograms divided by height in meters squared); IMD, Index of Multiple Deprivation; IQR, interquartile range; NA, not applicable.

^a^ Unless otherwise specified, data are given as No. (%). Individuals could contribute data as both atopic eczema exposed and unexposed. Further characteristics for the English study population (including ethnicity and comorbidities) for the overall cohort, individuals included in the model additionally adjusting for potential mediators (ie, with no missing BMI or smoking status data), and for individuals with missing BMI or smoking status data are listed in eTable 2 in Supplement 3. Further characteristics for the Danish study population and socioeconomic variables (highest educational level, partner status, and gross personal income) among those 30 years or older are listed in eTable 3 in Supplement 3.

^b^ Based on individual-level data (from 2007) if available, supplemented with practice-level data (from 2010) if individual-level data were not available.

^c^ Based on records closest to the index date.

^d^ Based on records on or before cohort entry. We defined harmful alcohol use based on primary care morbidity codes suggesting harmful or heavy alcohol use (including alcohol dependency codes and codes associated with physical or psychological harm associated with alcohol use) or a prescription for drugs used to maintain abstinence (acamprosate calcium, disulfiram, or nalmefene hydrochloride).

In Denmark, the median follow-up was 14.3 years (IQR, 6.3-20.8 years). Distribution of age and sex was equal, and comorbidities at baseline were rare in both groups (Table 2). Among individuals aged 30 years at cohort entry, higher educational level and being single were more common in those with atopic eczema (eTable 3 in Supplement 3) than in those without atopic eczema.

### Main Analysis

In both countries, little evidence was found of any association between atopic eczema and overall cancer (Figure 2 and eTable 4 and eTable 5 in Supplement 3). Hazard ratios (HRs) comparing cancer risks in people with and without atopic eczema were close to 1, showing evidence of only a minor, clinically unimportant increase in minimally adjusted estimates (HR, 1.05; 99% CI, 0.95-1.16 in Denmark) and confounder-adjusted estimates (HR, 1.04; 99% CI, 1.02-1.06 in England). After further adjusting for potential mediators, estimates moved slightly closer to the null. For all specific cancer sites, only small differences in absolute cancer risk were seen between individuals with and without atopic eczema.

For most site-specific cancers, English and Danish analyses showed no strong evidence of an association with atopic eczema (Figure 2). For the following cancers, HRs comparing cancer risk in people with and without atopic eczema were close to the null: breast, prostate, pancreas, leukemia, multiple myeloma, and central nervous system cancers. There were decreases in melanoma risk and small increases in NMSC risk.

We found evidence of larger increases in lymphoma risk in people with atopic eczema compared with those without atopic eczema. In England, non-Hodgkin lymphoma (NHL) risk was 20% higher (HR, 1.20; 99% CI, 1.07-1.34) and Hodgkin lymphoma risk was 48% higher (HR, 1.48; 99% CI, 1.07-2.04) in people with atopic eczema compared with those without atopic eczema after adjusting for confounders (Figure 2). Although Danish point estimates for lymphoma were less precise than English estimates, they showed similar increased risk (minimally adjusted HR, 1.31; 99% CI, 0.76-2.26 for NHL and 1.35; 99% CI, 0.65-2.82 for Hodgkin lymphoma), but the 99% CIs were wide.

An initial small increased lung cancer risk was observed in people with atopic eczema vs those without (confounder-adjusted HR, 1.08; 99% CI, 1.01-1.16 in England and minimally adjusted HR, 1.45; 99% CI, 1.08-1.96 in Denmark) (Figure 2). However, risk attenuated after further adjusting for potential mediators (including smoking or smoking-related disease) (HR, 1.02; 99% CI, 0.95-1.10 in England and 1.21; 99% CI, 0.88-1.65 in Denmark) (eTable 5 in Supplement 3).

Our sensitivity analyses yielded similar results (including analyses restricted to individuals with a 3-year cancer-free window after eczema diagnosis to limit reverse causality as an explanation for our findings). These results are summarized in eTable 6 and eTable 7 in Supplement 3.

### Atopic Eczema Severity

**Figure 3.**
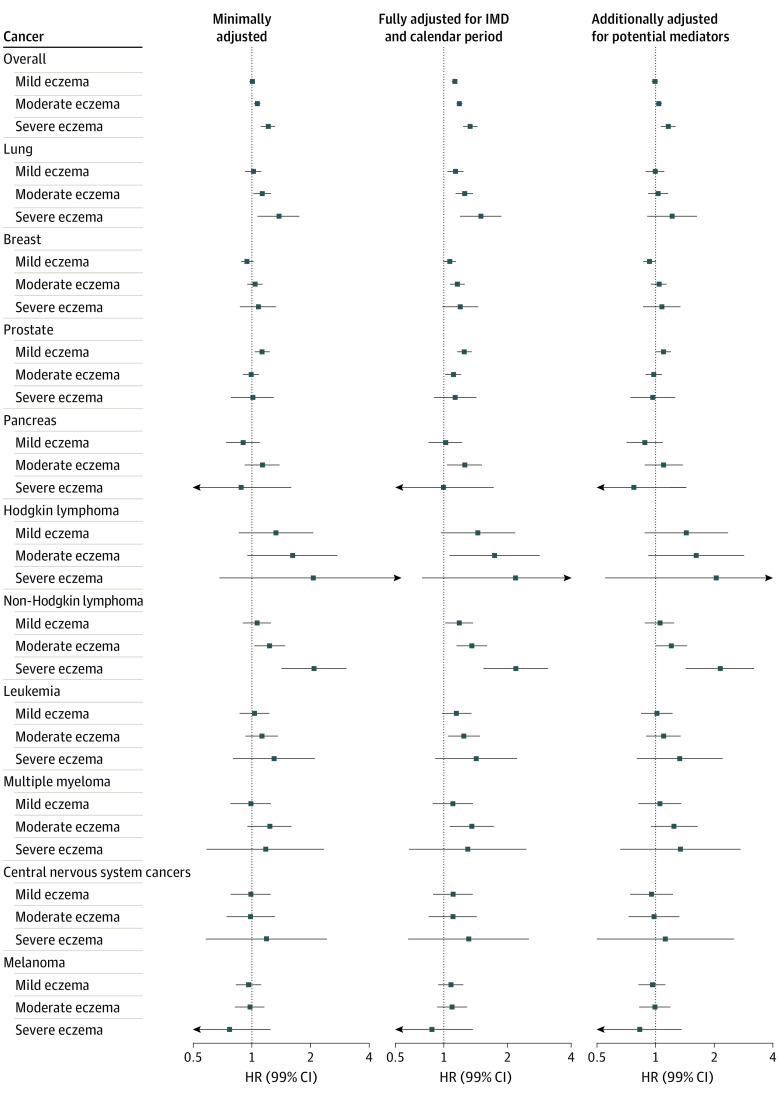
In England, Comparison of Cancer Rates at Each Level of Eczema Severity in People With vs Without Atopic Eczema Estimated hazard ratios are from Cox proportional hazards regression with current age as the underlying timescale, stratified by matched set (matched on age at cohort entry, sex, primary care practice, and date at cohort entry). All models were fitted to individuals with complete data for all variables included in each model and from valid matched sets, including 1 individual with atopic eczema and at least 1 individual without atopic eczema. All models implicitly adjusted for sex, primary care practice, and date at cohort entry because of stratification by matched set and for age because of the underlying timescale. Fifty-two percent of those with severe eczema were classified as having severe eczema based on a prescription for systemic drugs, 1.5% based on a record for phototherapy, 28.0% due to specialist dermatology referral, and 18.0% as a result of a hospital admission for atopic eczema. Potential mediators were body mass index, smoking, and harmful alcohol use. Specific central nervous system tumors (meningioma, brain neoplasm, and others) were too rare to consider individually. HR indicates hazard ratio; IMD, Index of Multiple Deprivation. Population counts are listed in eTable 4 and eTable 5 in Supplement 3.

In England, we generally found no evidence of different overall cancer or site-specific cancer risk in severe eczema (Figure 3 and eTable 8 in Supplement 3). An exception was lymphoma, particularly NHL, for which the observed increased risk became more pronounced with increasing eczema severity. The NHL confounder-adjusted HRs compared with those without atopic eczema were 1.06 (99% CI, 0.90-1.25) for mild eczema, 1.24 (99% CI, 1.04-1.48) for moderate eczema, and 2.08 (99% CI, 1.42-3.04) for severe eczema (eTable 8 in Supplement 3).

### Atopic Eczema Activity

In England, we saw limited evidence of an association between atopic eczema activity and most site-specific cancers (eFigure 3 and eTable 9 in Supplement 3). Notably, there were higher risks of NHL in people with more active atopic eczema (confounder-adjusted HR compared with those with no atopic eczema, 1.07; 99% CI, 0.83-1.38 for never active eczema; 1.08; 99% CI, 0.92-1.26 for moderately active eczema; and 1.58; 99% CI, 1.28-1.94 for very active eczema) and higher overall cancer risk in people with more active eczema (confounder-adjusted HR, 1.11; 99% CI, 1.07-1.15) (eTable 9 in Supplement 3).

### Effect Modification

Little evidence was found of effect modification by age, sex, or presence of asthma. These results are summarized in eTables 10, 11, 12, 13, 14, and 15 in Supplement 3.

## Discussion

In 2 matched cohort studies from England and Denmark, no evidence was found of an association between atopic eczema and most cancers. However, increased risk of lymphoma, particularly NHL, was seen among people with atopic eczema, specifically those with more severe or active disease. In England, we observed a 23% increased risk of lymphoma overall (similar to Danish estimates), with a 100% increase in NHL risk in people with severe eczema, although absolute risk differences were small.

### Findings in Context

A recent systematic review and meta-analysis^12^ of cancer risk in people with atopic eczema highlighted the heterogeneity and limitations of existing data. Overall, our findings generally confirm those reported in prior smaller studies.^12,14,16,19,23,46,47,48,49,50,51,52,53,54^

Our study is one of the few longitudinal studies^12,14,16,23,46,47,52^ to adjust for numerous potential confounders and mediators and to address the role of eczema severity and activity in 2 cohorts with complementary strengths and limitations. Our results suggest that the overall cancer risk among individuals with atopic eczema is small. This baseline risk is particularly important to establish as many new immunomodulatory systemic therapeutics are brought to market.

Previous studies assessing the association between atopic eczema and site-specific cancers have had conflicting results.^12^ A discussion follows for brain cancer, solid-organ cancers, melanoma, and noncutaneous lymphomas.

#### Brain Cancer

A recent systematic review and meta-analysis^46^ reported a 23% reduction in brain cancer risk in people with atopic eczema. However, this protective association was not observed with more specific atopic eczema diagnostic criteria, and there was substantial heterogeneity (*I*^2^ > 75%) between studies. We could not reliably assess the risk of brain cancer subtypes because of low statistical power.

#### Solid-Organ Cancers

Some prior cohort and case-control studies^47,48,49,50,51^ that assessed other solid-organ cancers found an increased risk of lung cancer associated with atopic diseases. Although we initially observed a small increased lung cancer risk in individuals with atopic eczema, this association disappeared after adjusting for possible mediators, suggesting that the increased risk may be attributable to smoking among people with atopic eczema.

#### Melanoma

The observation of a potentially protective association of atopic eczema with melanoma is of interest. Our findings are consistent with observations from mouse models^21^ and may reflect sun avoidance in people with atopic eczema or increased immune surveillance (further studies would be required to elucidate biologic mechanisms). Having atopic eczema might have led to more skin examinations, resulting in ascertainment bias and either an increased skin cancer risk (as seen for NMSC in this study and in other studies^23,47,51,52^) or an apparently decreased risk because of better detection of melanoma in situ (with consequent lower invasive cancer risk). If ascertainment leads to increased melanoma detection, we would expect to see an even larger risk reduction in advanced melanoma; additional studies could examine this hypothesis.

#### Noncutaneous Lymphomas

The association between atopic eczema and lymphoma is consistent with previous findings in a systematic review and meta-analysis^16^ of an overall relative risk of lymphoma of 1.43, as well as the finding in a CPRD-based case-control study^19^ of an association between Hodgkin lymphoma and allergic disease (asthma, eczema, and allergic rhinitis). The association of eczema severity with lymphoma has previously been assessed in only 1 cohort study^55^ and 2 case-control studies.^53,54^ Although the cohort study^55^ used data from Medicaid, a US administrative database, and focused on psoriasis, it demonstrated a 2-fold increased lymphoma risk among individuals with severe eczema in a limited secondary analysis. However, because NHL and Hodgkin lymphoma have different risk factor profiles,^56^ our findings in this study might suggest detection bias (because of the use of chest radiographs in people with comorbid asthma) or residual confounding, perhaps because of immunosuppression^57^ (although sensitivity analyses adjusting for immunosuppression in the present study were similar to our main analysis results).

Previous studies attempting to clarify whether topical calcineurin inhibitors (used in our study to identify moderate eczema severity) increase lymphoma risk in people with atopic eczema have failed to convincingly demonstrate an association, although most analyses were limited by low statistical power and confounding by severity.^16^ A low proportion of individuals with atopic eczema treated in primary care receive calcineurin inhibitors because these agents are recommended for specialist use only.^58^

### Implications for Future Research

In this study, we found an increased lymphoma risk in individuals with atopic eczema, especially for those with severe eczema; however, absolute differences were small, and there was little evidence of an increased risk of other cancers. This finding may be because of reverse causation (ie, atopic eczema could be an early sign of lymphoma), but a sensitivity analysis extending the cancer-free lag period to 3 years did not show any evidence of this hypothesis (eTable 6 in Supplement 3). Future research could explore underlying mechanisms for the associations observed for lymphoma, particularly NHL, including studies assessing Epstein-Barr virus in people with atopic eczema, as well as addressing the therapeutic implications (associated with existing and novel immunosuppressive treatments) of our findings in individuals with severe eczema.

### Strengths and Limitations

This study has strengths and limitations. A major strength of this study is the use of prospectively collected data from large representative population-based databases from 2 countries with free health care access.^26,30,59^ Our results reflect real-world clinical practice and are likely to be generalizable to other settings. Although the Danish findings are unlikely to apply to individuals with mild eczema because the Danish data were restricted to hospital-based atopic eczema diagnoses, the Danish results are consistent with those for individuals with moderate to severe eczema in England. Therefore, combining the English and Danish population-based data lends credence to the study conclusions by virtue of triangulation.^60^

It is possible that atopic eczema was misclassified in our study. Although we used a validated algorithm to identify atopic eczema in England and the validity of diagnoses in a dermatology department was found to be high in Denmark (positive predictive value, 99%^61^), we may have missed atopic eczema diagnoses (likely unrelated to cancer risk), which could have underestimated the magnitude of any association between atopic eczema and cancer.

The quality of diagnoses in the Danish Cancer Registry is excellent: reporting is mandatory, diagnoses stem from a range of sources, the proportion of histologically verified tumors is high (>90% for major cancers), and few diagnoses are based only on death certificates (0.1%).^32,62,63^ In England, most nationally registered cancer cases (>90%) can be identified in the CPRD; by including the HES and ONS data, we further improved cancer identification.^64^ Nevertheless, we cannot exclude ascertainment bias; cancers may be diagnosed more frequently in individuals with atopic eczema because of frequent skin examinations for skin cancer, regular blood test monitoring for systemic immunosuppressive drugs (in severe eczema only), or chest radiographs for concomitant asthma or before drug initiation in severe eczema (eg, cyclosporine).

Potential misclassification of covariates may have led to residual confounding, biasing the estimates of direct associations in mediation analyses. However, increasing adjustment made little change to our HR estimates. In Denmark, we were only able to adjust for potential confounders (ie, socioeconomic status) in sensitivity analyses, but the results of these sensitivity analyses were similar to those of the main analysis. However, matching with replacement in the Danish cohort may have resulted in mild misleadingly narrow 99% CIs.

A limitation that is common to many dermatological conditions lacking quantitative measures is reliance on treatments to classify disease severity and activity. In Denmark, sample sizes were insufficient for eczema severity and activity analyses; however, we note that, by definition, most individuals had moderate to severe eczema. Atopic eczema was identified in hospital records because we had no access to data from Danish primary care, where mild eczema is managed. The study also had limited power to examine certain cancers, specifically brain cancer subtypes.

## Conclusions

In this study, no evidence was found that people with atopic eczema are at increased risk of most cancers. An exception is the observed association between atopic eczema and lymphoma, particularly NHL, that increased with eczema severity. This finding warrants further study as new immunomodulatory systemic therapeutics are brought to market that may alter cancer risk.

## Notes

Supplement 1. English Trial Protocol

Supplement 2. Danish Trial Protocol

Supplement 3. **eAppendix 1.** Variable Definitions **eAppendix 2.** Study Populations **eAppendix 3.** Secondary Analyses
eReferences.
eFigure 1. England: Flowchart Illustrating Identification of Study Participants eFigure 2. Denmark: Flowchart Illustrating Identification of Study Participants eFigure 3. England: Association (HR [99% CI]* Comparing Individuals at Each Level of Eczema Disease Activity to Those Without Eczema) Between Atopic Eczema Disease Activity and Cancer eTable 1. Sensitivity Analyses eTable 2. England: Characteristics of the Study Population at Cohort Entry, for: the Overall Cohort, Individuals Included in the Model Additionally Adjusting for Potential Mediators (i.e. With No Missing BMI or Smoking Status Data), and for Individuals With Missing BMI or Smoking Status eTable 3. Denmark: Characteristics of the Study Population at Cohort Entry, for the Overall Cohort and for Individuals Aged 30 Years or Over (Included in Sensitivity Analysis Adjusting for Socioeconomic Status) eTable 4. England: Association (HR [99% CI]) Between Atopic Eczema and Cancer Outcomes: Comparing Risk of Cancer in Those With Atopic Eczema to Those Without eTable 5. Denmark: Association (HR [99% CI]) Between Atopic Eczema and Cancer Outcomes: Comparing Risk of Cancer in Those With Atopic Eczema to Those Without eTable 6. England: Hazard Ratios (99% CIs)* for Main and Sensitivity Analyses (After Adjusting for Calendar Period and IMD) eTable 7. Denmark: Hazard Ratios (99% CIs) for Main and Sensitivity Analyses eTable 8. England: Association (HR [99% CI]*) Between Severity of Atopic Eczema and Cancer Outcomes eTable 9. England: Association (HR [99% CI]*) Between Atopic Eczema Disease Activity and Cancer Outcomes eTable 10. England: Adjusted Hazard Ratios (99% CIs) for the Association Between Atopic Eczema and Cancer, Stratified by Sex (Adjusted for Calendar Period and IMD) eTable 11. Denmark: Adjusted Hazard Ratios (99% CIs) for the Association Between Atopic Eczema and Cancer, Stratified by Sex eTable 12. England: Adjusted Hazard Ratios (99% CIs) for the Association Between Atopic Eczema Stratified by Current Age (Adjusted for Calendar Period and IMD) eTable 13. Denmark: Adjusted Hazard Ratios (99% CIs) for the Association Between Atopic Eczema and Cancer, Stratified by Age eTable 14. England: Adjusted Hazard Ratios (99% CIs) for the Association Between Atopic Eczema Stratified by Asthma (Adjusted for Calendar Period and IMD) eTable 15. Denmark: Adjusted Hazard Ratios (99% CIs) for the Association Between Atopic Eczema and Cancer, Stratified by Asthma eTable 16. Denmark: Codes Used to Define Atopic Eczema eTable 17. Denmark: Codes Used to Define Outcomes (Cancer Overall and Specific Cancer Outcomes) eTable 18. Denmark: Codes Used to Define Covariables (Confounders and Mediators)
