# Supplementary material for: Association Between Atopic Eczema and Cancer in England and Denmark
Source: JAMA Dermatol. 2020 Jun 24;156(10):1086–97. doi: 10.1001/jamadermatol.2020.1948 (PMC7315391; doi:10.1001/jamadermatol.2020.1948)
Supplement: Supplement 1. — English Trial Protocol [file jamadermatol-e201948-s001.pdf]

The association between eczema and cancer: a cohort study
Summary protocol

1. Background
The association between eczema and risks of specific cancers is an ongoing area of controversy, with conflicting findings. Many studies report reductions in specific brain cancers and increased risk of lymphomas.[1–8] Two competing theories are proposed for the complex association between eczema and cancer: immune surveillance (reduced risk) and immune stimulation (increased risk). Immunosuppressive therapy (including topical calcineurin inhibitors) and an impaired skin barrier may also increase risks, particularly of skin cancers, although current findings are inconclusive, and a very low proportion in primary care will have received calcineurin inhibitors as they are recommended for hospital use only. Recent experimental models support reductions in risk of skin cancer in patients with eczema, although data from population-based studies are conflicting.[2,9–12]

2. Aims and objectives
The objectives of this study are to use routine primary care data from the Clinical Practice Research Datalink (CPRD), linked hospital admissions data from hospital episode statistics (HES) and cause of death data from the Office for National Statistics (ONS) to examine the associations between eczema and cancer. Specifically, we aim to:

1. Determine if eczema is associated with increased risk of all cancers and a number of specific cancers (by calculating hazard ratios for the association between eczema and cancer).
2. Establish if cancer is more likely in individuals with active or severe eczema.
3. Explore whether the following modify the effect of eczema on cancer:
    - Age
    - Sex
    - Asthma
    - Specific systemic immunosuppressive treatments (including cyclosporine or azathioprine).

2. Study population
We will identify a matched cohort of adults aged over 18 years registered with CPRD practices eligible for linkage to HES/ONS data between January 1998 and March 2016 (period of complete linkage with HES and ONS version 14). All individuals will need to be registered with practices meeting CPRD quality control standards, have at least 12 months of registration prior to study entry (to allow adequate time for recording of baseline data in the patient record), and no previous history of cancer (or specific cancer diagnosis for individual cancer outcomes).

We will follow individuals until the earliest of: no longer registered with practice, practice no longer contributing data to CPRD, death, end of study (March 2016) or cancer diagnosis (any cancer diagnosis or specific cancer diagnoses for individual cancer outcomes).

2.1 Eczema exposed
Individuals exposed to eczema will be identified using a validated algorithm[13] based on a record of one diagnostic morbidity code (recorded in either primary or secondary care) and at least two records for eczema therapy (recorded in primary care using Read morbidity codes or prescription data). Eczema-exposed individuals will enter the cohort on the latest of: date of registration with GP practice; date practice met CPRD quality control standards; start of study (2nd January 1998); 18th birthday; or 12 months from date of eczema diagnosis (i.e. date the individual meets the eczema algorithm of one diagnostic code and two records for eczema therapy – latest of eczema morbidity

code or second eczema therapy record). We have allowed a 12-month window from date of eczema diagnosis to limit reverse causality as a reason for any observed association.

2.2 Eczema unexposed

We will identify an age, sex, calendar period and general practice matched cohort of individuals without eczema (the importance of matching here is in relation to general practice, since we cannot adjust for general practice, age is less important as we will adjust for this in all analyses). We will randomly match (with replacement) up to five individuals without eczema for every individual with eczema. Individuals without eczema will enter the cohort on the same date as their matched eczema-exposed individual.

After initial work we have decided to allow a 15-year difference in age for age matching in order to maximise the generalisability of our cohort. Initial work suggests that we will have a sufficient power if we use a narrower age window for matching (i.e. 5 years gives 7% unmatched exposed individuals compared to 4% unmatched using a 15-year window) but, the eczema exposed individuals who are unmatched are more likely to be older and female when compared to those who were matched (Preliminary results, allowing for a 15-year age difference when matching: Mean year of birth for eczema-exposed people *not* matched was 1967 while that for those who *were* matched is 1961. Further, 72% of those who were unmatched were female, compared to 59% who were matched). Therefore, given that we will adjust for age, we opted for a wider age-matching window in order to identify a cohort who are more likely to be representative of all those with eczema.

3. Exposure

As outlined above, individuals will be defined as having eczema based on an existing algorithm requiring a record of at least one eczema morbidity code and at least two eczema therapy records. Once a participant fulfils the algorithm they will be considered exposed.

We will also identify severe and active eczema for use in secondary analyses (see **Section 6.2.1**).

3.1 Eczema severity

We will classify eczema severity as mild, moderate or severe. Atopic eczema patients will be considered to have mild disease by default. They will be classified as having ‘moderate’ atopic eczema at the first of: 1) their second potent topical corticosteroid treatment within one year; or 2) their first topical calcineurin inhibitor (both topical corticosteroid and calcineurin treatment will be identified using primary care prescribing data). Finally, individuals will be classified as having severe eczema if: 1) they have received either phototherapy or systemic treatment (excluding systemic corticosteroids in this definition as these treatments are likely to be given for associated asthma) for eczema; 2) they have been admitted to hospital with eczema as the primary reason for admission (i.e. where eczema is recorded in the primary diagnostic position for any episode of an admission); or 3) they have a referral to a dermatologist. Phototherapy will be identified using Read coding in primary care and OPCS procedure coding in secondary care. Systemic eczema treatment will be identified using primary care prescribing records. Hospital admissions for eczema will be identified as any hospitalisation in which an ICD-10 code for eczema is recorded in the primary diagnostic position of any episode of an admission.

Atopic eczema severity will be defined as a time-updated variable. We will continue to classify an individual as having moderate eczema from date they first meet the moderate-eczema definition (unless they develop severe eczema). Similarly, we will continue to classify an individual has having severe eczema from the point at which they meet the definition for ‘severe eczema’ (similar to established approaches for defining severity in psoriasis studies[14]). At any given point during follow-up, atopic eczema patients will therefore belong to one of three severity categories: mild, moderate, or severe.

3.2 Active eczema

We will define active eczema when a patient has two or more primary care consultations, primary care prescriptions, or hospital records for eczema within a one-year period. Thus, a person will start contributing person-time in the "active eczema" group on the date of the second consultation or prescription. We will then classify individuals as having active eczema for the next 12 months, unless a new consultation/prescription is recorded, in which case we will classify them as having active eczema for a further 12 months (and so on). Periods of disease activity will be identified irrespective of disease severity.

4. Outcome

We will use morbidity coding recorded in CPRD (Read codes) and HES (ICD-10 codes), and cause of death coding in ONS data (ICD-9 pre Jan 2001 and ICD-10 post Jan 2001) to identify cancer diagnoses. Cancer code lists will be updated from our group's recent publications in this field.[15] Codes on the existing 'all cancers' Read code list are classified as: borderline/unclear; in-situ; cancer/malignancy; secondary; suspected malignancy; code indicative of malignancy; and code indicative of chemo/radiotherapy. In order to identify codes representing clear cancer diagnoses, we will only include codes classified as cancer/malignancy or secondary cancer. In a sensitivity analysis we will additionally include codes classified as indicative of malignancy or chemo/radiotherapy.

Over 90% of nationally registered cancers can be identified in CPRD records. By additionally including ONS and HES data we hope to further improve our ability accurately identify cancer.[16]

We will exclude non-melanoma skin cancers from the all cancers outcome as we believe that these are likely to be recorded poorly; these cancers are often treated with photodynamic therapy and consequently there is often no biopsy specimen available with which to confirm a diagnosis. We are also concerned about introducing ascertainment bias by including any skin cancer in the all cancers outcome; individuals with eczema are more likely to have a skin cancer diagnosis due to increased scrutiny of their skin by clinicians. In order to explore the impact of this potential ascertainment bias we will conduct a sensitivity analysis by repeating the all-cancer analysis after excluding all skin cancer from the outcome definition.

We will explore the following specific cancers as outcomes: (1) lung; (2) breast; (3) prostate; (4) pancreatic; (5) skin – classified initially as melanoma vs non-melanoma (keratinocyte cancer), with further strata if possible; (6) haematological – classified as lymphoma, multiple myeloma, or leukaemia; and (7) central nervous system (CNS) cancers – we will initially look at all CNS cancers as a composite outcome and then, because the existing literature suggests it is appropriate, stratified as: meningioma, brain neoplasm (glioma), and spinal cord, cranial nerve or other CNS tumours. We have chosen these specific cancers as they are among the most common cancers in the UK. In addition, some specific cancers have been shown to be associated with eczema/atopy: In a meta-analysis of atopy and CNS tumours, atopy was shown to be associated with reduced risk of glioma, but no overall risk of meningioma,[4] however other studies have seen an association between eczema and increased risk of meningioma; eczema has been associated with increased risk of pancreatic cancer.[1]

Previous work by members of our research group has mapped ICD-10 codes for specific cancers to Read codes (Table 1).[15] We will use this Read coding mapping (alongside corresponding ICD-10 codes recorded in HES and ICD-9/ICD-10 codes recorded in ONS data).

Table 1. Specific cancer outcomes and ICD-10 codes.

Specific cancer	ICD-10 code(s)	Notes
Lung	C34	
Breast	C50	
Prostate	C61	
Pancreatic	C25	
Skin		We are aware that looking at skin cancer in eczema exposed/unexposed will lead to ascertainment bias (people with eczema are more likely to be diagnosed with skin cancer as clinicians will be examining skin more carefully in those with existing skin disease). We will therefore need to take care with these analyses.
Melanoma	C43	
Non-melanoma	C44	
Haematological		
Lymphoma	C81-86	We will make no distinction between Hodgkin's and non-Hodgkin's lymphoma (but will stratify if power allows)
Multiple myeloma	C90	
Leukaemia	C91-95	
CNS		
Meningioma	C70	
Brain neoplasm (glioma)	C71	
Spinal cord, cranial nerve or other CNS tumours	C72	

CNS: central nervous system

5. Covariates

We will consider the following as potential covariates: age, sex, calendar period), lifestyle factors (smoking, body mass index [BMI], and alcohol intake), and index of multiple deprivation (as a proxy for socioeconomic status).

For descriptive analyses, age will be classified into 10-year age bands (for Cox regression, age will be used as the underlying timescale). However, when investigating some specific cancers as outcomes we may need to define finer bands in specific age groups, this will be guided by reviewing existing literature for age distribution in specific cancers.

Lifestyle covariates (smoking, alcohol and BMI) will be defined based on primary care records for these measures recorded closest to study entry date. The identification of lifestyle variables will be pragmatically based on status recorded closest to the index date, with records within -1 year to +1 month from index date regarded as the best, +1 months to +1 years from index date being second best, the nearest before -1 year from index date as the third best, and within +1 year from index date being the worst. Smoking status will be classified as: current-/ex-smoker or non-smoker. Alcohol consumption will be classified as: current-, ex- or non-drinker. Body mass index will be classified using World Health Organisation categories (underweight (<18.5 kg/m2), normal weight (18.5–24.9 kg/m2), overweight (25–29.9 kg/m2), obese (≥30 kg/m2)). Read codes for BMI category will not be used, because they are rarely recorded.

We will use index of multiple deprivation as a proxy for socioeconomic status. IMD will be measured using quintiles of patient-level IMD scores linked via the practice and patient postcode (where patient-level data is available for patients eligible for linkage to IMD data). We will use the 2007 version of IMD data (IMD data is available for the years: 2004, 2007, 2010 and 2015), we have chosen this as the midpoint of the study (January 1998 to March 2016).

In all models, GP practice will be dealt with through matching.

In addition, we will also consider immunosuppression, diabetes mellitus, use of oral corticosteroids, frequent attender status (as a proxy measure of health-seeking behaviour), and ethnicity as covariates in sensitivity analyses (see **Section 6.3**).

6. Analysis

6.1 Main analysis

We will present descriptive statistics comparing the following variables between individuals with eczema and those without: age, sex, lifestyle factors (smoking, alcohol and BMI), diabetes mellitus, high-dose oral corticosteroid use, IMD, asthma, and immunosuppression.

We will use Cox regression (99% confidence intervals) to estimate the hazard ratio for the association between eczema and cancer, adjusting for covariates (see modelling strategy in **Table 2**), accounting for matching. We will exclude participants with specific (or any, depending on the analysis) cancer prior to study entry. We will use age as the underlying time scale in order to adjust for changes with age over time more finely (this will not allow us to reviewing changing trends with age, so, should we feel this is important, we will repeat the main analysis using calendar time as the underlying timescale with age band as a covariate, in a sensitivity analysis).

We will be using 99% confidence intervals in order to reduce the type 1 error introduced by including multiple outcomes.

Table 2. Modelling strategy for main analysis

Model 1a	(Model 1b)	Model 2
Age<br>Sex<br>Calendar period	Smoking<br>Alcohol<br>BMI<br><br>Ideally lifestyle covariates will be included in Model 1 – however, if ++ missing data we will include in a sensitivity analysis only	IMD*<br><br>As for Model 1 + IMD. It's likely that IMD will be captured by some of the covariates included in the main analysis, therefore we will add separately in a second model.

BMI: body mass index; IMD: index of multiple deprivation
*We may include IMD in main model if there is a substantial change in effect estimates between Models 1 and 2 (i.e. if IMD is not captured by lifestyle covariates / chronic comorbidities).

6.2 Secondary analyses

We will conduct a number of secondary analyses aiming to investigate whether: (1) cancer is more likely in individuals with active or severe eczema; and (2) the effect of eczema on risk of cancer is modified by age, sex, asthma or specific systemic immunosuppressive treatments (including cyclosporine or azathioprine).

We will explore whether the association between eczema and cancer differs by eczema severity or eczema disease activity by stratifying separately on disease severity (defined in **Section 3.1**) and activity (defined in **Section 3.2**).

We will explore whether age, sex and asthma modify the effect of atopic eczema on cancer outcomes by stratifying results for each sex and age category and whether individuals have asthma or not. Where appropriate, we will fit models that adjust for age, sex and additional confounders, with interaction terms between eczema and age/sex or asthma status allowing us to assess whether the association of eczema with cancer varies with age and sex or asthma.

We will also explore if the effect of eczema on cancer is modified by specific systemic therapies by drug class (systemic immunosuppressive treatments, i.e. cyclosporin, azathioprine, mycophenolate and methotrexate). Broadly speaking, these analyses will only be in patients with severe disease. For

these analyses, we will classify eczema patients into those who have ever-received or never-received the specific treatment to determine if any association between eczema and each outcome is restricted to those on the specific systemic drugs. Subsequent analyses will further divide the eczema cohort into different treatment groups and then this will be treated as a categorical variable to test if the association is the same in each group. However, as a very low proportion of individuals with eczema ever receive systemic treatment (97% of individuals are managed in primary care), these analyses are likely to be underpowered. Further, another limitation of this analysis is that we will be unable to capture records for systemic therapies prescribed in secondary care. We will therefore misclassify a number of individuals as never having been exposed to a systemic therapy when in fact they received their systemic treatment in secondary care. We hope that only a small number of individuals will be affected by this as it is unlikely that an individual with eczema of sufficient severity to require systemic therapy will never have a primary care prescription for that therapy.

6.3 Sensitivity analyses

We will test how robust our findings are by repeating the main analysis after systematically altering aspe*cts* of the main study design in a series of sensitivity analyses outlined in Table 3.

Table 3. Sensitivity analyses.

No.	Sensitivity analysis	Justification
1	We will repeat the main analysis requiring a **3 year cancer-free window** following eczema diagnosis (main analysis requires a 12-month window). If we see a large change at 3 years, we will also repeat the main analysis requiring a 5 year cancer-free window.	We have included a 12-month cancer-free window following eczema diagnosis to limit **reverse causality** as an explanation for any association observed, by increasing this to 3 years we hope to account for any time lag between onset of cancer and diagnosis and therefore further reduce reverse causality.
2	The main analysis will be repeated on an **incident atopic eczema cohort** (exposed patients defined as those joining the cohort when they first fulfil our diagnostic criteria and after the start of the study period).	**Covariates measured at entry precede atopic eczema** onset so will not be on the causal pathway between atopic eczema and cancer outcomes.
3	We will repeat the main analysis additionally adjusting for **immunosuppression**. Our definition of immunosuppression will include: immunosuppressive disorders, drugs leading to immunosuppression (excluding oral corticosteroid use, as this is likely to be transient), and lymphopenia.<br><br>**Immunosuppressive disorders** will include: HIV; haematopoietic stem cell or bone marrow transplant; myeloma or other unspecified cellular immune deficiencies (e.g. pancytopenia); recent history (less than 2 years before start of follow-up) of leukaemia; and lymphoma. Myeloma, leukaemia, and lymphoma will be removed as covariates from the respective analyses in which they are the outcome. Recent history of leukaemia (within 2 years before index date) will be recorded in relation to index date (i.e. study entry date).<br><br>**Lymphopenia** will be defined using the most recent blood test result recorded in primary care prior to study entry (entity type 208 in CPRD test data). We will define lymphopenia based on a low lymphocyte count (i.e. <1 x 109/L) recorded as part of the most recent blood test recorded prior to study entry (NB, previous research [unpublished] by our group has demonstrated little difference between eczema exposed and unexposed in terms of frequency of lymphocyte count records). We will not include lymphopenia in our composite measure of immunosuppression, as a single record of lymphopenia may represent a transient event. We will therefore conduct an exploratory analysis to investigate whether lymphopenia confounds any relationship between atopic eczema and cancer here. If lymphopenia does appear to act as a confounder in this context we will develop additional formal analyses (as part of a separate protocol) to explore this further.	As immunosuppression might mediate the relationship between eczema and specific cancers, we will only adjust for immunosuppression in a sensitivity analysis.
4	The main analysis will be repeated on patients with **at least one consultation** with their GP in the year prior to cohort entry.	To exclude practice **non-attenders.**

No.	Sensitivity analysis	Justification
5	The main analysis will be repeated on a **redefined cohort** (different unexposed pool, exposed individuals unchanged, unless unmatched), where: 1) the pool of unexposed persons also includes patients with an atopic eczema diagnosis but without two further treatments for the entire duration of their follow-up; and 2) patients in the exposed cohort (with an atopic eczema diagnosis and two further treatments) will be classified as unexposed up until their cohort entry (i.e. the latest of their atopic eczema diagnosis and their two further treatments). This cohort will be matched separately to the main analysis cohort.	To explore the sensitivity of the results to the **definition of the exposure.**
6	The main analysis will be repeated on a **second redefined cohort** where exposed patients will be those with an atopic eczema diagnosis only (i.e. without requiring two atopic eczema treatments), and these patients will be eligible for inclusion in the unexposed cohort up until their atopic eczema diagnosis (some patients may have childhood atopic eczema, but may not have treatment codes recorded if registered at GP during adulthood, and therefore may be erroneously excluded from the exposed cohort in the primary analysis), This cohort will be matched separately to the main analysis cohort.	To explore the sensitivity of the results to the **definition of the exposure.**
7	The main analysis will be repeated on a subset of patients **registered from 2007** onwards.	Data on covariates, particularly **BMI and smoking**, would be expected to be more complete, thus reducing any selection bias due to missing data.
8	The main analysis will be repeated on a subset of patients **registered from 2006 onwards**, additionally adjusting for **ethnicity** (White, South Asian, Black, other or mixed, identified from CPRD and HES data using a previously developed algorithm[17]). We will include ethnicity as a covariate in those joining the cohort from 2006 onwards (more complete records for ethnicity following the introduction of remuneration for recording of ethnicity data in the Quality and Outcomes Framework).	To examine whether the omission of ethnicity as a covariate in the main analysis may have introduced bias.
9	We will repeat the main analysis including **frequent attender status** as a covariate. However, it is difficult to capture a propensity-to-consult measure effectively using consultation data, and we have yet to develop this measure. One approach would be to calculate median yearly consultation frequency over total follow-up for each individual after excluding consultations for eczema or cancer.	To limit the influence of ascertainment bias (a particular issue when skin cancer is the outcome). Individuals with eczema are likely to consult their health care providers more frequently than those without and therefore have more opportunity for a cancer diagnosis.

No.	Sensitivity analysis	Justification
10	We will repeat the main analysis with the **outcome all cancer** defined using codes classified as representing cancer/malignancy and secondary cancer (as in the main analysis) in addition we will also include codes classified as indicative of malignancy or codes for chemo/radiotherapy.	We have chosen to only include codes that clearly diagnose cancers in the main analysis, however, this definition may miss some cases (**outcome misclassification**). It is unlikely that any cases we miss will be differential between eczema exposed and unexposed, so it is unlikely that this will influence our effect estimate. However, it may limit the power of our study, we will therefore repeat the main analysis with a more inclusive definition of the all cancer outcome.
11	We will repeat the all cancers analysis after **excluding any skin cancer diagnoses** from the **all cancer** outcome definition.	To explore potential **ascertainment bias** introduced by investigating skin cancer in eczema exposed/unexposed; people with eczema are more likely to be diagnosed with skin cancer as clinicians will be examining skin more carefully in those with existing skin disease.
12	We have discussed that a **competing risks analysis** may be appropriate in this setting. We have yet to make a firm decision regarding how to approach this. Further consideration is needed.	
13	The analysis stratified by **eczema activity** will be repeated, restricted to patients with **at least 5 years of follow-up**.	To explore any potential bias caused by atopic eczema patients with **short follow-up periods** being more likely to have either none or all of their follow-up with **active atopic eczema.**
14	Repeat main analysis using a cohort that has been matched **without allowing for a 12-month cancer-free window** from eczema diagnosis date (i.e. start of observation for eczema-exposed will be the earliest of: diagnosis date + one year, current registration date + one year, up-to-standard date, or 18th birthday).	In order to be comparable with **Danish** cohort.
15	We will repeat the main analysis restricting to those entering **the cohort from 2004 onwards**.	To account for changes in diagnostic and coding practices over time – specifically those introduced by the Quality and Outcomes Framework in 2004 (likely to be more important for cancer outcomes and some covariates, but there are no specific dermatology indicators in the Quality and Outcomes Framework, so unlikely to affect eczema coding).

No.	Sensitivity analysis	Justification
16	We will repeat the main analysis including **diabetes mellitus** as a covariate. Diabetes mellitus will be classified as: absent, type 1, type 2, or type unknown, and will be defined based on primary or secondary care diabetes diagnoses (using definite diagnostic codes only). Onset of diabetes will be defined as date of the earliest diagnostic code recorded. Diabetes mellitus is part of the Quality and Outcomes Framework, which remunerates primary care practices for recording of specific management and monitoring practices in some chronic conditions. Therefore, diagnostic coding is likely for those with diagnosed diabetes (therefore no need to supplement the definition with anti-diabetic prescribing or possible diabetes codes.	Our rationale for including diabetes mellitus as a covariate and stratifying by diabetes type (rather than simply classifying as present or absent) is as follows: 1) type 1 diabetes mellitus is immune-mediated (resulting from autoimmune destruction of pancreatic beta-cells); 2) research suggests that there is a link between diabetes and cancer17; 3) we believe that any link between eczema and cancer will be via an immune mechanism; therefore, it seems important to consider diabetes as a potential confounder in the relationship between eczema and cancer. However, as this is a somewhat tenuous link we will only adjust for diabetes in a sensitivity analysis.
17	We will repeat the main analysis additionally adjusting for use of **oral corticosteroids**. As oral corticosteroids may be given transiently and intermittently it may be difficult to attribute a causal association with an outcome (i.e. cancer) that may occur many years after initial exposure (eczema). We will therefore restrict our definition of oral corticosteroid use to high-dose oral corticosteroid use. High-dose steroids will be defined as a daily dose of prednisolone of 20mg or more (the doses of other corticosteroid drugs will be converted to prednisolone equivalent dosage). As no one definition of oral corticosteroid use using electronic health record data is ideal,[18] we will define high-dose corticosteroid exposure in two ways:<br>1. **Never/ever:** Individuals classified as exposed if they ever have a prescription for a high-dose oral corticosteroid, with status changing at the first prescription for a high-dose corticosteroid (i.e. the variable will be time-updated). The problem with this approach is that we will classify some individuals as ever having been exposed to a high-dose steroid on the basis of a one-off prescription. We will therefore end up with a heterogeneous group of individuals including those who have had one rescue course of steroids and those who are on long-term high dose steroid.<br>2. **Percentage of follow-up time on high-dose corticosteroid:** Here we will identify the proportion of total follow-up when an individual is prescribed a high-dose steroid (the total number of days prescribed a high-dose steroid / total number of days of follow-up). However, the problem with this definition is that those with a very short duration of follow-up, prescribed a short course of steroids will have a large percentage of time exposed. To avoid this we could exclude those individuals with less than one year of follow-up.	As immunosuppression due to oral corticosteroids may mediate the relationship between eczema and specific cancers and high-dose oral corticosteroid use may act as a proxy for eczema severity, we will only adjust for immunosuppression in a sensitivity analysis.
18	We will consider repeating our analyses after restricting to **individuals resident in specific geographical areas** that are predominantly white in order to account for ethnicity.	We plan an analysis adjusting for ethnicity (**Sensitivity analysis 8**) however, this analysis will be limited to those joining the cohort from 2006 onwards. Therefore we plan to explore the impact of **ethnicity** using an alternative approach.

No.	Sensitivity analysis	Justification
19	We will consider repeating our analyses after restricting to individuals who **never smoked**.	Smoking may be recorded poorly, and smoking may act as a confounder for conflicting reasons (1. Atopy: individuals with eczema are more likely to have asthma and are therefore less likely to smoke; or 2. individuals with eczema may be more likely to smoke due to poor mental state as a result of skin disease). This analysis will therefore reduce **residual confounding** introduced as a result of smoking status.

7. Limitations

7.1 Misclassification of exposure

The diagnosis of eczema can be challenging. We propose to examine the effect of clinically diagnosed eczema, which will be different from the majority of other studies that identified eczema patients using self-reported questionnaires. We therefore may not able to capture all eczema cases, especially those with mild eczema who receive self-care in the family and community. We will use a validated algorithm to identify eczema diagnoses.[13] Ultimately, however, we are limited by the acumen of the clinician recording the diagnosis. As the majority of eczema diagnoses are managed in primary care, using a GP record of eczema is reasonable. We will undertake sensitivity analyses to determine the effect of altering our diagnostic definition for eczema (see **Section 6.3.1**).

7.2 Type 1 Error

Given the large number of planned analyses and sub-analyses, there may be an increased risk of type 1 error due to multiple testing; we plan to use 99% confidence intervals to reduce the risk of type 1 error.

7.3 Residual confounding

CPRD contains limited information on potential confounding factors, such as sleep deprivation or perceived levels of stress. The absence of these measures could lead to residual confounding.

7.4 Confounding by indication

In this study, severity is defined based on treatments received, as these data are available, therefore although we will determine if effect modification by the specific therapies (systemic immunosuppressive treatments, specifically, oral corticosteroids, cyclosporine or azathioprine) is present, we will not be able to completely disentangle the effects of therapy and severity. We believe that this issue is not a major problem as the majority of individuals with eczema in the UK (97%) are managed in primary care and hence only a very small proportion of individuals will ever have received systemic treatment for their eczema.

8. References

1 Deckert S, Kopkow C, Schmitt J. Nonallergic comorbidities of atopic eczema: An overview of systematic reviews. *Allergy Eur J Allergy Clin Immunol* 2014; **69**: 37–45.

2 Wedgeworth E, Powell AM, Flohr C. Eczema and cancer risk: A critical appraisal and review of the literature. *Br J Dermatol* 2011; **165**: 457–62.

3 Burki TK han. Association between allergies and reduced risk of glioma. *Lancet Oncol* 2016; **17**: e94.

4 Linos E, Raine T, Alonso A, Michaud D. Atopy and risk of brain tumors: A meta-analysis. *J Natl Cancer Inst* 2007; **99**: 1544–50.

5 Wang M, Chen C, Qu J, *et al.* Inverse association between eczema and meningioma: A meta-analysis. *Cancer Causes Control* 2011; **22**: 1355–63.

6 Legendre L, Barnetche T, Mazereeuw-Hautier J, Meyer N, Murrell D, Paul C. Risk of lymphoma in patients with atopic dermatitis and the role of topical treatment: A systematic review and meta-analysis. *J Am Acad Dermatol* 2015; **72**: 992–1002.

7 Linabery AM, Jurek AM, Duval S, Ross JA. The association between atopy and childhood/adolescent leukemia: A meta-analysis. *Am J Epidemiol* 2010; **171**: 749–64.

8 Margolis DJ, Abuabara K, Hoffstad OJ, Wan J, Raimondo D, Bilker WB. Association between malignancy and topical use of pimecrolimus. *JAMA Dermatology* 2015; **151**: 594–9.

9 Cipolat S, Hoste E, Natsuga K, Quist SR, Watt FM. Epidermal barrier defects link atopic dermatitis with altered skin cancer susceptibility. *Elife* 2014; **2014**: 1–21.

10 Margolis DJ, Hoffstad O, Bilker W. Lack of association between exposure to topical calcineurin inhibitors and skin cancer in adults. *Dermatology* 2007; **214**: 289–95.

11 Jensen AO, Svaerke C, Körmendiné Farkas D, Olesen AB, Kragballe K, Sørensen HT. Atopic Dermatitis and Risk of Skin Cancer. *Am J Clin Dermatol* 2012; **13**: 29–36.

12 Cheng J, Zens MS, Duell E, Perry AE, Chapman MS, Karagas MR. History of allergy and atopic dermatitis in relation to squamous cell and basal cell carcinoma of the skin. *Cancer Epidemiol Biomarkers Prev* 2015; **24**: 749–54.

13 Abuabara K, Magyari AM, Hoffstad O, *et al.* Development and Validation of an Algorithm to Accurately Identify Atopic Eczema Patients in Primary Care Electronic Health Records from the UK. *J Invest Dermatol* 2017; **137**: 1655–62.

14 Gelfand JM, Troxel AB, Lewis JD, *et al.* The Risk of Mortality in Patients With Psoriasis. *Arch Dermatol* 2007; **143**: 1493–9.

15 Bhaskaran K, Douglas I, Forbes H, Dos-Santos-Silva I, Leon DA, Smeeth L. Body-mass index and risk of 22 specific cancers: a population-based cohort study of 5·24 million UK adults. *Lancet* 2014; **6736**: 60892–8.

16 Boggon R, van Staa T-P, Chapman M, Gallagher AM, Hammad TA, Richards MA. Cancer recording and mortality in the General Practice Research Database and linked cancer registries. *Pharmacoepidemiol Drug Saf* 2013; **22**: 168–75.

17 Mathur R, Bhaskaran K, Chaturvedi N, *et al.* Completeness and usability of ethnicity data in UK-based primary care and hospital databases. *J Public Health (Oxf)* 2013; **36**: 684–92.

18 Robinson DE, Dennison EM, Cooper C, van Staa TP, Dixon WG. A review of the methods used to define glucocorticoid exposure and risk attribution when investigating the risk of fracture in a rheumatoid arthritis population. *Bone* 2016; **90**: 107–15.

9. Appendix

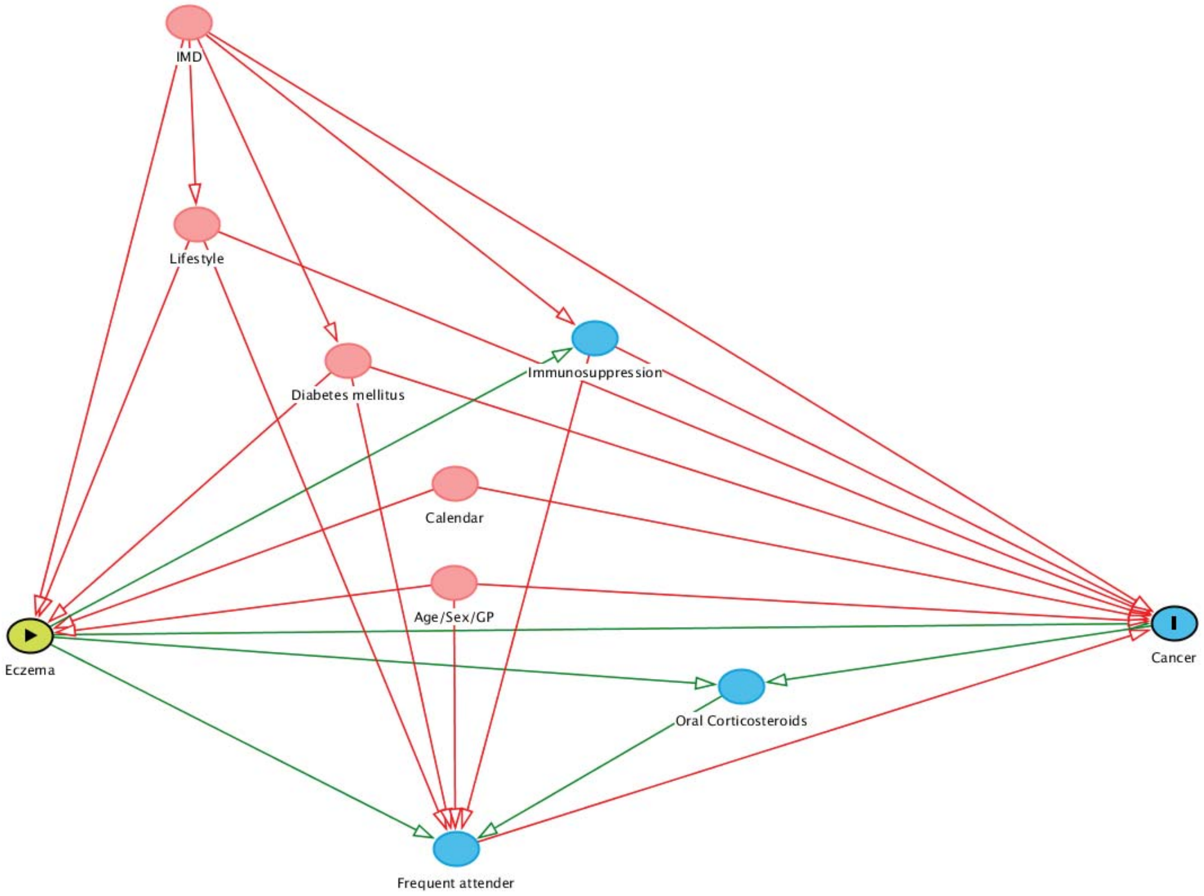

Figure 1. Directed acyclic graph illustrating implicitly assumed causal structure underlying our adjusted models.
