# Supplementary material for: Association Between Atopic Eczema and Cancer in England and Denmark
Source: JAMA Dermatol. 2020 Jun 24;156(10):1086–97. doi: 10.1001/jamadermatol.2020.1948 (PMC7315391; doi:10.1001/jamadermatol.2020.1948)
Supplement: Supplement 2. — Danish Trial Protocol [file jamadermatol-e201948-s002.pdf]

Study title: The association between atopic eczema and cancer: a cohort study

Objectives, Specific Aims and Rationale

- Primary aim: To examine if atopic eczema is associated with increased risk of all cancers and a number of specific cancers.
- Secondary aim: To examine if the risk depends on severity and activity of eczema, age, sex, coexisting asthma and systemic immunosuppressive treatments.

Of note, the study will also be conducted by colleagues in the United Kingdom in parallel.

Study Design: Nationwide matched cohort study.

Data sources

- The Danish National Patient Registry (inpatients since 1977, outpatient/emergency rooms since 1995)
- The Danish Cancer Registry (since 1943)
- The Civil Registration System (since 1968)
- The Danish National Health Service Registry (since 1990)
- The Danish National Prescription Registry (since 1995)
- Socioeconomic data (educational data since 1981, income and partnership data since 1980)

Study Population

The study will include an exposed cohort of persons with eczema and a comparison cohort including matched persons from the general population. The Danish Health Data Authority has already sampled the eczema exposed and unexposed cohorts described below.

Note: A sibling cohort has also been sampled by the Danish Health Data Authority, but is not included in this analysis plan, as we do not expect to include it in the current study.

Eczema exposed

- Persons with a diagnosis of atopic eczema (ICD-8: 691; ICD-10: DL20) in the Danish National Patient Registry between 1 Jan 1977 and 10 February 2018.
- We include all primary and secondary diagnoses for eczema from admissions, hospital outpatient clinics and emergency rooms in the Danish National Patient Registry, including ongoing contacts (that is, persons who at the end of study period are in active follow-up in a hospital outpatient clinic).
- Index date = The earliest hospital record for atopic eczema (date of admission or first outpatient appointment).
- Only persons who are born in Denmark and living in Denmark on the index date (i.e. date of first record for eczema).

For the current study, we will further restrict the study population as follows:

- We will exclude eczema patients (together with their matched comparators) identified by an ICD-8 diagnosis recorded together with an additional ("modification") code indicating that it potentially an uncertain/unverified/working diagnosis (c_diagmod=1–7).
- We will exclude patients (together with their matched comparators) with inconsistencies in registration of status and residence in the Civil Registration System (recorded as alive but not living in Denmark but without an emigration date), as we assume that these patients are not living in Denmark and thus not eligible.
- To ensure that we have at least 1 year of registration history from included registries (except the prescription registry), we will only include patients (together with their matched comparators) with index date on 1 January 1982 or later. In the subanalysis on severity and in a sensitivity analysis, we restrict to those diagnosed on 1 January 1996 or later in order to ensure one year of registration history in all databases.
- To ensure at least 1 year of follow-up after start of eligible follow-up (available until 30 June 2017), we will include eczema patients until 30 June 2016.
- We will exclude eczema patients (together with their matched comparators) with a record for any cancer (except non-melanoma skin cancer) before or on index date (see Table 2). That is, we will not only exclude those with history of the cancer outcome of interest. However, in analyses of non-melanoma skin cancer specifically, we will exclude those with previous history of this cancer as well.

Unexposed cohort

- Up to 10 persons matched to each eczema patient by sex and birth year.
- Comparators have to: (1) be born in Denmark, (2) be alive and living in Denmark on the on index date of their matched eczema-exposed individual (i.e. first hospital eczema diagnosis), and (3) have no previous diagnosis of eczema.
- Index date = The same index date as their matched eczema-exposed individual

As with eczema patients, we will additionally apply the following restrictions:

- We will exclude matched comparators with inconsistencies in registration of status and residence in the Civil Registration System (recorded as alive but not living in Denmark but without an emigration date), as we assume that these patients are not living in Denmark and thus not eligible.
- We will exclude comparators with any cancer (except non-melanoma skin cancer) before or on index date (see Table 2). That is, we will not only exclude those with history of the cancer outcome of interest. However, in analyses of non-melanoma skin cancer specifically, we will exclude those with previous history of this cancer as well.

Exposure subgroups

For the secondary aim, we will identify severe and active eczema. The analyses will be perfomed if feasible in terms of sample size.

We will categorize severity as moderate or severe. Severe eczema will be defined as (1) either phototherapy or systemic treatment for eczema (cyclosporine, azathioprine, mycophenolate, methotrexate, dupilumab), identified in Patient Registry (procedure codes: “BNGA1” “BNGA2” “BNGA3” “BNGA4” “BOHJ18B8” “BWHB83” “BWHA115” “BOHJ20” “BOHJ22”) or Prescription Registry (ATC codes: “L04AX01” “L01BA01” “L04AX03” “L04AD01” “L04AA06” “D11AH05”) or (2) admission to hospital with eczema as the primary reason for admission (i.e. where eczema is recorded in the primary diagnostic position for an in-hospital contact). Severity will be included as a time-updated variable. Thus, a patient with eczema will be classified as having moderate eczema from index date (unless they develop severe eczema). When (and if) a person fulfills the definition for severe eczema, we will classify them as having severe eczema from the

point at which they meet the definition for ‘severe eczema’. Once a person fulfills the criteria for severe disease, he/she will remain in that category for the rest of follow-up. At any given point during follow-up, atopic eczema patients will therefore belong to one of two severity categories: moderate or severe.

We will classify activity of eczema based on consultation and treatment patterns within the first year after index date, as illustrated in the Figure below. A person will be considered to have active eczema that lasts 3 months from either 1) a consultation to a private-practicing dermatologist (any consultations coded with specialty ‘04’ in The Danish National Health Service Registry), 2) a primary care prescription for eczema therapies (Prescription Registry ATC codes: “D07” “D11AH01” “D11AH02” “L04AX01” “L04AX03” “L04AD01” “L04AA06” ”H02AB” [excluding injections] or Patient Registry procedure codes: “BNGA1” “BNGA2” “BNGA3” “BNGA4” “BOHJ18B8” “BWHB83” “BWHA115” “BOHJ20” “BOHJ22”) or 3) a hospital record for eczema. Each time a person fulfills one of these criteria, the active period will be extended by 3 months from that date. We will then compute the percentage of time with active eczema in the first 12 months after index date as the number of months with active eczema divided by 12 months. We will then classify persons as: 0=unexposed; 1=those who never had active atopic eczema in the first year; 2=those who had active atopic eczema for less than 50% of the first year; 3=those who had active atopic eczema for at least 50% of the first year. In order to classify activity, follow-up will start at 12 months after index date in activity analyses. Note that periods of disease activity will be identified irrespective of the classification for disease severity.

Figure. Illustration of active eczema disease

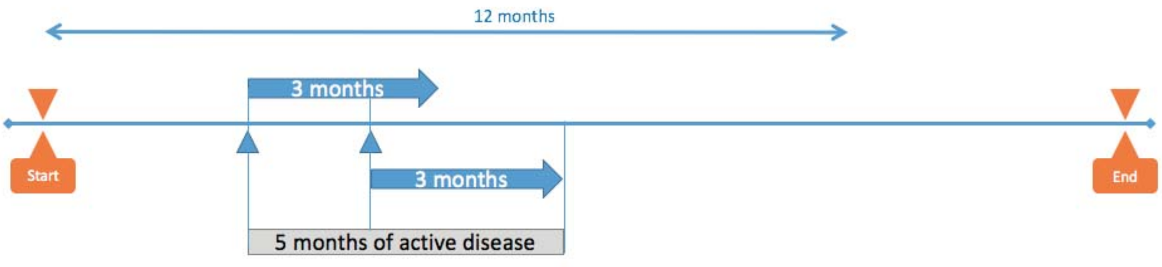

$$\%\ active\ eczema = \frac{number\ of\ months\ with\ active\ eczema\ in\ first\ 12\ months\ of\ follow\ up}{12\ months}$$

Outcome definitions

- Cancer diagnosis recorded in the Danish Cancer Registry

- If possible (depending on power), we will present results for subtypes of hematological and brain cancers, rather than overall types. As a rule of thumb, we will not publish results based on analyses of outcomes with less than 20 events.

Table 2. Cancer outcomes

	ICD-7	ICD-10	Notes
**Any cancer (excluding non-melanoma skin cancer)**	”140” ”141” ”142” ”143” ”144” ”145” ”146” ”147” ”148” ”149” ”150” ”151” ”152” ”153” ”154” ”155” ”156” ”157” ”158” ”159” ”160” ”161” ”162” ”163” ”164” ”165” ”166” ”167” ”168” ”169” ”170” ”171” ”172” ”173” ”174” ”175” ”176” ”177” ”178” ”179” ”180” ”181” ”182” ”183” ”184” ”185” ”186” ”187” ”188” ”189” ”190” ”192” ”193” ”194” ”195” ”196” ”197” ”198” ”199” ”200” ”201” ”202” ”203” ”27559” ”204” ”205” ”206” ”207”	”DC00” ”DC01” ”DC02” ”DC03” ”DC04” ”DC05” ”DC06” ”DC07” ”DC08” ”DC09” ”DC10” ”DC11” ”DC12” ”DC13” ”DC14” ”DC15” ”DC16” ”DC17” ”DC18” ”DC19” ”DC20” ”DC21” ”DC22” ”DC23” ”DC24” ”DC25” ”DC26” ”DC27” ”DC28” ”DC29” ”DC30” ”DC31” ”DC32” ”DC33” ”DC34” ”DC35” ”DC36” ”DC37” ”DC38” ”DC39” ”DC40” ”DC41” ”DC42” ”DC43” ”DC45” ”DC46” ”DC47” ”DC48” ”DC49” ”DC50” ”DC51” ”DC52” ”DC53” ”DC54” ”DC55” ”DC56” ”DC57” ”DC58” ”DC59” ”DC60” ”DC61” ”DC62” ”DC63” ”DC64” ”DC65” ”DC66” ”DC67” ”DC68” ”DC69” ”DC70” ”DC71” ”DC72” ”DC73 ”DC74” ”DC75” ”DC76” ”DC77” ”DC78”	For exclusions of persons with previous cancer, we will exclude those with any cancer code except except non-melanoma skin cancer (have already been excluded from the list). However, in analyses of melanoma and keratinocyte skin cancer specifically, we will exclude those with previous history of non-melanoma skin cancer (ICD-7: 191; ICD-10: C44) as well.<br><br>Furthermore, in order to explore the impact of potential ascertainment bias of skin cancers, we will conduct a sensitivity analysis by repeating the all-cancer analysis after excluding all skin cancer (non-melanoma and melanoma skin cancer) from the outcome definition.

		”DC79” ”DC80” ”DC81” ”DC82” ”DC83” ”DC84” ”DC85” ”DC88” ”DC90” ”DC96” ”DC91” ”DC92” ”DC93” ”DC94” ”DC95” except codes for cutaneous lymphoma (”C826” ”C840” ”C841” ”C848” ”C863” ”C866” ”C884B”)	
**Lung**		“C34”	
**Breast**		“C50”	
**Prostate**		“C61”	
**Pancreatic**		“C25”	
**Skin**			
Melanoma	”190”	“C43”	
Keratinocyte cancers	”191”	“C44” if any of the following morphology codes: ”80903” ”80913” ”80923” ”80933” ”80973” ”81233” ”80513” ”80523” ”80703” ”80713” ”80723” ”80733” ”80743” ”80753” ”80763” ”80943” ”80953”	Morphology codes will be used to identify BCC and SCC specifically (to exclude rarer NMSCs).
**Haematological**		All codes below	We will also compute estimates for overall hematological cancer and present if there is not power for subtypes
Lymphoma		“C81” “C82” “C83” “C84” “C85” “C86” “C88” except codes for cutaneous lymphoma (”C826” ”C840” ”C841” ”C848” ”C863” ”C866” ”C884B”)	We will make no distinction between Hodgkin’s and non-Hodgkin’s lymphoma (but will stratify if power allows)
Multiple myeloma		“C90”	
Leukaemia		“C91” “C92” “C93” “C94” “C95”	

**CNS**		All codes below	We will also compute estimates for overall CNS cancer and present if there is not power for subtypes
Meningioma		"C70"	
Cancer of the brain, hypophysis, corpus pineale and ductus craniopharyngealis		"C71"	If power is sufficient, we will look at glioma separately.
Spinal cord, cranial nerve or other CNS tumours		"C72"	

CNS: central nervous system
Note: All subcodes are included unless otherwise stated

Follow-up

- Follow-up start: index date.
- Follow-up end: earliest of emigration, death, end of study (30 June 2017) or first-ever cancer diagnosis (when examining specific cancers, patients are thus censored if diagnosed with another individual cancer).
- If a person from the comparison cohort is diagnosed with eczema during follow-up, that person will be censored at time of diagnosis. The person will then contribute to the eczema cohort together with his/her own comparators (this has been accounted for in the sampling done by the Danish Health Data Authority)

Covariables

We will include various variables, shown in table 3. With the exception of socioeconomic variables, variables are time-updated. Thus, people will contribute person-time in the unexposed category for a given variable until they fulfill the definition for the condition/treatment, after which they will contribute in the exposed category for the remaining follow-up.

Table 3. Covariables

	ICD-8	ICD-10	Other codes in patient registry	ATC	Notes

Age				<18<br>18–44<br>45–64<br>≥65 years
Sex				Men<br>Women
Calendar period				1982–1999<br>2000–2004<br>2005–2009<br>2010–2015
Various lifestyle-related diseases, as proxy for unhealthy lifestyle				
Chronic obstructive pulmonary disease	“491”<br>“492”	“DJ41” “DJ42”<br>“DJ43” “DJ44”		
Hyperlipidemia or treatment of hyperlipidemia	“27200”	“DE780”	“C10”	
Hypertension or antihypertensive treatment	”400”<br>”401”<br>”402”<br>”403”<br>”404”	”DI10” ”DI11”<br>”DI12” ”DI13”<br>”DI14” ”DI15”<br>”DI674”	“C02”<br>“C03”<br>“C07”<br>“C08”<br>“C09”	
Alcohol-related conditions	“291”<br>“303”<br>“57109”<br>“57110”<br>“57710”<br>“979”<br>“980”	”DF10” ”DG312”<br>”DG621”<br>”DG721” ”DI426”<br>”DK292”<br>”DK700”<br>”DK703”<br>“DK860”<br>“DR780”<br>“DT510”<br>“DT519”<br>“DZ721”	“N07BB01”	
Ischemic heart disease	”410”<br>”411”<br>”412”<br>”413”<br>”414”	”DI20” ”DI21”<br>”DI22” ”DI23”<br>”DI24” ”DI25”<br>”DT823D”<br>”DT823E”		“30009” “30019”<br>“30029” “30039”<br>“30049” “30059”<br>“30069” “30079”<br>“30089” “30099”<br>“30109” “30119”

					“30120” “30129” “30139” “30149” “30159” “30169” “30179” “30189” “30199” “KFNA” “KFNB” “KFNC” “KFND” “KFNE” “KFNH20” “KFNG” “KFNF”
Hospital-diagnosed obesity	“277”	“DE65” “DE66”			
Type II diabetes	“250”	“DE11” “DO241”		Oral antidiabetic = “A10B” (excluding “A10BE01”)	Any type II ICD code or second prescription for oral antidiabetic
Socioeconomic status (education and social registries from Statistics Denmark)[a]					For use in a sensitivity analysis among those aged 30 years or older at index date.
Highest attained educational level (primary education, secondary education, or higher education)					• Short term education (7-10 years) if afsp1e code = 10<br>• Medium term education (11-12 years) if afsp1e code 20<= & <40<br>• Long term education (13+ years) if afsp1e code 40<= & <90

					• Missing if afsp1e has any other value
Partnership status (married/cohabitating vs. single)					Based on algorithm defined by Statistics Denmark. Those who have non-missing data on variables EFALLE (table BEF, during 1986-) and C_faelle_id (table FAIN, 1980-1985) will be recorded as in a partnership (=1) and remaining patients are considered single (=0)[b]
Gross personal income					Based on variable PERINDKIALT _13 (during 1987-) and PERINDKIALT (1980-1986). Categorized yearly (to account for inflation) based on quartiles.
Immunosuppression (immunosuppressive disorders and drugs, excluding oral corticosteroid use)	”07983” ”28401” ”28402” ”28408” ”28409”	”DB20” ”DB21” ”DB22” ”DB23” ”DB24” ”DF024” ”DZ21” ”DT860” “DZ948C”	Procedure codes: ”BOQE” “BOQF” “BOHJ”	“L01” “L04” “V02CA01” “V02CA02”	Exclude codes for myeloma, lymphoma and leukemia respectively from code list for the

	”75830”<br>“203”<br>”204”<br>”205”<br>”206”<br>”207”<br>”200”<br>”201”<br>”202”	“DZ948” (if not “DZ948A” “DZ948B” or “DZ948C” **and** if coded as a B-diagnosis or additional diagnosis together with one of the following A-diagnoses “DC770” “DC81” “DC82” “DC83” “DC84” “DC85” “DC86” “DC87” “DC88” “DC89” “DC90” “DC91” “DC92” “DC93” “DC94” “DC95” “DC96”, “DD45” “DD46” “DD47” “DD5” “DD6” “DD7” “DD80” “DD81” “DD82” “DD83” “DD84” “DD85” “DD87” “DD88” “DD89” “DT860” “DT860A” “DT888N”)<br><br>”DD611” ”DD612” ”DD613” ”DD618” ”DD619” ”DD81” ”DD820” ”DD821” ”DD821A” ”DD822” ”DD83” “DC90” ”DC91” ”DC92” ”DC93”	“BWG”<br>“BWHB”<br><br>Or<br><br>Any ATC code for “L01” “L04” “V02CA01” or “V02CA02” used as additional code in the Patient Registry	variable when these cancers are the outcome (ICD-8: “203” ”204” ”205” ”206” ”207” ”200” ”201” ”202”; ICD-10: “DC90” ”DC91” ”DC92” ”DC93” ”DC94” ”DC95” ”DC81” ”DC82” ”DC83” ”DC84” ”DC85” ”DC86” ”DC88” ”DC96”)

		”DC94” ”DC95” ”DC81” ”DC82” ”DC83” ”DC84” ”DC85” ”DC86” ”DC88” ”DC96”			
Use of oral corticosteroids				”H02AB” and DOSFORM “TAB” or “TABMOD”	Any prescription excluding injections.
Systemic treatment for eczema (cyclosporine, azathioprine, mycophenolate, methotrexate, dupilumab)			Procedure codes: “BOHJ18B8” “BWHB83” “BWHA115” “BOHJ20” “BOHJ22”	“L04AX01” “L01BA01” “L04AX03” “L04AD01” “L04AA06” “D11AH05”	
Asthma	“493”	“DJ45” “DJ46”			

Note: All subcodes are included unless otherwise stated; all types of contacts (inpatient, outpatient and emergency) and both primary and secondary diagnoses should be considered. Use admission/prescription/record date for all variables.
[a]Baseline information only
[b]Documentation is available here:
https://www.dst.dk/da/Statistik/dokumentation/Times/forskningsservice/efalle
https://www.dst.dk/da/Statistik/dokumentation/Times/cpr-oplysninger/c-faelle-id

Statistical analyses

1. Construct study population. Statistics Denmark has already sampled eczema patients and comparators. However, we need to apply the additional exclusions described above in the section on Study Population.

2. Compute summary statistics for covariables (at baseline) and accumulated person-time for exposed (eczema) patients and their comparators (Table 1).

Table 1. Selected characteristics of eczema patients and matched comparison cohort, Denmark		
	**Eczema patients**	**Comparison cohort**
	**No. (%)**	**No. (%)**
Total		
Age, years		
Range		
Median (IQR)		
Groups		
<18		
18–44		
45–64		
≥65		
Sex		
Women		
Men		
Setting for first eczema diagnosis		
Inpatient		–
Outpatient clinic		–
Emergency room		–
Calendar period of index date		
1982–1999		
2000–2004		
2005–2009		
2010–2016		
Lifestyle-related diseases		
Chronic obstructive pulmonary disease		
Hyperlipidemia or treatment of hyperlipidemia		
Hypertension or antihypertensive treatment		
Alcohol-related conditions		
Ischemic heart disease		
Hospital-diagnosed obesity		
Diabetes type II		
Immunosuppression		
Oral corticosteroids		
Asthma		

Educational level*		
Short		
Medium		
Long		
Missing		
Partnership status (married/cohabitating vs. single)*		
Single		
Married/cohabitating		
Gross personal income*		
Low		
Intermediate		
High		
Very high		
Follow-up (years)		
Total		
Median (IQR)		
Abbreviations: IQR = interquartile range		

*Educational level, partnership status and gross personal income are presented for those 30 years or older at index date only. To enable interpretation of the categories for gross personal income, we will check the category boundaries for the first and last year of the inclusion period, as well as the mean for the boundaries over the entire study period.

3. With time since index date as underlying time scale, we will use stratified Cox regression (conditioning on matched set) to compute unadjusted hazard ratios (**99**% confidence intervals) to determine associations between eczema and each outcome (that is, comparing eczema patients and the comparison cohort). By stratifying on matched set, the model accounts for birth year, sex and calendar period. We will then fit a "mediation model" additionally adjusting for time-varying lifestyle-related diseases (chronic obstructive pulmonary disease, hyperlipidemia, hypertension, alcohol-related conditions, ischemic heart disease, hospital-diagnosed obesity and type II diabetes). If sufficient power, we will adjust for the lifestyle-related diseases as individual variables.

Table 2. Number of events, accumulated person-time, and HRs for cancer associated with eczema, Denmark

					Hazard ratio and 99% confidence interval[1]	
	N	events	P-Y at risk	Rate per 1,000	Unadjusted	Mediation model
Any cancer						
Unexposed						
Exposed						
Lung cancer						
Unexposed						
Exposed						
Breast cancer						
Unexposed						
Exposed						
Prostate cancer						
Unexposed						
Exposed						
Melanoma skin cancer						
Unexposed						
Exposed						
Keratinocyte skin cancer						
Unexposed						
Exposed						
Lymphoma						
Unexposed						
Exposed						
Multiple myeloma						
Unexposed						

Exposed
Leukaemia
Unexposed
Exposed
Meningioma
Unexposed
Exposed
Cancer of the brain, hypophysis, corpus pineale and ductus craniopharyngealis
Unexposed
Exposed
Spinal cord, cranial nerve and other CNS tumours
Unexposed
Exposed

[1]Estimated hazard ratios from Cox regression with time since index date as underlying timescale, stratified by matched set.
Unadjusted: No adjustment.
Mediation model: Adjusted additionally for time-varying lifestyle-related diseases.

4. For the atopic eczema cohort, we will make a plot of the cumulative incidence function by age for overall and specific cancers (lung, breast, prostate, lymphoma, leukemia, multiple myeloma, CNS cancers, melanoma and keratinocyte cancer) non-parametrically. This will be done for the unadjusted model.

5. We will assess the assumption of proportional hazards model in all the primary analysis models through Schöenfeld residual plots.

Subgroup analyses

Depending on power, subgroup analyses may only be performed for overall cancer.

6. We will explore whether the association between eczema and cancer differs by eczema severity or eczema disease activity by stratifying separately on disease severity (Table 4) and activity (Table 5). The severity and activity analyses will be restricted to those with index date on or after 1 January 1996 to ensure at least 1 year of prescription history. Furthermore, the follow-up will start at 12 months after index date in the activity analysis in order to allow categorization of activity.

Table 3. Number of events, accumulated person-time, and HRs for cancer associated with eczema severity, Denmark

					Hazard ratio and 99% confidence interval[1]	
	N	events	P-Y at risk	Rate per 1,000	Unadjusted	Mediation model
Any cancer						
Unexposed						
Exposed – moderate						
Exposed – severe						
Lung cancer						
Unexposed						
Exposed – moderate						
Exposed – severe						
Breast cancer						
Unexposed						
Exposed – moderate						
Exposed – severe						
Prostate cancer						
Unexposed						
Exposed – moderate						
Exposed – severe						
Pancreatic cancer						
Unexposed						
Exposed – moderate						
Exposed – severe						
Melanoma						

Unexposed						
Exposed – moderate						
Exposed – severe						
Keratinocyte cancer						
Unexposed						
Exposed – moderate						
Exposed – severe						
Lymphoma						
Unexposed						
Exposed – moderate						
Exposed – severe						
Multiple myeloma						
Unexposed						
Exposed – moderate						
Exposed – severe						
Leukaemia						
Unexposed						
Exposed – moderate						
Exposed – severe						
Meningioma						
Unexposed						
Exposed – moderate						
Exposed – severe						
Cancer of the brain, hypophysis, corpus pineale and ductus craniopharyngealis						
Unexposed						
Exposed – moderate						
Exposed – severe						
Spinal cord, cranial nerve or other CNS tumours						
Unexposed						
Exposed – moderate						
Exposed – severe						

[1]Estimated hazard ratios from Cox regression with time since index date as underlying timescale, stratified by matched set.
Unadjusted: No adjustment.
Mediation model: Adjusted additionally for time-varying lifestyle-related diseases.

Table 4. Number of events, accumulated person-time, and HRs for cancer associated with eczema activity, Denmark

					Hazard ratio and 99% confidence interval[1]	
	N	events	P-Y at risk	Rate per 1,000	Unadjusted	Mediation model
Any cancer						

- Unexposed
- Exposed – never active
- Exposed – <50% active
- Exposed – ≥50% active

Lung cancer
- Unexposed
- Exposed – never active
- Exposed – <50% active
- Exposed – ≥50% active

Breast cancer
- Unexposed
- Exposed – never active
- Exposed – <50% active
- Exposed – ≥50% active

Prostate cancer
- Unexposed
- Exposed – never active
- Exposed – <50% active
- Exposed – ≥50% active

Pancreatic cancer
- Unexposed
- Exposed – never active
- Exposed – <50% active
- Exposed – ≥50% active

Melanoma
- Unexposed
- Exposed – never active
- Exposed – <50% active
- Exposed – ≥50% active

Keratinocyte cancer
- Unexposed
- Exposed – never active
- Exposed – <50% active
- Exposed – ≥50% active

Lymphoma
- Unexposed
- Exposed – never active
- Exposed – <50% active
- Exposed – ≥50% active

Multiple myeloma
- Unexposed
- Exposed – never active
- Exposed – <50% active
- Exposed – ≥50% active

Leukaemia
- Unexposed
- Exposed – never active
- Exposed – <50% active

Exposed – ≥50% active						
Meningioma						
Unexposed						
Exposed – never active						
Exposed – <50% active						
Exposed – ≥50% active						
Cancer of the brain, hypophysis, corpus pineale and ductus craniopharyngealis						
Unexposed						
Exposed – never active						
Exposed – <50% active						
Exposed – ≥50% active						
Spinal cord, cranial nerve or other CNS tumours						
Unexposed						
Exposed – never active						
Exposed – <50% active						
Exposed – ≥50% active						

[1]Estimated hazard ratios from Cox regression with time since index date as underlying timescale, stratified by matched set.
Unadjusted: No adjustment.
Mediation model: Adjusted additionally for time-varying lifestyle-related diseases.

7. As a subgroup analysis, we will stratify the results by variables listed below:

- Age (Table 5)
- Sex (Table 6)
- Asthma (Table 7)

Table 5. Number of events, accumulated person-time, and HRs for cancer associated with eczema, Denmark, by age

	N	events	P-Y at risk	Rate per 1,000	Hazard ratio and 99% confidence interval[1]	
					Unadjusted	Mediation model
Any cancer						
Age <18 years						
Unexposed						
Exposed						
Age 18–44 years						
Unexposed						

Exposed
Age 45–64 years
Unexposed
Exposed
Age >64 years
Unexposed
Exposed
Lung cancer
Age <18 years
Unexposed
Exposed
Age 18–44 years
Unexposed
Exposed
Age 45–64 years
Unexposed
Exposed
Age >64 years
Unexposed
Exposed
Breast cancer
Age <18 years
Unexposed
Exposed
Age 18–44 years
Unexposed
Exposed
Age 45–64 years
Unexposed
Exposed
Age >64 years
Unexposed
Exposed
Prostate cancer
Age <18 years
Unexposed
Exposed
Age 18–44 years
Unexposed
Exposed
Age 45–64 years
Unexposed
Exposed
Age >64 years
Unexposed
Exposed
Pancreatic cancer
Age <18 years

Unexposed
Exposed
Age 18–44 years
Unexposed
Exposed
Age 45–64 years
Unexposed
Exposed
Age >64 years
Unexposed
Exposed
Melanoma
Age <18 years
Unexposed
Exposed
Age 18–44 years
Unexposed
Exposed
Age 45–64 years
Unexposed
Exposed
Age >64 years
Unexposed
Exposed
Keratinocyte cancer
Age <18 years
Unexposed
Exposed
Age 18–44 years
Unexposed
Exposed
Age 45–64 years
Unexposed
Exposed
Age >64 years
Unexposed
Exposed
Lymphoma
Age <18 years
Unexposed
Exposed
Age 18–44 years
Unexposed
Exposed
Age 45–64 years
Unexposed
Exposed
Age >64 years

Unexposed
Exposed
Multiple myeloma
Age <18 years
Unexposed
Exposed
Age 18–44 years
Unexposed
Exposed
Age 45–64 years
Unexposed
Exposed
Age >64 years
Unexposed
Exposed
Leukaemia
Age <18 years
Unexposed
Exposed
Age 18–44 years
Unexposed
Exposed
Age 45–64 years
Unexposed
Exposed
Age >64 years
Unexposed
Exposed
Meningioma
Age <18 years
Unexposed
Exposed
Age 18–44 years
Unexposed
Exposed
Age 45–64 years
Unexposed
Exposed
Age >64 years
Unexposed
Exposed
Cancer of the brain, hypophysis, corpus pineale and ductus craniopharyngealis
Age <18 years
Unexposed
Exposed
Age 18–44 years

Unexposed
Exposed
Age 45–64 years
Unexposed
Exposed
Age >64 years
Unexposed
Exposed
Spinal cord, cranial nerve or other CNS tumours
Age <18 years
Unexposed
Exposed
Age 18–44 years
Unexposed
Exposed
Age 45–64 years
Unexposed
Exposed
Age >64 years
Unexposed
Exposed

[1]Estimated hazard ratios from Cox regression with time since index date as underlying timescale, stratified by matched set.
Unadjusted: No adjustment.
Mediation model: Adjusted additionally for time-varying lifestyle-related diseases.

Table 6. Number of events, accumulated person-time, and HRs for cancer associated with eczema, Denmark, by sex

					Hazard ratio and 99% confidence interval[1]	
	N	events	P-Y at risk	Rate per 1,000	Unadjusted	Mediation model
Any cancer						
Female						
Unexposed						
Exposed						
Male						
Unexposed						
Exposed						
Lung cancer						
Female						
Unexposed						
Exposed						
Male						

Unexposed
Exposed
Breast cancer
Female
Unexposed
Exposed
Male
Unexposed
Exposed
Prostate cancer
Female
Unexposed
Exposed
Male
Unexposed
Exposed
Pancreatic cancer
Female
Unexposed
Exposed
Male
Unexposed
Exposed
Melanoma
Female
Unexposed
Exposed
Male
Unexposed
Exposed
Keratinocyte cancer
Female
Unexposed
Exposed
Male
Unexposed
Exposed
Lymphoma
Female
Unexposed
Exposed
Male
Unexposed
Exposed
Multiple myeloma
Female
Unexposed
Exposed

Male						
Unexposed						
Exposed						
Leukaemia						
Female						
Unexposed						
Exposed						
Male						
Unexposed						
Exposed						
Meningioma						
Female						
Unexposed						
Exposed						
Male						
Unexposed						
Exposed						
Cancer of the brain, hypophysis, corpus pineale and ductus craniopharyngealis						
Female						
Unexposed						
Exposed						
Male						
Unexposed						
Exposed						
Spinal cord, cranial nerve or other CNS tumours						
Female						
Unexposed						
Exposed						
Male						
Unexposed						
Exposed						

[1]Estimated hazard ratios from Cox regression with time since index date as underlying timescale, stratified by matched set.
Unadjusted: No adjustment.
Mediation model: Adjusted additionally for time-varying lifestyle-related diseases.

Table 7. Number of events, accumulated person-time, and HRs for cancer associated with eczema, Denmark, by presence of asthma

					Hazard ratio and 99% confidence interval[1]	
	N	events	P-Y at risk	Rate per 1,000	Unadjusted	Mediation model

Any cancer
No asthma
Unexposed
Exposed
Asthma
Unexposed
Exposed
Lung cancer
No asthma
Unexposed
Exposed
Asthma
Unexposed
Exposed
Breast cancer
No asthma
Unexposed
Exposed
Asthma
Unexposed
Exposed
Prostate cancer
No asthma
Unexposed
Exposed
Asthma
Unexposed
Exposed
Pancreatic cancer
No asthma
Unexposed
Exposed
Asthma
Unexposed
Exposed
Melanoma
No asthma
Unexposed
Exposed
Asthma
Unexposed
Exposed
Keratinocyte cancer
No asthma
Unexposed
Exposed
Asthma
Unexposed

Exposed
Lymphoma
No asthma
Unexposed
Exposed
Asthma
Unexposed
Exposed
Multiple myeloma
No asthma
Unexposed
Exposed
Asthma
Unexposed
Exposed
Leukaemia
No asthma
Unexposed
Exposed
Asthma
Unexposed
Exposed
Meningioma
No asthma
Unexposed
Exposed
Asthma
Unexposed
Exposed
Cancer of the brain, hypophysis, corpus pineale and ductus craniopharyngealis
No asthma
Unexposed
Exposed
Asthma
Unexposed
Exposed
Spinal cord, cranial nerve or other CNS tumours
No asthma
Unexposed
Exposed
Asthma
Unexposed
Exposed

[1]Estimated hazard ratios from Cox regression with time since index date as underlying timescale, stratified by matched set.

Unadjusted: No adjustment.
Mediation model: Adjusted additionally for time-varying lifestyle-related diseases.

Sensitivity analyses

8. We will examine if the association is reduced by adjustment for time-varying systemic treatment for eczema. The study is likely insufficiently powered to examine for effect measure modification by immunosuppressive treatments.

Table 8. Number of events, accumulated person-time, and HRs for cancer associated with eczema, Denmark. Adjusting additionally for systemic treatment for eczema.

					Hazard ratio and 99% confidence interval[1]	
	N	events	P-Y at risk	Rate per 1,000	Unadjusted	Mediation model
Any cancer						
Unexposed						
Exposed						
Lung cancer						
Unexposed						
Exposed						
Breast cancer						
Unexposed						
Exposed						
Prostate cancer						
Unexposed						
Exposed						
Melanoma skin cancer						
Unexposed						
Exposed						
Keratinocyte skin cancer						
Unexposed						
Exposed						
Lymphoma						
Unexposed						
Exposed						
Multiple myeloma						
Unexposed						
Exposed						
Leukemia						
Unexposed						
Exposed						
Meningioma						
Unexposed						
Exposed						

Cancer of the brain, hypophysis, corpus pineale and ductus craniopharyngealis						
Unexposed						
Exposed						
Spinal cord, cranial nerve and other CNS tumours						
Unexposed						
Exposed						

[1]Estimated hazard ratios from Cox regression with time since index date as underlying timescale, stratified by matched set. Adjusted additionally for **systemic treatment for eczema.**
Unadjusted: No adjustment.
Mediation model: Adjusted additionally for time-varying lifestyle-related diseases and **systemic treatment for eczema**.

9. We will repeat the main analysis after starting follow-up at 3 years after index date to limit potential reverse causality. If we see a large change at 3 years, we will also repeat the main analysis by changing start of follow-up to 5 years after index date.

Table 9. Number of events, accumulated person-time, and HRs for cancer associated with eczema, Denmark. Starting follow-up at 3 years after index date.

					Hazard ratio and 99% confidence interval[1]	
	N	events	P-Y at risk	Rate per 1,000	Unadjusted	Mediation model
Any cancer						
Unexposed						
Exposed						
Lung cancer						
Unexposed						
Exposed						
Breast cancer						
Unexposed						
Exposed						
Prostate cancer						
Unexposed						
Exposed						
Melanoma skin cancer						
Unexposed						
Exposed						
Keratinocyte skin cancer						
Unexposed						
Exposed						
Lymphoma						
Unexposed						
Exposed						

Multiple myeloma						
Unexposed						
Exposed						
Leukemia						
Unexposed						
Exposed						
Meningioma						
Unexposed						
Exposed						
Cancer of the brain, hypophysis, corpus pineale and ductus craniopharyngealis						
Unexposed						
Exposed						
Spinal cord, cranial nerve and other CNS tumours						
Unexposed						
Exposed						

[1]Estimated hazard ratios from Cox regression with time since index date as underlying timescale, stratified by matched set.
Unadjusted: No adjustment.
Mediation model: Adjusted additionally for time-varying lifestyle-related diseases.

10. We will repeat the main analysis additionally adjusting for immunosuppression (immunosuppressive disorders or drugs).

Table 10. Number of events, accumulated person-time, and HRs for cancer associated with eczema, Denmark. Adjusting additionally for immunosuppression.

					Hazard ratio and 99% confidence interval[1]	
	N	events	P-Y at risk	Rate per 1,000	'Unadjusted'	Mediation model
Any cancer						
Unexposed						
Exposed						
Lung cancer						
Unexposed						
Exposed						
Breast cancer						
Unexposed						
Exposed						
Prostate cancer						
Unexposed						
Exposed						

Melanoma skin cancer						
Unexposed						
Exposed						
Keratinocyte skin cancer						
Unexposed						
Exposed						
Lymphoma						
Unexposed						
Exposed						
Multiple myeloma						
Unexposed						
Exposed						
Leukemia						
Unexposed						
Exposed						
Meningioma						
Unexposed						
Exposed						
Cancer of the brain, hypophysis, corpus pineale and ductus craniopharyngealis						
Unexposed						
Exposed						
Spinal cord, cranial nerve and other CNS tumours						
Unexposed						
Exposed						

[1]Estimated hazard ratios from Cox regression with time since index date as underlying timescale, stratified by matched set.
Unadjusted: Adjusted additionally for **immunosuppression.**
Mediation model: Adjusted additionally for time-varying lifestyle-related diseases **and immunosuppression**.

11. We will repeat the main analysis additionally adjusting for use of **oral corticosteroids**. We will define use as a time-updated never/ever variable. Thus, individuals classified as exposed if they ever have a prescription for an oral corticosteroid, with status changing at the first prescription for an oral corticosteroid.

Table 11. Number of events, accumulated person-time, and HRs for cancer associated with eczema, Denmark. Adjusting additionally for oral glucocorticoids.

	N	events	P-Y at risk	Rate per 1,000	Hazard ratio and 99% confidence interval[1]	
					'Unadjusted'	Mediation model

Any cancer	
Unexposed	
Exposed	
Lung cancer	
Unexposed	
Exposed	
Breast cancer	
Unexposed	
Exposed	
Prostate cancer	
Unexposed	
Exposed	
Melanoma skin cancer	
Unexposed	
Exposed	
Keratinocyte skin cancer	
Unexposed	
Exposed	
Lymphoma	
Unexposed	
Exposed	
Multiple myeloma	
Unexposed	
Exposed	
Leukemia	
Unexposed	
Exposed	
Meningioma	
Unexposed	
Exposed	
Cancer of the brain, hypophysis, corpus pineale and ductus craniopharyngealis	
Unexposed	
Exposed	
Spinal cord, cranial nerve and other CNS tumours	
Unexposed	
Exposed	

[1]Estimated hazard ratios from Cox regression with time since index date as underlying timescale, stratified by matched set.
Unadjusted: Adjusted additionally for **oral glucocortoids.**
Mediation model: Adjusted additionally for time-varying lifestyle-related diseases **and oral glucocorticoids**.

12. We will repeat the main analysis after not censoring unexposed persons who are diagnosed with eczema. Thus, after diagnosis of eczema, unexposed persons will contribute to both unexposed and exposed cohorts (to avoid potential informative censoring by eczema).

Table 12. Number of events, accumulated person-time, and HRs for cancer associated with eczema, Denmark. Analysis without censoring at eczema diagnosis.

					Hazard ratio and 99% confidence interval[1]	
	N	events	P-Y at risk	Rate per 1,000	Unadjusted	Mediation model
Any cancer						
Unexposed						
Exposed						
Lung cancer						
Unexposed						
Exposed						
Breast cancer						
Unexposed						
Exposed						
Prostate cancer						
Unexposed						
Exposed						
Melanoma skin cancer						
Unexposed						
Exposed						
Keratinocyte skin cancer						
Unexposed						
Exposed						
Lymphoma						
Unexposed						
Exposed						
Multiple myeloma						
Unexposed						
Exposed						
Leukemia						
Unexposed						
Exposed						
Meningioma						
Unexposed						
Exposed						
Cancer of the brain, hypophysis, corpus pineale and ductus craniopharyngealis						
Unexposed						
Exposed						

Spinal cord, cranial nerve and other CNS tumours						
Unexposed						
Exposed						

[1]Estimated hazard ratios from Cox regression with time since index date as underlying timescale, stratified by matched set.
Unadjusted: No adjustment.
Mediation model: Adjusted additionally for time-varying lifestyle-related diseases.

13. We will repeat the main analysis among those with an index date on or after 1 January 1996 to ensure at least 1 year of prescription history for patients.

Table 13. Number of events, accumulated person-time, and HRs for cancer associated with eczema, Denmark. Analysis restricting to index date on or after 1 January 1996.

					Hazard ratio and 99% confidence interval[1]	
	N	events	P-Y at risk	Rate per 1,000	Unadjusted	Mediation model
Any cancer						
Unexposed						
Exposed						
Lung cancer						
Unexposed						
Exposed						
Breast cancer						
Unexposed						
Exposed						
Prostate cancer						
Unexposed						
Exposed						
Melanoma skin cancer						
Unexposed						
Exposed						
Keratinocyte skin cancer						
Unexposed						
Exposed						
Lymphoma						
Unexposed						
Exposed						
Multiple myeloma						
Unexposed						
Exposed						
Leukemia						
Unexposed						
Exposed						

Meningioma							
Unexposed							
Exposed							
Cancer of the brain, hypophysis, corpus pineale and ductus craniopharyngealis							
Unexposed							
Exposed							
Spinal cord, cranial nerve and other CNS tumours							
Unexposed							
Exposed							

[1]Estimated hazard ratios from Cox regression with time since index date as underlying timescale, stratified by matched set.
Unadjusted: No adjustment.
Mediation model: Adjusted additionally for time-varying lifestyle-related diseases.

14. To explore the potential role of socioeconomic outcomes as confounders of the association, we will repeat the main analysis with additional adjustment for baseline educational level, partnership status, and income. We will restrict the analysis to those aged 30 years or older at index date, as people are more likely to have partnership and income recorded and to have attained their highest education level at this age. If this analysis suggests major confounding (and is sufficiently powered), we may explore this further using time-updated information on the socioeconomic variables for a larger part of the population (18 years or older at index date).

Table 14. Number of events, accumulated person-time, and HRs for cancer associated with eczema in persons aged 30 years or older on index date, Denmark. Analysis adjusting for baseline educational level, partnership status and income

					Hazard ratio and 99% confidence interval[1]		
	N	events	P-Y at risk	Rate per 1,000	Unadjusted	Adjusted model	Mediation model
Any cancer							
Unexposed							
Exposed							
Lung cancer							
Unexposed							
Exposed							
Breast cancer							
Unexposed							
Exposed							
Prostate cancer							

Unexposed
Exposed
Melanoma skin cancer
Unexposed
Exposed
Keratinocyte skin cancer
Unexposed
Exposed
Lymphoma
Unexposed
Exposed
Multiple myeloma
Unexposed
Exposed
Leukemia
Unexposed
Exposed
Meningioma
Unexposed
Exposed
Cancer of the brain, hypophysis, corpus pineale and ductus craniopharyngealis
Unexposed
Exposed
Spinal cord, cranial nerve and other CNS tumours
Unexposed
Exposed

[1]Estimated hazard ratios from Cox regression with time since index date as underlying timescale, stratified by matched set.
Unadjusted: No adjustment.
Adjusted: Adjusted for baseline educational level, partnership status, income.
Mediation model: Adjusted additionally for time-varying lifestyle-related diseases.

15. We will repeat the main analysis for specific cancers without censoring patients if they develop another individual cancer first.

Table 15. Number of events, accumulated person-time, and HRs for cancer associated with eczema, Denmark. Analysis without censoring at diagnosis of another specific individual cancer.

	Hazard ratio and 99% confidence interval[1]

	N	events	P-Y at risk	Rate per 1,000	Unadjusted	Mediation model
Lung cancer						
Unexposed						
Exposed						
Breast cancer						
Unexposed						
Exposed						
Prostate cancer						
Unexposed						
Exposed						
Melanoma skin cancer						
Unexposed						
Exposed						
Keratinocyte skin cancer						
Unexposed						
Exposed						
Lymphoma						
Unexposed						
Exposed						
Multiple myeloma						
Unexposed						
Exposed						
Leukemia						
Unexposed						
Exposed						
Meningioma						
Unexposed						
Exposed						
Cancer of the brain, hypophysis, corpus pineale and ductus craniopharyngealis						
Unexposed						
Exposed						
Spinal cord, cranial nerve and other CNS tumours						
Unexposed						
Exposed						

[1]Estimated hazard ratios from Cox regression with time since index date as underlying timescale, stratified by matched set.
Unadjusted: No adjustment.
Mediation model: Adjusted additionally for time-varying lifestyle-related diseases.

16. We will repeat the all-cancer analysis after excluding all skin cancer (non-melanoma and melanoma skin cancer) from the outcome definition.

**Table 16. Number of events, accumulated person-time, and HRs for cancer associated with eczema, Denmark. Excluding melanoma and non-melanoma skin cancer from the outcome**						
					Hazard ratio and 99% confidence interval[1]	
	N	events	P-Y at risk	Rate per 1,000	Unadjusted	Mediation model
Any cancer						
Unexposed						
Exposed						

[1]Estimated hazard ratios from Cox regression with time since index date as underlying timescale, stratified by matched set.
Unadjusted: No adjustment.
Mediation model: Adjusted additionally for time-varying lifestyle-related diseases.

17. Because of particular concerns about ascertainments bias for skin cancer, we will perform analyses using stage of melanoma at diagnosis as outcome (if feasible). Decreasing HR with increasing stage could suggest a shift with diagnosis at an earlier stage (as a proxy for ascertainment bias).

**Table 17. Number of events, accumulated person-time, and HRs for melanoma associated with eczema, Denmark. According to stage at diagnosis.**						
					Hazard ratio and 99% confidence interval[1]	
	N	events	P-Y at risk	Rate per 1,000	Unadjusted	Mediation model
Localized stage melanoma						
Unexposed						
Exposed						
Regional stage melanoma						
Unexposed						
Exposed						
Distant stage melanoma						
Unexposed						
Exposed						

[1]Estimated hazard ratios from Cox regression with time since index date as underlying timescale, stratified by matched set.
Unadjusted: No adjustment.
Mediation model: Adjusted additionally for time-varying lifestyle-related diseases.
