# Supplementary material for: Association Between Atopic Eczema and Cancer in England and Denmark
Source: JAMA Dermatol. 2020 Jun 24;156(10):1086–97. doi: 10.1001/jamadermatol.2020.1948 (PMC7315391; doi:10.1001/jamadermatol.2020.1948)
Supplement: Supplement 3. — eAppendix 1. Variable Definitions eAppendix 2. Study Populations eAppendix 3. Secondary Analyses eReferences. eFigure 1. England: Flowchart Illustrating Identification of Study Participants eFigure 2. Denmark: Flowchart Illustrating Identification of Study Participants eFigure 3. England: Association (HR [99% CI]* Comparing Individuals at Each Level of Eczema Disease Activity to Those Without Eczema) Between Atopic Eczema Disease Activity and Cancer eTable 1. Sensitivity Analyses eTable 2. England: Characteristics of the Study Population at Cohort Entry, for: the Overall Cohort, Individuals Included in the Model Additionally Adjusting for Potential Mediators (i.e. With No Missing BMI or Smoking Status Data), and for Individuals With Missing BMI or Smoking Status eTable 3. Denmark: Characteristics of the Study Population at Cohort Entry, for the Overall Cohort and for Individuals Aged 30 Years or Over (Included in Sensitivity Analysis Adjusting for Socioeconomic Status) eTable 4. England: Association (HR [99% CI]) Between Atopic Eczema and Cancer Outcomes: Comparing Risk of Cancer in Those With Atopic Eczema to Those Without eTable 5. Denmark: Association (HR [99% CI]) Between Atopic Eczema and Cancer Outcomes: Comparing Risk of Cancer in Those With Atopic Eczema to Those Without eTable 6. England: Hazard Ratios (99% CIs)* for Main and Sensitivity Analyses (After Adjusting for Calendar Period and IMD) eTable 7. Denmark: Hazard Ratios (99% CIs) for Main and Sensitivity Analyses eTable 8. England: Association (HR [99% CI]*) Between Severity of Atopic Eczema and Cancer Outcomes eTable 9. England: Association (HR [99% CI]*) Between Atopic Eczema Disease Activity and Cancer Outcomes eTable 10. England: Adjusted Hazard Ratios (99% CIs) for the Association Between Atopic Eczema and Cancer, Stratified by Sex (Adjusted for Calendar Period and IMD) eTable 11. Denmark: Adjusted Hazard Ratios (99% CIs) for the Association Between Atopic Eczema and Cancer, Stratified by Sex eTabl [file jamadermatol-e201948-s003.pdf]

Supplementary Online Content

Mansfield KE, Schmidt SAJ, Darvalics B, et al. Association between atopic eczema and cancer in England and Denmark. *JAMA Dermatol*. Published online June 24, 2020. doi:10.1001/jamadermatol.2020.1948

eAppendix 1. Variable Definitions

eAppendix 2. Study Populations

eAppendix 3. Secondary Analyses

eReferences.

eFigure 1. England: Flowchart Illustrating Identification of Study Participants

eFigure 2. Denmark: Flowchart Illustrating Identification of Study Participants

eFigure 3. England: Association (HR [99% CI]* Comparing Individuals at Each Level of Eczema Disease Activity to Those Without Eczema) Between Atopic Eczema Disease Activity and Cancer

eTable 1. Sensitivity Analyses

eTable 2. England: Characteristics of the Study Population at Cohort Entry, for: the Overall Cohort, Individuals Included in the Model Additionally Adjusting for Potential Mediators (i.e. With No Missing BMI or Smoking Status Data), and for Individuals With Missing BMI or Smoking Status

eTable 3. Denmark: Characteristics of the Study Population at Cohort Entry, for the Overall Cohort and for Individuals Aged 30 Years or Over (Included in Sensitivity Analysis Adjusting for Socioeconomic Status)

eTable 4. England: Association (HR [99% CI]) Between Atopic Eczema and Cancer Outcomes: Comparing Risk of Cancer in Those With Atopic Eczema to Those Without

eTable 5. Denmark: Association (HR [99% CI]) Between Atopic Eczema and Cancer Outcomes: Comparing Risk of Cancer in Those With Atopic Eczema to Those Without

eTable 6. England: Hazard Ratios (99% CIs)* for Main and Sensitivity Analyses (After Adjusting for Calendar Period and IMD)

eTable 7. Denmark: Hazard Ratios (99% CIs) for Main and Sensitivity Analyses

eTable 8. England: Association (HR [99% CI]*) Between Severity of Atopic Eczema and Cancer Outcomes

eTable 9. England: Association (HR [99% CI]*) Between Atopic Eczema Disease Activity and Cancer Outcomes

eTable 10. England: Adjusted Hazard Ratios (99% CIs) for the Association Between Atopic Eczema and Cancer, Stratified by Sex (Adjusted for Calendar Period and IMD)

eTable 11. Denmark: Adjusted Hazard Ratios (99% CIs) for the Association Between Atopic Eczema and Cancer, Stratified by Sex

eTable 12. England: Adjusted Hazard Ratios (99% CIs) for the Association Between Atopic Eczema Stratified by Current Age (Adjusted for Calendar Period and IMD)

eTable 13. Denmark: Adjusted Hazard Ratios (99% CIs) for the Association Between Atopic Eczema and Cancer, Stratified by Age

eTable 14. England: Adjusted Hazard Ratios (99% CIs) for the Association Between Atopic Eczema Stratified by Asthma (Adjusted for Calendar Period and IMD)

eTable 15. Denmark: Adjusted Hazard Ratios (99% CIs) for the Association Between Atopic Eczema and Cancer, Stratified by Asthma

eTable 16. Denmark: Codes Used to Define Atopic Eczema

eTable 17. Denmark: Codes Used to Define Outcomes (Cancer Overall and Specific Cancer Outcomes)

eTable 18. Denmark: Codes Used to Define Covariables (Confounders and Mediators)

This supplementary material has been provided by the authors to give readers additional information about their work.

© 2020 Mansfield KE et al. *JAMA Dermatology*.

eAppendix 1. VARIABLE DEFINITIONS

England

Complete code lists for all variables used in the English study are available for download (https://doi.org/10.17037/DATA.00001266).

Cancer diagnoses

We investigated the association between atopic eczema and cancer overall and the following site-specific cancers: 1) lung; 2) breast; 3) prostate; 4) pancreatic; 5) melanoma and non-melanoma (NMSC or keratinocyte cancer) skin cancers; 6) Hodgkin's lymphoma; 7) non-Hodgkin's lymphoma (NHL); 8) multiple myeloma; 9) leukaemia; and 10) central nervous system (CNS) (meningioma, glioma, and spinal cord, cranial nerve or other CNS tumours, when power was sufficient). We identified cancer diagnoses using the morbidity codes recorded in the Clinical Practice Research Datalink (CPRD, Read codes) and Hospital Episodes Statistics (HES, *International Classification of Diseases*, *Tenth Revision* [ICD-10] codes), and cause of death codes recorded in Office for National Statistics (ONS) data (ICD-9 before January 2001 and ICD-10 after January 2001). ICD-10 codes for specific cancers have been mapped to Read codes in previous work.[1] We used this Read code mapping (together with corresponding ICD-10 codes recorded in HES and ICD-9/ICD-10 codes recorded in ONS data) to identify codes representing clear cancer diagnoses (*i.e.*, codes for cancer, malignancies, or secondary cancers). Over 90% of cancers registered nationally can be identified in CPRD records; by also including ONS and HES data, we improved the completeness of the algorithm used to identify cancers.[2]

Atopic eczema

We identified atopic eczema using a validated algorithm[3] based on a record of one diagnostic morbidity code (recorded in either primary or secondary care) and at least two records (on separate days) for eczema therapy (recorded in primary care using Read morbidity codes or prescription data). Eczema therapy included primary care records of (1) morbidity codes for phototherapy and (2) prescriptions for topical emollients, corticosteroids or calcineurin inhibitors, or oral glucocorticoids, azathioprine, methotrexate, ciclosporin or mycophenolate.

We identified phototherapy using Read codes in primary care and Office of Population Censuses and Surveys (OPCS) procedure codes in secondary care. Systemic eczema treatment was identified using primary care prescription records. Hospital admissions for eczema were identified as any hospitalisation in which an ICD-10 code for eczema was recorded in the primary diagnostic position of any episode associated with the hospitalization.

© 2020 Mansfield KE et al. *JAMA Dermatology*.

Smoking and body mass index

We pragmatically defined smoking and body mass index (BMI) levels based on primary care records for these measures, using the status recorded closest to the cohort entry date. Records within -1 year to +1 month of the cohort entry date were regarded as the best, +1 month to +1 year from the cohort entry date as second best, the nearest prior to the year before the cohort entry date as the third best, and within +1 year from the cohort entry date as the worst. Smoking status was classified as: (1) current/ex-smoker; or (2) non-smoker. Read codes for BMI category were not used (because they are rarely recorded). Instead BMI was calculated using height and weight measures recorded closest to the cohort entry date. BMI was classified using the World Health Organisation categories, *i.e*., underweight [<18.5 kg/m^2], normal weight [18.5–24.9 kg/m^2], overweight [25.0–29.9 kg/m^2], and obese [≥30.0 kg/m^2]).

Harmful alcohol use

We defined harmful alcohol use based on primary care morbidity codes suggesting harmful or heavy alcohol use (including alcohol dependency codes and codes related to physical/psychological harm related to alcohol use) or a prescription for drugs used to maintain abstinence (acamprosate, disulfiram, or nalmefene). Individuals were defined as harmful alcohol users on the date of the first record of a relevant morbidity code or prescription.

Socioeconomic deprivation

We used the index of multiple deprivation (IMD) as a proxy for socioeconomic deprivation. IMD was measured using quintiles of patient-level IMD scores linked via the practice and patient postal code (when patient-level data was available for patients eligible for linkage to IMD data). While patient-level IMD data are available for the years 2004, 2007, 2010, and 2015, we used the 2007 version as this corresponded to the midpoint of the study period (January 1998 to March 2016). However, when patient-level data was unavailable, we used practice-level data from England in 2010 (the closest available to the 2007 version of patient-level data).

High-dose oral glucocorticoid use

We identified prescriptions for oral glucocorticoids (prednisolone, betamethasone, deflazacort, dexamethasone, hydrocortisone, methylprednisolone, prednisone, triamcinolone, and cortisone) and converted the prescribed daily dose to the prednisolone-equivalent dose (PED). High-dose oral glucocorticoid use was defined as a dose of 20 mg/day or higher PED. Individuals were defined as ever- or never-exposed to a high-dose oral glucocorticoid, with their status changing on the date of their first recorded prescription for a 20 mg/day or higher PED.

© 2020 Mansfield KE et al. *JAMA Dermatology*.

Immunosuppression

Our definition of immunosuppression included both immunosuppressive disorders (identified in both CPRD [Read morbidity codes] and HES [both ICD-10 morbidity codes and OPCS procedure codes]) and primary care prescriptions for drugs associated with immunosuppression.

Immunosuppressive disorders included infection with human immunodeficiency virus (HIV), haematopoietic stem cell or bone marrow transplant, myeloma or other unspecified cellular immune deficiencies (*e.g.*, pancytopenia), leukaemia, and lymphoma. Myeloma, leukaemia, and lymphoma were removed as covariates in analyses in which they comprised the outcome.

Our analyses included the following immunosuppressive drugs: biologic agents, chemotherapy drugs, cyclophosphamide, methotrexate, cyclosporin, azathioprine, mycophenolate, and calcineurin inhibitors. We did not include oral glucocorticoids, as their use is more likely to be transient.

© 2020 Mansfield KE et al. *JAMA Dermatology*.

Denmark

Complete code lists for all variables used in the Danish study are available in eTables 16-18.

Cancer diagnoses

We identified cancer diagnoses in the Danish Cancer Registry using ICD-10 codes. We also used ICD version 7 codes to exclude prevalent cancers recorded prior to 1978. The Danish Cancer Registry includes data on all incident cancers diagnosed in Denmark since 1943.[4] Among other variables, the registry includes information on morphology, histology, and cancer stage at time of diagnosis. We used the third edition of the *International Classification of Diseases for Oncology* (ICD-O-3) specifically to identify keratinocyte cancers. Reporting to the Danish Cancer Registry is mandatory and completeness is ensured through electronic notifications from multiple sources in the health care system, *i.e*., primary care, hospital departments (including departments of pathology), and death certificates. The validity of diagnoses is supported by the high proportion of tumours histologically verified and the very low proportion identified only by death certificates (0.1%). The high quality of diagnoses has been reported for lung cancer, breast cancer, and melanoma.[5,6]

Atopic eczema

We identified atopic eczema using ICD-8 and ICD-10 codes in the Danish National Patient Registry. First-time primary and secondary diagnoses were included from inpatient, outpatient and emergency room contacts with any Danish hospital department. A validation study showed a positive predictive value of 99% for atopic eczema diagnoses recorded at one dermatology department.[7] We identified systemic treatment of atopic eczema with methotrexate, cyclosporin, azathioprine, mycophenolate, methotrexate, and dupilumab using procedure codes in the Danish National Patient Registry and Anatomical Therapeutic Chemical (ATC) Classification codes in the Danish National Prescription Registry.

Lifestyle-related diseases

We identified lifestyle-related diseases (chronic obstructive pulmonary disease, hyperlipidaemia, hypertension, alcohol-related conditions, ischemic heart disease, hospital-diagnosed obesity, and type 2 diabetes) using codes in the Danish National Patient Registry. When possible, we aimed to increase completeness by supplementing this data with prescriptions for drugs used to treat these conditions, identified through the Danish National Prescription Registry. We included lipid-lowering drugs for hyperlipidaemia, antihypertensive drugs for hypertension, disulfiram for alcohol-related conditions, and oral antidiabetics for type 2 diabetes. Similarly, we used both diagnosis codes and relevant procedure codes (coronary artery bypass surgeries and percutaneous coronary interventions) to identify ischemic heart disease. We treated all lifestyle-related conditions as time-varying variables, with status changes occurring at first occurrence of a diagnosis code or prescription record).

© 2020 Mansfield KE et al. *JAMA Dermatology*.

Socioeconomic status

We used education and social registries maintained by Statistics Denmark to identify annual gross personal income, partner status, and education level at start of follow-up. We restricted analyses using these variables to patients aged 30 years or more, when income and partnership is more likely to be recorded and final education level is more likely to have been attained. We categorised **annual gross personal income** based on quartiles of income, as low (<120,022 Danish kroner), intermediate (120,022–203,325 Danish kroner), high (203,325–286264 Danish kroner), and very high (286,264+ Danish kroner). We identified **partnership status** using an algorithm generated by Statistics Denmark, which identifies a person as being single or in partnership (married or cohabitating) based on information on civil status, demographics, exact address, and kinship (*e.g.*, personal identifiers of children). We categorised **education level** as basic education (7–10 years), youth education (11–12 years), and higher education (13+ years).

Oral glucocorticoids

We identified oral glucocorticoid use from ATC codes in the Danish National Prescription Registry. As we were unable to identify the daily dose, we included any prescription for an oral glucocorticoid.

Immunosuppression

Our definition of immunosuppression paralleled the conditions and treatments used in England. We identified immunosuppression from diagnosis codes and procedure codes in the Danish National Patient Registry and ATC codes in the Danish National Prescription Registry.

Asthma

We identified asthma from hospital diagnoses in the Danish National Patient Registry.

© 2020 Mansfield KE et al. *JAMA Dermatology*.

eAppendix 2. STUDY POPULATIONS

England

In England, the population eligible for inclusion comprised all adults aged 18 years and older registered with CPRD practices eligible for linkage to HES/ONS databases between 2 January 1998 and 31 March 2016 (period of complete linkage to HES and ONS version 14) (**Figure 1**). All individuals had to be registered with a practice meeting CPRD quality control standards and had to have at least 12 months of registration prior to study entry (to allow adequate time for recording of baseline data in the patient record). We restricted our study population to adults due to limited follow up (mean follow up time = 5 years[8]) and the rarity of childhood cancer outcomes.

From this population, we identified individuals with atopic eczema (eczema-exposed cohort) using a validated algorithm[3] based on a record of one eczema diagnosis code from primary or secondary care and at least two primary care records (diagnosis code or prescription) for eczema therapy (**eAppendix 1**). We considered the date on which the individual fulfilled the full eczema algorithm (eczema diagnosis code or second eczema therapy record, whichever occurred first) to be the date of eczema diagnosis. Eczema-exposed individuals entered the cohort (index date) on the latest of the following: one year after date of registration with their primary care practice; date practice met CPRD quality control standards; start of study (2 January 1998); 18th birthday; or 12 months after the date of eczema diagnosis (to limit potential reverse causality).

We identified an age-, sex-, calendar period- and general practice-matched cohort of individuals without eczema. We randomly matched (without replacement) up to five individuals without atopic eczema for every individual with eczema in calendar date order (*i.e.*, individuals in the matched cohort were assigned first to individuals with eczema with earliest cohort entry to avoid time-related bias). Individuals without atopic eczema entered the cohort on the same date as the matched eczema-exposed individual (index date). We allowed a 15-year age difference for matching to maximise the generalisability of our cohort because of the small pool of eligible matched comparators (particularly among women and older people). We accounted for the wide age-matching window in our analysis by finely adjusting for age as the underlying timescale.

We followed individuals from cohort entry until the earliest of the following: no longer registered with practice, practice no longer contributing data to CPRD, death, end of study (31 March 2016), diagnosis of atopic eczema (matched cohort only), or cancer diagnosis (any cancer diagnosis, excluding non-melanoma skin cancer [NMSC]). We used morbidity coding in the CPRD and HES databases and cause-of-death coding in the ONS database to identify all records of cancer.

© 2020 Mansfield KE et al. *JAMA Dermatology*.

Denmark

In Denmark, we used data from linked nationwide registries: 1) The Danish National Patient Registry includes data on non-psychiatric hospital admissions from 1977, and all Danish hospital admissions, outpatient clinic appointments and emergency room contacts from 1994[9]; 2) The Danish Cancer Registry includes data on incident cancers diagnosed since 1943[4]; 3) The Civil Registration System includes demographic data (*e.g.*, civil status, vital status and address) for the entire population recorded from 1968[10]; 4) The Danish National Prescription Registry includes data on all prescriptions filled at community pharmacies since 1995[11]; and 5) A number of socioeconomic sources, including education level and income data, gathered by Statistics Denmark, the central statistical authority in Denmark.[12,13]

We used the Danish National Patient Registry to identify all individuals born in Denmark who received a hospital diagnosis of atopic eczema at any age between 1 January 1982 and 30 June 2016 (period of coverage from main data sources). Individuals entered the cohort on the date of their first atopic eczema diagnosis, recorded either during a hospital admission or an outpatient appointment. Individuals had to be living in Denmark on the date of cohort entry. The Danish atopic eczema cohort represents individuals with moderate-to-severe atopic eczema, as they were identified through hospital records.

We identified a matched cohort of individuals without atopic eczema by matching up to 10 individuals (with replacement) on sex and exact birth year to each individual with atopic eczema. Individuals entered the comparator cohort on the eczema diagnosis date of the matched eczema-exposed individual. They were required to be born and currently living in Denmark, with no previous atopic eczema diagnosis recorded on or before cohort entry.

We followed cohorts from cohort entry (date of atopic eczema diagnosis both for individuals with eczema and their matched comparators) to the earliest of the following: emigration, death, end of study (30 June 2017), diagnosis of atopic eczema (matched cohort only), or first-ever cancer diagnosis recorded in the Danish Cancer Registry.

© 2020 Mansfield KE et al. *JAMA Dermatology*.

eAppendix 3. SECONDARY ANALYSES

In secondary analyses, we investigated whether the association between atopic eczema and cancer was: (1) more pronounced in individuals with severe or active eczema and (2) modified by age, sex, or asthma. In Denmark, sample sizes were insufficient for analyses of eczema severity and activity. However, as we had no access to Danish primary care data (where mild eczema is managed), it is likely that people with eczema included in the Danish cohort had disease that required more intensive topical therapy and systemic treatment, corresponding to people classified with moderate/severe eczema in the English study population.

1. Eczema severity

In analyses examining eczema severity, we classified individuals with atopic eczema as having mild, moderate, or severe disease. We then compared their cancer risk to the risk among individuals without atopic eczema, using Cox regression to estimate HRs.

To define atopic eczema disease severity, we considered individuals to have mild disease by default. We classified individuals as having moderate disease from the first of: (1) a second potent topical corticosteroid prescription within one year; or (2) a first prescription for a topical calcineurin inhibitor. We defined severe atopic eczema from the first of: (1) use of phototherapy or systemic treatment for atopic eczema (excluding systemic glucocorticoids, as they may have been prescribed for coexisting asthma); (2) hospital admission for eczema (i.e., when eczema was recorded as the primary diagnosis of an admission); or (3) referral to a dermatologist. Severity was updated over time, i.e., individuals were classified as having mild eczema until the date they first satisfied the requirements of the moderate-eczema definition (unless they developed severe eczema). When (and if) a person satisfied the requirements of the definition for severe eczema, they switched to the severe category and remained in it for the rest of follow-up (similar to established procedures for defining severity in psoriasis studies[14]). At any given point during follow-up, individuals with atopic eczema therefore belonged to a single severity category.

2. Eczema activity

In addition to capturing maximum atopic eczema disease severity, we examined eczema activity, which reflects the proportion of life lived with eczema symptoms. It is possible that the association between atopic eczema and cancer differs between people experiencing severe eczema of short duration and those with long-standing mild eczema. We explored the role of atopic eczema disease activity in a *post-hoc* analysis, as results from our pre-specified analysis were suggestive of time-related bias (see 'Original algorithm' below). In the analysis of eczema activity, we classified individuals with atopic eczema into three categories based on the proportion of the 12 months preceding start of follow-up during which they experienced active disease (no active eczema; active disease for less than 50% of the time; or active disease for at least 50% of the time) and

© 2020 Mansfield KE et al. *JAMA Dermatology*.

compared their risk of cancer to individuals without eczema using Cox regression. As cohort entry in the English study was delayed until at least 12 months after the eczema diagnosis date, we identified eczema disease activity status in the 12 months before cohort entry. We defined disease activity as lasting for three months (considered a reasonable interval between prescriptions), starting from any medical record suggesting atopic eczema, including an eczema-related primary care consultation, a hospital admission, or a prescription for medication used to treat eczema. If an individual had a second medical record of eczema activity within the first three-month period, we extended the period of active disease by another three months from that second record (and so on for subsequent records). We then calculated disease activity as the percentage of time with active disease in the 12 months before start of follow-up.

We aimed to identify a measure of atopic eczema disease activity that captured long-term disease control (*e.g.* the proportion of an individual's life with eczema symptoms). In the past, eczema disease activity has been operationalised either as: (1) as a well-controlled week, defined as up to two days of symptoms or treatment in a week;[15] or (2) using flares,[16] defined as treatment intensification (*i.e.*, stepped-up treatment) and identifying those with frequent exacerbations as having active eczema. However, these approaches cannot easily be applied to electronic health record data.

2.1 Original algorithm

In our study protocol, we identified active atopic eczema as two or more primary care consultations, primary care prescriptions, or hospital records for eczema (diagnostic codes or records for eczema therapies [including prescribed medications and records for phototherapy]) within a one-year period.[17] Individuals started contributing person-time in the "active eczema" group on the date of the second consultation or prescription and were classified as having active eczema for the next 12 months, unless a new consultation/prescription was recorded, in which case they were classified as having active eczema for a further 12 months (and so on). Atopic eczema disease activity was then classified into one of three categories based on the percentage of total follow-up time with active eczema: (1) never active; 2) active during less than 50% of follow-up time; or (3) active during 50% or more of follow-up time.

However, we found that our protocol-defined eczema disease activity algorithm was prone to bias. Individuals with less follow up were more likely to have either all or none of their follow-up time defined as active. One reason is that it is necessary to survive to the second record indicating active disease, meaning that individuals with less follow-up time may be more likely to be classified as never having active disease (insufficient time to record the second active disease record). A second reason is that individuals with less follow-up time may also be more likely to have all of their observed time defined as active due to the algorithm classifying active disease in 12-month increments. Thus, if an individual has only 12 months of follow-up and they have two eczema records during this time, much of the time will be defined as active. This could lead to erroneously

© 2020 Mansfield KE et al. *JAMA Dermatology*.

high rates of cancer in those who either never have active disease or whose eczema is active for more than 50% of follow up time, as our original algorithm may differentially classify individuals with short follow-up time due to cancer in the 'never active group' or in the group whose disease is active during 50% or more of follow-up time.

Further, our original algorithm determined active disease status at any time point during follow-up based on data from the whole follow-up period. Therefore, the algorithm potentially misclassified activity status at an earlier time point based on what happened during a later time point.

2.2 Post hoc algorithm

Given the likelihood of time-related biases, we chose an alternative atopic eczema disease activity definition. We classified individuals with atopic eczema into three categories based on the proportion of the 12 months prior to the start of follow up spent with active disease (never active eczema, active eczema for less than 50% of the 12 months, or active eczema for at least 50% of the 12 months) and compared their risk of cancer to individuals without eczema using Cox regression. As cohort entry in the English study was delayed until at least 12 months after the eczema diagnosis date, we identified eczema disease activity status during the 12 months before cohort entry. We defined disease activity as lasting for three months (considered a reasonable interval between prescriptions) from any medical record suggesting eczema, including an eczema-related primary care consultation, a hospital admission, or therapy (prescribed medication or record of phototherapy). If an individual had a further record of activity within the three months, we extended the period of active disease by three months from the date of the second record (and so on for subsequent records). We then calculated disease activity as the percentage of time with active disease in the 12 months before start of follow-up.

Our post-hoc algorithm does not capture the totality of eczema disease experience throughout the follow-up period. For example, the algorithm may: (1) misclassify individuals with early active disease that remits during follow up as having highly active disease (leading to underestimation of the effect of active disease); or (2) misclassify individuals with early inactive disease that flares during follow up as having limited disease activity (again leading to underestimation of the effect of active disease). Overall, the effect of potential misclassification introduced by capturing a measure of eczema disease activity at cohort entry rather than throughout follow up (effectively an 'intention-to-treat' analysis) would be to bias our results to the null. Thus, any estimates of the effect of eczema disease activity using our new definition are likely to be conservative.

© 2020 Mansfield KE et al. *JAMA Dermatology*.

3. Effect modification

We performed analyses stratified by age, sex, or asthma (time-varying variable with status changing at the time of the first asthma diagnosis record) to examine whether the association between atopic eczema and cancer differed between subgroups of these characteristics (*i.e.*, effect modification).

© 2020 Mansfield KE et al. *JAMA Dermatology*.

eREFERENCES.

1. Bhaskaran K, Douglas I, Forbes H, Dos-Santos-Silva I, Leon DA, Smeeth L. Body-mass index and risk of 22 specific cancers: a population-based cohort study of 5.24 million UK adults. *Lancet*. 2014;6736(14):60892-60898. http://dx.doi.org/10.1016/S0140-6736(14)60892-8.

2. Boggon R, van Staa T-P, Chapman M, Gallagher AM, Hammad TA, Richards MA. Cancer recording and mortality in the General Practice Research Database and linked cancer registries. *Pharmacoepidemiol Drug Saf*. 2013;22:168-175. doi:10.1002/pds.3374

3. Abuabara K, Magyari AM, Hoffstad O, et al. Development and Validation of an Algorithm to Accurately Identify Atopic Eczema Patients in Primary Care Electronic Health Records from the UK. *J Invest Dermatol*. 2017;137(8):1655-1662. doi:10.1016/j.jid.2017.03.029

4. Gjerstorff ML. The Danish Cancer Registry. *Scand J Public Health*. 2011;39(7):42-45. doi:10.1177/1403494810393562

5. Friis S, Jørgensen T, Mellemkjær L. *Validation of the Danish Cancer Registry and Selected Clinical Cancer Databases*.; 2012.

6. Pedersen SA, Schmidt SAJ, Klausen S, et al. Melanoma of the skin in the danish cancer registry and the danish melanoma database. *Epidemiology*. 2018;29(3):442-447. doi:10.1097/EDE.0000000000000802

7. Schmidt S, Olsen M, Schmidt M, et al. Atopic dermatitis and risk of atrial fibrillation or flutter: a 35-year follow-up study. *J Am Acad Dermatol*. 2019;(provisionally accepted).

8. Herrett E, Gallagher AM, Bhaskaran K, et al. Data Resource Profile: Clinical Practice Research Datalink (CPRD). *Int J Epidemiol*. 2015;44(3):1-10. doi:10.1093/ije/dyv098

9. Schmidt M, Schmidt SAJ, Sandegaard JL, Ehrenstein V, Pedersen L, Sørensen HT. The Danish National patient registry: A review of content, data quality, and research potential. *Clin Epidemiol*. 2015;7:449-490. doi:10.2147/CLEP.S91125

10. Schmidt M, Pedersen L, Sørensen HT. The Danish Civil Registration System as a tool in epidemiology. *Eur J Epidemiol*. 2014;29(8):541-549. doi:10.1007/s10654-014-9930-3

11. Pottegård A, Schmidt SAJ, Wallach-Kildemoes H, Sørensen HT, Hallas J, Schmidt M. Data resource profile: The Danish National Prescription Registry. *Int J Epidemiol*. 2017;46(3):798. doi:10.1093/ije/dyw213

12. Jensen VM, Rasmussen AW. Danish education registers. *Scand J Public Health*. 2011;39(7):91-94. doi:10.1177/1403494810394715

13. Baadsgaard M, Quitzau J. Danish registers on personal income and transfer payments. *Scand J Public Health*. 2011;39(7):103-105. doi:10.1177/1403494811405098

14. Gelfand JM, Troxel AB, Lewis JD, et al. The Risk of Mortality in Patients With Psoriasis. *Arch Dermatol*. 2007;143(12):1493-1499.

15. Langan SM, Stuart B, Bradshaw L, Schmitt J, Williams HC, Thomas KS. Measuring long-term disease control in patients with atopic dermatitis: A validation study of well-controlled weeks. *J Allergy Clin Immunol*. 2017;140(6):1580-1586. doi:10.1016/j.jaci.2017.02.043

16. Langan SM, Thomas KS, Williams HC. What Is Meant by a “Flare” in Atopic Dermatitis? *Arch Dermatol*. 2006;142(9):1190-1196. doi:10.1001/archderm.142.9.1190

17. Silverwood RJ, Forbes HJ, Abuabara K, et al. Severe and predominantly active atopic eczema in adulthood and long term risk of cardiovascular disease: population based cohort study. *Bmj*. 2018;361:k1786. doi:10.1136/bmj.k1786

18. Mathur R, Bhaskaran K, Chaturvedi N, et al. Completeness and usability of ethnicity data in UK-based primary care and hospital databases. *J Public Health (Oxf)*. 2013;36(4):684-692. doi:10.1093/pubmed/fdt116

19. Cardwell CR, Shields MD, Carson DJ, Patterson CC. A meta-analysis of the association between childhood type 1 diabetes and atopic disease. *Diabetes Care*. 2003;26(9):2568-2574. doi:10.2337/diacare.26.9.2568

© 2020 Mansfield KE et al. *JAMA Dermatology*.

eFigure 1. England: Flowchart illustrating identification of study participants.

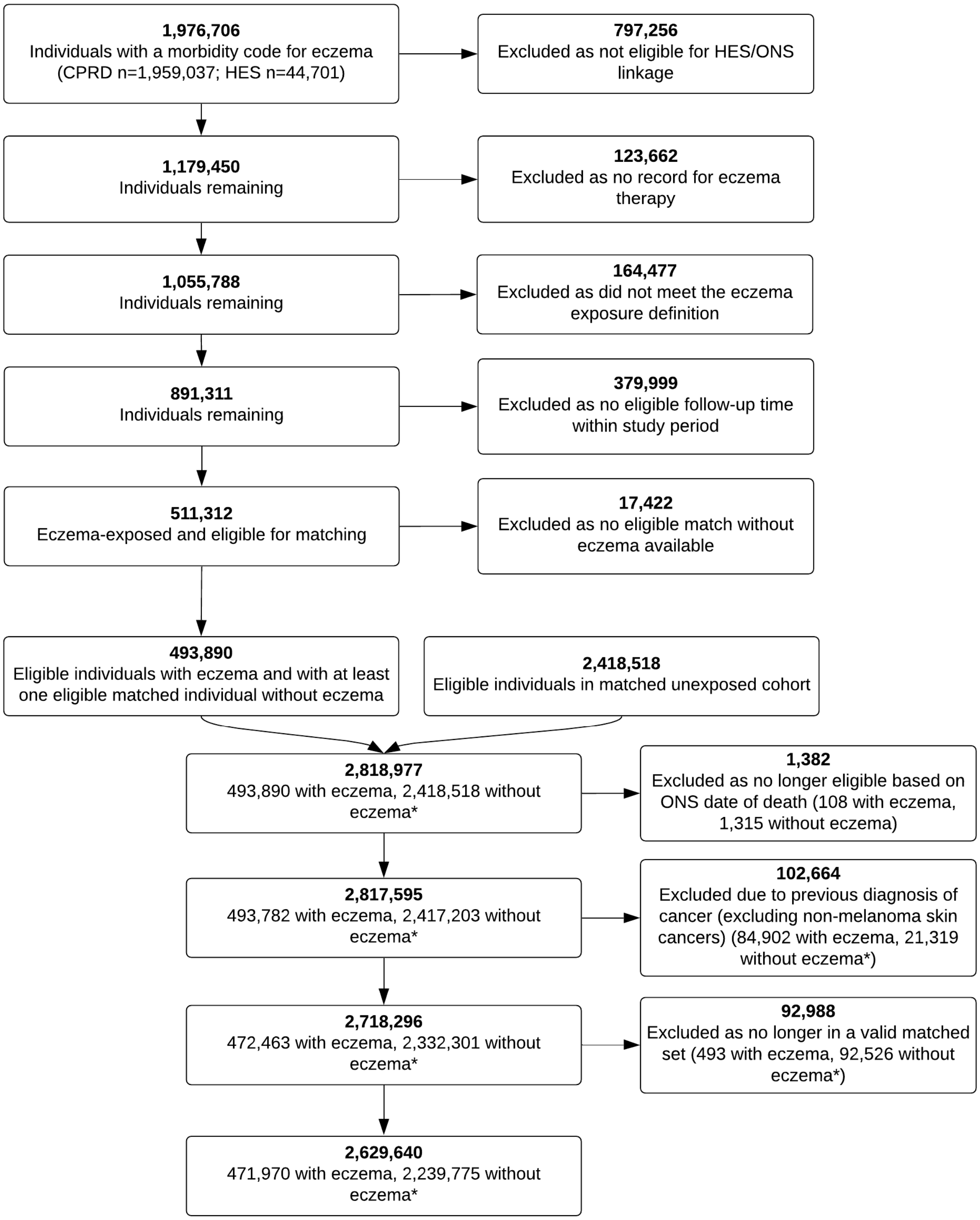

*NB: numbers of individuals with and without eczema do not necessarily sum to the total number of individuals included, as individuals with eczema could be included in the matched comparison cohort up until the date of their first eczema diagnosis.
Abbreviations: CPRD: Clinical Practice Research Datalink; HES: Hospital Episode Statistics; ONS: Office for National Statistics.

© 2020 Mansfield KE et al. *JAMA Dermatology*.

eFigure 2. Denmark: Flowchart illustrating identification of study participants.

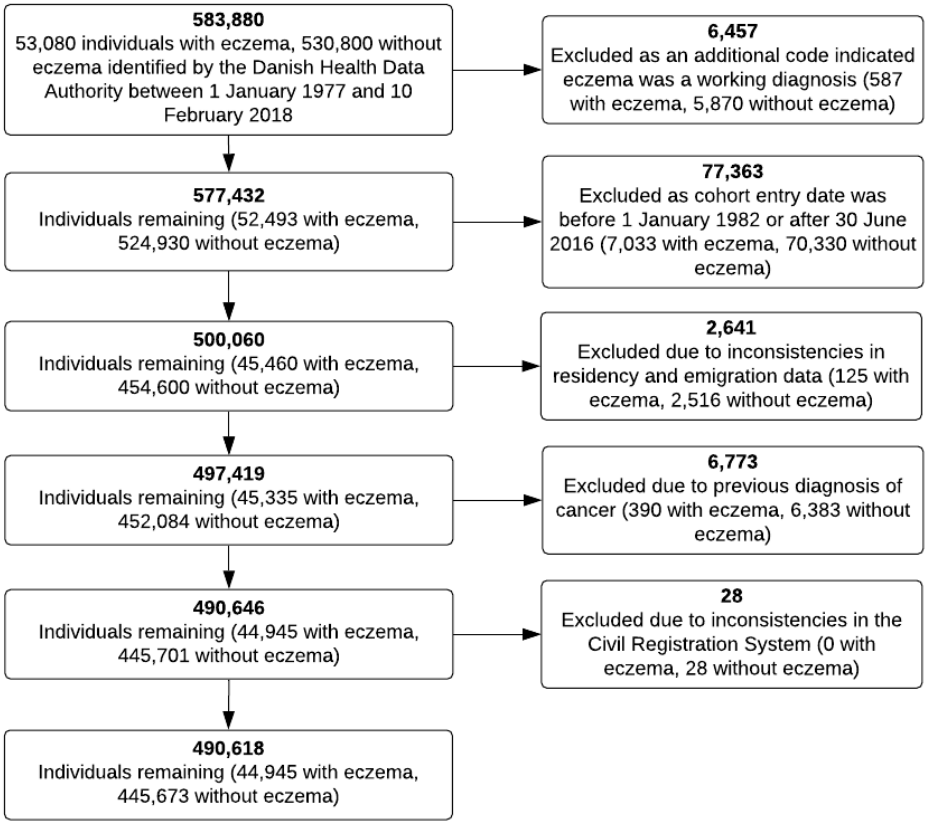

© 2020 Mansfield KE et al. *JAMA Dermatology*.

eFigure 3. England: Association (HR [99% CI]* comparing individuals at each level of eczema disease activity to those without eczema) between atopic eczema disease activity and cancer.

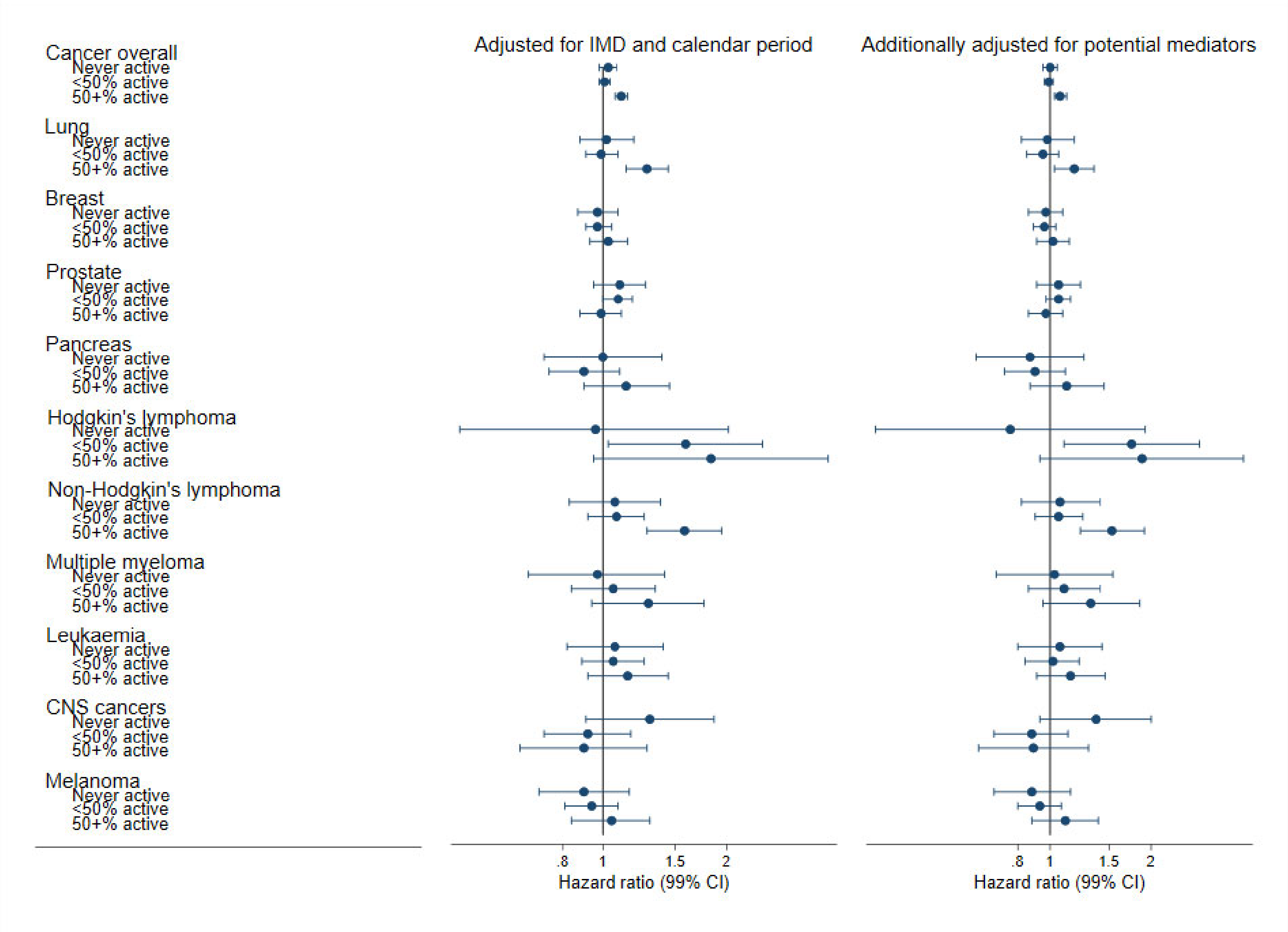

*Estimated hazard ratios obtained from Cox regression using current age as the underlying timescale, stratified by matched set (matched on age at cohort entry, sex, general practice, and date of cohort entry). All models fitted to individuals with complete data for all variables included in each model and from valid matched sets, including one eczema-exposed individual and at least one unexposed individual without eczema.

All models implicitly adjusted for sex, date of cohort entry, and practice (due to stratification by matched set), and age (due to underlying timescale).

Potential mediators: Body mass index, harmful alcohol use, and smoking.

Abbreviations: IMD: Index of Multiple Deprivation; CNS: central nervous system.

Specific CNS tumours (meningioma, brain neoplasm, and other CNS tumours) were too rare to consider individually.

© 2020 Mansfield KE et al. *JAMA Dermatology*.

eTable 1. Sensitivity analyses.

Sensitivity analysis description	Justification	England*	Denmark*
Restricted to individuals with a **3-year cancer-free window** following eczema diagnosis (main analysis in English studies required a 12-month window, Danish studies did not have this 12-month window to maximise study power). This analysis was restricted to individuals with at least three years of follow-up in the CPRD prior to cohort entry, with no record of a cancer diagnosis prior to cohort entry.	We included a 12-month cancer-free window following eczema diagnosis in the English studies to limit **reverse causality** as an explanation for any observed association (to maximise power, given the smaller sample size, we did not do this for the main analysis for the Danish studies). By increasing this window to 3 years, we aimed to account for any lag between cancer onset and diagnosis, and therefore to further reduce reverse causality.	Yes (E1)	Yes (D1)
Restricted to individuals with **at least one consultation** with their GP in the year prior to cohort entry.	To exclude practice **non-attenders.**	Yes (E2)	No
Restricted to individuals with **a diagnosis of newly active atopic eczema in the exposed cohort** (exposed individuals defined as those who joined the cohort when they first fulfilled the study's diagnostic criteria after the start of the study period) and their matched sets.	**Covariates measured at entry precede atopic eczema** onset, so will not be on the causal pathway between atopic eczema and cancer outcomes.	Yes (E3)	No
The main analysis was repeated using a **redefined cohort** (different unexposed pool, exposed individuals unchanged, unless unmatched), where: (1) the pool of unexposed people also included individuals with an atopic eczema diagnosis but without two treatments for the entire duration of their follow-up; and (2) individuals in the exposed cohort (with an atopic eczema diagnosis and two treatments) were classified as unexposed up until cohort entry (*i.e.*, at the time of their atopic eczema diagnosis or their two treatments, whichever occurred later). This cohort was matched separately to the main analysis cohort.	To explore the sensitivity of the results to the **definition of the exposure.**	Yes (E4)	No
The main analysis was repeated using a **second redefined cohort,** in which exposed individuals had only an atopic eczema diagnosis (*i.e.*, without requiring two atopic eczema treatments) and these individuals were eligible for inclusion in the unexposed cohort up until their atopic eczema diagnosis. (Some individuals may have had childhood atopic eczema; however, they might not have recorded treatment codes if they registered with their GP during adulthood. These individuals therefore may have been excluded erroneously from the exposed cohort in the primary analysis). This cohort was matched separately to the main analysis cohort.	To explore the sensitivity of the results to the **definition of the exposure.**	Yes (E5)	No
We repeated the analysis after removing the **censoring criterion of an atopic eczema** diagnosis for members of the matched cohort.	To avoid potential **informative censoring** by atopic eczema in the matched cohort.	No	Yes (D5)
Restricted to a subset of individuals **registered from 2006 onwards**, and additionally adjusting for **ethnicity** (White, South Asian, Black, other, or mixed), identified from CPRD and HES data using a previously developed algorithm[18] Records for ethnicity became more complete following the introduction of remuneration for including ethnicity data in the Quality and Outcomes Framework).	To examine whether the omission of ethnicity as a covariate in the main analysis introduced bias.	Yes (E6)	No
Restricted to individuals entering **the cohorts from 2004 onwards**.	To account for changes in diagnostic and coding practices over time – specifically those introduced by the Quality and Outcomes Framework in 2004. This is likely to be more important for cancer outcomes and some covariates. However, as there are no specific dermatology indicators in the Quality and Outcomes Framework, it is unlikely that eczema coding was affected).	Yes (E7)	No
Additionally including **diabetes mellitus** as a covariate. Diabetes mellitus was classified as absent, type 1, type 2, or type unknown, based on primary or secondary care diabetes diagnoses (using definite diagnostic codes only). Onset of diabetes was defined as the date of the earliest recorded diagnostic code. Diabetes mellitus is part of the Quality and Outcomes Framework, which remunerates primary care practices for recording specific management and monitoring practices for some chronic conditions. Therefore, diagnostic coding is likely for individuals with diagnosed diabetes, eliminating the need to supplement the definition with prescriptions for anti-diabetic drugs or possible diabetes codes.	Our rationale for including diabetes mellitus as a covariate and stratifying by diabetes type (rather than simply classifying as present or absent) is as follows: (1) Type 1 diabetes mellitus is immune-mediated (resulting from autoimmune destruction of pancreatic beta-cells); (2) Research suggests that there is a link between diabetes and cancer.[19] (3) We believe that any link between eczema and cancer will be via an immune mechanism. Therefore, it seems important to consider diabetes as a potential confounder in the relationship between eczema and cancer. However, as this is a somewhat tenuous link, we only adjusted for diabetes in this sensitivity analysis.	Yes (E8)	Adjusted for diabetes (as a proxy measure of lifestyle factors) in analysis adjusting for potential mediators

© 2020 Mansfield KE et al. *JAMA Dermatology*.

Repeated main analysis using a cohort that was matched **without allowing for a 12-month cancer-free window** starting from the eczema diagnosis date (*i.e.* start of observation for eczema-exposed individuals was the earliest of: diagnosis date + one year, current registration date + one year, up-to-standard date, or 18th birthday, whichever occurred first).	To be comparable with methods used with **Danish** data.	Yes (E9)	No
Additionally adjusting for **immunosuppression**. Immunosuppression was defined using coding of immunosuppressive disorders and records of prescriptions for drugs leading to immunosuppression (excluding oral corticosteroid use, as this is likely to be transient). Immunosuppressive disorders included HIV, haematopoietic stem cell or bone marrow transplant, myeloma, or other unspecified cellular immune deficiencies (*e.g.,* pancytopenia), leukaemia, and lymphoma. Myeloma, leukaemia, and lymphoma were removed from the definition of immunosuppression in the respective analyses in which they were outcomes.	As immunosuppression might mediate the relationship between eczema and specific cancers, we only adjusted for immunosuppression in a sensitivity analysis.	Yes (E10)	Yes (D2)
Additionally adjusting for **oral glucocorticoids**. We adjusted for oral glucocorticoids as a time-updated variable categorized as ever/never prescribed, with status changing at the time of the first prescription.	Oral glucocorticoids may be prescribed more frequently to people with atopic eczema because of coexisting asthma and may also increase the risk of certain cancers.	Yes (E11)	Yes (D3)
Additionally adjusting for **systemic therapies**. We adjusted for systemic therapy (methotrexate, ciclosporin, azathioprine, or mycophenolate), as a time-updated variable categorized as ever/never prescribed, with status changing at the time of the first prescription. Oral glucocorticoids were not included.	Systemic immunosuppressive drugs (cyclosporin, azathioprine, mycophenolate and methotrexate) may be prescribed for severe atopic eczema and may also affect cancer risk.	Yes (E12)	Yes (D4)
We repeated the overall cancer analysis after **excluding any skin cancer diagnoses** from the overall cancer outcome definition.	To explore potential **ascertainment bias** introduced by investigating skin cancer in people with and without atopic eczema. People with eczema are more likely to be diagnosed with skin cancer as clinicians examine skin more carefully in those with existing skin disease.	Yes (E13)	Yes (D5)
Repeated specific cancer analyses **without censoring at diagnosis of other types of cancer** (*i.e.*, censoring only at diagnosis of the specific cancer under investigation).	In the main analyses we censored at the time of any cancer diagnosis, as some cancers may represent secondary sites of an original cancer (rather than a primary cancer diagnosis). We repeated the analysis to explore the impact of not censoring at diagnosis of other types of cancer.	Yes (E14)	No
Repeated the main analysis after restriction to individuals with **complete smoking and BMI data** in valid matched sets.	We undertook a complete case analysis for analyses additionally adjusting for potential mediators (*i.e.* smoking status, BMI, and harmful alcohol use). We therefore repeated the main analysis in individuals with complete BMI and smoking data after adjusting for quintile of IMD score and calendar period only (no further adjustment for potential mediators) to ensure that any difference between effect estimates from analyses adjusted for confounders and from analyses additionally adjusted for potential mediators were due to the additional covariables adjusted for and not a result of the restriction of the study sample.	Yes (E15)	No
Repeated analysis after **restriction** to individuals with an **index date on or after 1 January 1996**.	To limit left-censoring of variables (*i.e.,* incorrect identification of variables due to insufficient data to capture them) by ensuring at least 1 year of registration history for all registries prior to cohort entry (index) date.	No	Yes (D6)
Additionally adjusting for **socioeconomic status** by adding baseline educational level (short, medium, long, missing), partnership status (married/cohabiting, single) and gross personal income (low, intermediate, high, very high) to the minimally adjusted model. We restricted the analysis to those **aged 30 years or older** on the cohort entry date, as people are more likely to have recorded partnership and income information and to have attained their highest education level by this age.	To explore the potential role of socioeconomic outcomes as confounders of the association between atopic eczema and cancer. Prior to this analysis, we also repeated the main analysis for individuals aged 30 years and older without adjusting for SES to ensure that any differences in effect estimates after adjusting for SES variables were due to SES and not to restriction to people over 30 years old (D7a).	No	Yes (D7)

* Note: The numbers in brackets after the contents (Yes/No) of the England/Denmark columns refer to numbered analyses in the sensitivity analysis results tables: **Supplementary eTables 6 and 7**.

© 2020 Mansfield KE et al. *JAMA Dermatology*.

eTable 2. England: Characteristics of the study population at cohort entry, for: the overall cohort, individuals included in the model additionally adjusting for potential mediators (i.e. with no missing BMI or smoking status data), and for individuals with missing BMI or smoking status. Values are n (%) unless stated otherwise.

	Overall cohort		Sample included in model adjusting for potential mediators		Individuals with missing BMI status		Individuals with missing smoking status	
	With atopic eczema	Without atopic eczema	With atopic eczema	Without atopic eczema	With atopic eczema	Without atopic eczema	With atopic eczema	Without atopic eczema
**Number**	471,970	2,239,775	392,644	1,588,775	73,770	442,225	12,567	131,622
**Follow up[a]**								
Total person-years	2,864,446	12,601,393	2,538,790	9,680,745	300,921	1,839,218	36,822	477,319
Median (IQR) duration of follow-up (years)	4.8 (1.9-9.2)	4.2 (1.7-8.6)	5.3 (2.2-9.9)	4.8 (1.9-9.3)	2.8 (1.2-5.9)	2.8 (1.1-6.1)	1.7 (0.8-4.0)	2.3 (0.8-5.1)
Mean (SD) duration of follow-up (years)	6.1 (4.9)	5.6 (4.8)	6.5 (5.0)	6.1 (5.0)	4.1 (3.9)	4.2 (4.0)	2.9 (3.2)	3.6 (3.8)
**Female (%)**	276,510 (58.6%)	1,301,074 (58.1%)	244,836 (62.4%)	1,014,382 (63.8%)	29,341 (39.8%)	185,366 (41.9%)	4,627 (36.8%)	49,685 (37.7%)
**Age (years)[b]**								
18-44	262,119 (55.5%)	1,292,565 (57.7%)	189,676 (48.3%)	709,650 (44.7%)	60,150 (81.5%)	336,446 (76.1%)	9,901 (78.8%)	95,872 (72.8%)
45-54	115,510 (24.5%)	563,375 (25.2%)	111,329 (28.4%)	507,643 (32.0%)	5,198 (7.0%)	51,414 (11.6%)	683 (5.4%)	17,333 (13.2%)
65-69	94,341 (20.0%)	383,835 (17.1%)	91,639 (23.3%)	371,482 (23.4%)	8,422 (11.4%)	54,365 (12.3%)	1,983 (15.8%)	18,417 (14.0%)
**Quintiles IMD[c]**								
1 (least deprived)	113,598 (24.1%)	531,707 (23.7%)	93,972 (23.9%)	379,047 (23.9%)	18,318 (24.8%)	105,101 (23.8%)	3,145 (25.0%)	31,317 (23.8%)
2	107,613 (22.8%)	509,274 (22.7%)	89,541 (22.8%)	362,571 (22.8%)	16,853 (22.8%)	99,694 (22.5%)	2,709 (21.6%)	28,305 (21.5%)
3	92,864 (19.7%)	441,451 (19.7%)	76,965 (19.6%)	312,245 (19.7%)	14,606 (19.8%)	86,935 (19.7%)	2,700 (21.5%)	25,869 (19.7%)
4	90,112 (19.1%)	428,193 (19.1%)	75,332 (19.2%)	304,093 (19.1%)	13,756 (18.6%)	83,333 (18.8%)	2,320 (18.5%)	25,372 (19.3%)
5 (most deprived)	67,783 (14.4%)	329,150 (14.7%)	56,834 (14.5%)	230,819 (14.5%)	10,237 (13.9%)	67,162 (15.2%)	1,693 (13.5%)	20,759 (15.8%)
**Body mass index (kg/m^2)[d]**								
Underweight (<20)	34,065 (7.2%)	165,212 (7.4%)	33,070 (8.4%)	136,066 (8.6%)	n/a	n/a	212 (1.7%)	1,622 (1.2%)
Normal (20-24)	153,822 (32.6%)	721,037 (32.2%)	151,590 (38.6%)	630,452 (39.7%)	n/a	n/a	441 (3.5%)	3,670 (2.8%)
Overweight (25-29)	127,144 (26.9%)	570,179 (25.5%)	125,800 (32.0%)	512,981 (32.3%)	n/a	n/a	324 (2.6%)	2,373 (1.8%)
Obese (30+)	83,169 (17.6%)	341,122 (15.2%)	82,184 (20.9%)	309,276 (19.5%)	n/a	n/a	260 (2.1%)	1,434 (1.1%)
Missing	73,770 (15.6%)	442,225 (19.7%)	n/a	n/a	n/a	n/a	11,330 (90.2%)	122,523 (93.1%)
**Smoking[d]**								
Non-smoker	239,072 (50.7%)	1,132,798 (50.6%)	196,217 (50.0%)	826,258 (52.0%)	40,203 (54.5%)	195,187 (44.1%)	n/a	n/a
Current or ex-smoker	220,331 (46.7%)	975,355 (43.5%)	196,427 (50.0%)	762,517 (48.0%)	22,237 (30.1%)	124,515 (28.2%)	n/a	n/a
Missing	12,567 (2.7%)	131,622 (5.9%)	n/a	n/a	11,330 (15.4%)	122,523 (27.7%)	n/a	n/a
**Harmful alcohol use[e]**	12,812 (2.7%)	49,844 (2.2%)	11,788 (3.0%)	40,555 (2.6%)	936 (1.3%)	5,212 (1.2%)	108 (0.9%)	833 (0.6%)
**Ethnicity**								
White	362,553 (76.8%)	1,619,242 (72.3%)	312,137 (79.5%)	1,220,774 (76.8%)	46,416 (62.9%)	248,028 (56.1%)	6,322 (50.3%)	50,431 (38.3%)
South Asian	19,709 (4.2%)	77,005 (3.4%)	17,357 (4.4%)	57,618 (3.6%)	2,181 (3.0%)	11,258 (2.5%)	219 (1.7%)	2,072 (1.6%)
Black	9,897 (2.1%)	47,164 (2.1%)	8,302 (2.1%)	35,146 (2.2%)	1,512 (2.0%)	7,672 (1.7%)	194 (1.5%)	1,684 (1.3%)
Other	5,163 (1.1%)	26,519 (1.2%)	4,338 (1.1%)	18,375 (1.2%)	781 (1.1%)	5,379 (1.2%)	128 (1.0%)	1,289 (1.0%)
Mixed	2,414 (0.5%)	10,805 (0.5%)	1,855 (0.5%)	6,876 (0.4%)	517 (0.7%)	2,524 (0.6%)	87 (0.7%)	530 (0.4%)

© 2020 Mansfield KE et al. *JAMA Dermatology*.

Not stated or missing	72,234 (15.3%)	459,040 (20.5%)	48,655 (12.4%)	249,986 (15.7%)	22,363 (30.3%)	167,364 (37.8%)	5,617 (44.7%)	75,616 (57.4%)
Comorbidities[e]								
Asthma	117,510 (24.9%)	283,344 (12.7%)	95,614 (24.4%)	200,025 (12.6%)	19,879 (26.9%)	50,157 (11.3%)	2,439 (19.4%)	8,900 (6.8%)
Immunosuppression	7,971 (1.7%)	22,306 (1.0%)	7,144 (1.8%)	18,267 (1.1%)	747 (1.0%)	2,453 (0.6%)	96 (0.8%)	445 (0.3%)
Diabetes mellitus[e]								
No diabetes	443,805 (94.0%)	2,137,544 (95.4%)	365,841 (93.2%)	1,499,536 (94.4%)	72,732 (98.6%)	437,073 (98.8%)	12,362 (98.4%)	130,380 (99.1%)
Type I	4,067 (0.9%)	14,630 (0.7%)	3,866 (1.0%)	12,258 (0.8%)	123 (0.2%)	632 (0.1%)	33 (0.3%)	186 (0.1%)
Type II	16,389 (3.5%)	61,142 (2.7%)	15,967 (4.1%)	55,639 (3.5%)	287 (0.4%)	1,944 (0.4%)	75 (0.6%)	555 (0.4%)
Type unspecified	7,709 (1.6%)	26,459 (1.2%)	6,970 (1.8%)	21,342 (1.3%)	628 (0.9%)	2,576 (0.6%)	97 (0.8%)	501 (0.4%)
Prescriptions[e]								
High-dose oral glucocorticoids	59,437 (12.6%)	106,924 (4.8%)	51,341 (13.1%)	84,905 (5.3%)	7,164 (9.7%)	12,365 (2.8%)	848 (6.7%)	2,001 (1.5%)
Systemic drugs for atopic eczema	5,177 (1.1%)	12,805 (0.6%)	4,689 (1.2%)	10,835 (0.7%)	435 (0.6%)	1,106 (0.3%)	53 (0.4%)	186 (0.1%)
Calendar period[b]								
1998-2001	125,996 (26.7%)	608,483 (27.2%)	84,296 (21.5%)	198,318 (12.5%)	15,345 (20.8%)	105,747 (23.9%)	4,961 (39.5%)	49,940 (37.9%)
2002-2004	61,678 (13.1%)	295,393 (13.2%)	51,242 (13.1%)	186,049 (11.7%)	8,368 (11.3%)	55,916 (12.6%)	1,978 (15.7%)	21,305 (16.2%)
2005-2007	76,947 (16.3%)	365,458 (16.3%)	60,239 (15.3%)	238,744 (15.0%)	10,939 (14.8%)	69,955 (15.8%)	1,287 (10.2%)	18,256 (13.9%)
2008-2010	81,889 (17.4%)	385,874 (17.2%)	68,471 (17.4%)	299,373 (18.8%)	13,614 (18.5%)	77,521 (17.5%)	1,395 (11.1%)	16,480 (12.5%)
2011-2013	81,740 (17.3%)	382,322 (17.1%)	71,129 (18.1%)	353,763 (22.3%)	16,182 (21.9%)	84,618 (19.1%)	1,533 (12.2%)	14,659 (11.1%)
2014-2016	43,720 (9.3%)	202,245 (9.0%)	57,267 (14.6%)	312,528 (19.7%)	9,322 (12.6%)	48,468 (11.0%)	1,413 (11.2%)	10,982 (8.3%)

Individuals could contribute data as both eczema exposed and unexposed.

Follow-up based on censoring at the earliest of: death, no longer registered with practice, practice no longer contributing to CPRD, or any cancer diagnosis (excluding non-melanoma skin cancer)

Abbreviations: IQR: Interquartile range; SD: Standard deviation; IMD: Index of multiple deprivation. BMI: Body mass index.

a. Follow-up based on censoring at the earliest of: death, no longer registered with practice, practice no longer contributing to CPRD, or any cancer diagnosis (excluding non-melanoma skin cancer).
b. Age or calendar period at cohort entry.
c. IMD based on individual-level data (from 2007) if available, supplemented with practice-level data (from 2010) if individual-level data not available.
d. Smoking and BMI based on records closest to index date.
e. Based on records on or before cohort entry.

© 2020 Mansfield KE et al. *JAMA Dermatology*.

eTable 3. Denmark: Characteristics of the study population at cohort entry, for the overall cohort and for individuals aged 30 years or over (included in sensitivity analysis adjusting for socioeconomic status). Values are n (%) unless stated otherwise.

	Overall cohort		Cohort aged 30 or more	
	With atopic eczema	Without atopic eczema	With atopic eczema	Without atopic eczema
Number	44,945	445,673	6,939	67,179
Follow up				
Total person-years	639,121	6,358,286	73,786	746,311
Median (IQR) duration of follow-up (years)	14.3 (6.3-20.8)	14.3 (6.3-20.8)	10.9 (4.4-16.3)	11.3 (4.7-16.8)
Mean (SD) duration of follow-up (years)	14.3 (9.1)	14.3 (9.1)	10.9 (7.6)	11.3 (7.7)
Female (%)	22,826 (50.8%)	226,323 (50.8%)	4,034 (58.1%)	39,012 (58.1%)
Age (years)				
<18	31,772 (70.2%)	316,396 (71.0%)	n/a	n/a
18–44	9,875 (22.0%)	98,181 (22.0%)	3,641 (52.5%)	36,083 (53.7%)
45–65	2,418 (5.4%)	23,308 (5.2%)	2,418 (34.8%)	23,308 (34.7%)
65+	880 (2.0%)	7,788 (1.7%)	880 (12.7%)	7,788 (11.6%)
Calendar year for index date				
1982–1999	17,345 (38.6%)	172,250 (38.6%)	1,957 (28.2%)	19,168 (28.5%)
2000–2004	7,743 (17.2%)	76,808 (17.2%)	1,288 (18.6%)	12,553 (18.7%)
2005–2009	7,217 (16.1%)	71,557 (16.1%)	1,295 (18.7%)	12,495 (18.6%)
2010–2015	12,640 (28.1%)	125,058 (28.1%)	2,399 (34.6%)	22,963 (34.2%)
Setting for first eczema diagnosis				
Inpatient	12,588 (28.0%)	N/A	1,172 (16.9%)	11,246 (16.7%)
Outpatient clinic	31,296 (69.6%)	N/A	5,559 (80.1%)	53,935 (80.3%)
Emergency room	1,061 (2.4%)	N/A	208 (3.0%)	1,998 (3.0%)
Lifestyle related diseases[a]				
Chronic obstructive pulmonary disease	555 (1.2%)	1,568 (0.4%)	272 (3.9%)	897 (1.3%)
Hyperlipidemia or treatment of hyperlipidemia	533 (1.2%)	4,389 (1.0%)	505 (7.3%)	4,249 (6.3%)
Hypertension or antihypertensive treatment	2,012 (4.5%)	16,601 (3.7%)	1,631 (23.5%)	13,568 (20.2%)
Alcohol-related conditions	591 (1.3%)	4,747 (1.1%)	392 (5.6%)	2,873 (4.3%)
Ischemic heart disease	274 (0.6%)	2,011 (0.5%)	268 (3.9%)	1,970 (2.9%)
Hospital-diagnosed obesity	474 (1.1%)	3,733 (0.8%)	227 (3.3%)	2,277 (3.4%)
Type II diabetes	248 (0.6%)	2,325 (0.5%)	221 (3.2%)	1,963 (2.9%)
Other comorbidities[a]				
Immunosuppression	821 (1.8%)	1,589 (0.4%)	526 (7.6%)	1,061 (1.6%)
Asthma	9,221 (20.5%)	12,588 (2.8%)	1,106 (15.9%)	1,518 (2.3%)
Prescriptions[a]				
Oral glucocorticoids	3,379 (7.5%)	6,220 (1.4%)	2,072 (29.9%)	4,390 (6.5%)
Systemic drugs for atopic eczema	452 (1.0%)	645 (0.1%)	317 (4.6%)	470 (0.7%)
Partnership status[a]				
Single	n/a	n/a	2,811 (40.5%)	24,810 (36.9%)
Married/cohabitating	n/a	n/a	3,929 (56.6%)	40,486 (60.3%)
Unknown	n/a	n/a	199 (2.9%)	1,883 (2.8%)
Education[a]				
Basic education (7–10 years)	n/a	n/a	1,598 (23.0%)	17,456 (26.0%)
Youth education, high school or similar (11–12 years)	n/a	n/a	2,870 (41.4%)	27,997 (41.7%)
Higher education (13+ years)	n/a	n/a	2,280 (32.9%)	19,921 (29.7%)
Unknown	n/a	n/a	191 (2.8%)	1,805 (2.7%)
Annual gross personal income[a,b]				
Low	n/a	n/a	< 665	6,695 (10.0%)
Intermediate	n/a	n/a	1,592 (22.9%)	14,204 (21.1%)
High	n/a	n/a	1,991 (28.7%)	19,953 (29.7%)
Very high	n/a	n/a	2,688 (38.7%)	26,280 (39.1%)
Unknown	n/a	n/a	< 5	47 (0.1%)

Individuals could contribute data as both eczema exposed and unexposed.

a. Based on records on or before cohort entry
b. Categorised based on quartiles of income, as low (<120,022 Danish kroner), intermediate (120,022–203,325 Danish kroner), high (203,325–286264 Danish kroner) or very high (286,264+ Danish kroner).

Abbreviations: SD: standard deviation; IQR: interquartile range.

© 2020 Mansfield KE et al. *JAMA Dermatology*.

eTable 4. England: Association (HR [99% CI]) between atopic eczema and cancer outcomes: comparing risk of cancer in those with atopic eczema to those without.

	Minimally adjusted				Adjusted for IMD and calendar period				Additionally adjusted for potential mediating variables (harmful alcohol use, smoking and BMI)			
Cancer outcome	Number	Person years at risk	Events	Hazard ratio (99% CI)*	Number	Person years at risk	Events	Hazard ratio (99% CI)*	Number	Person years at risk	Events	Hazard ratio (99% CI)*
CANCER OVERALL												
without atopic eczema	2,239,775	12,601,393	97,534	1 (ref)	2,239,775	12,601,393	97,534	1 (ref)	1,588,775	9,680,745	82,672	1 (ref)
with atopic eczema	471,970	2,864,446	24,402	**1.04 (1.02, 1.06)**	471,970	2,864,446	24,402	**1.04 (1.02, 1.06)**	392,644	2,538,790	22,690	**1.02 (0.99, 1.04)**
SPECIFIC CANCER OUTCOMES												
Lung												
without atopic eczema	2,239,775	12,601,393	9,715	1 (ref)	2,239,775	12,601,393	9,715	1 (ref)	1,588,775	9,680,745	7,992	1 (ref)
with atopic eczema	471,970	2,864,446	2,527	**1.08 (1.02, 1.16)**	471,970	2,864,446	2,527	**1.08 (1.01, 1.16)**	392,644	2,538,790	2,305	**1.02 (0.95, 1.10)**
Breast												
without atopic eczema	1,301,074	7,335,404	14,699	1 (ref)	1,301,074	7,335,404	14,699	1 (ref)	1,014,328	6,161,511	12,959	1 (ref)
with atopic eczema	276,510	1,711,089	3,468	**0.99 (0.93, 1.04)**	276,510	1,711,089	3,468	**0.99 (0.94, 1.04)**	244,834	1,597,522	3,267	**0.98 (0.92, 1.03)**
Prostate												
without atopic eczema	938,701	5,265,989	11,687	1 (ref)	938,701	5,265,989	11,687	1 (ref)	574,251	3,518,451	10,232	1 (ref)
with atopic eczema	195,460	1,153,357	2,993	**1.06 (1.00, 1.12)**	195,460	1,153,357	2,993	**1.06 (1.00, 1.13)**	147,805	941,256	2,842	**1.03 (0.97, 1.10)**
Pancreas												
without atopic eczema	2,239,775	12,601,393	2,280	1 (ref)	2,239,775	12,601,393	2,280	1 (ref)	1,588,775	9,680,745	1,872	1 (ref)
with atopic eczema	471,970	2,864,446	567	**0.99 (0.87, 1.14)**	471,970	2,864,446	567	**0.99 (0.86, 1.14)**	392,644	2,538,790	513	**0.96 (0.83, 1.11)**
Non-Hodgkin's lymphoma												
without atopic eczema	2,239,775	12,601,393	2,910	1 (ref)	2,239,775	12,601,393	2,910	1 (ref)	1,588,775	9,680,745	2,484	1 (ref)
with atopic eczema	471,970	2,864,446	825	**1.19 (1.07, 1.34)**	471,970	2,864,446	825	**1.20 (1.07, 1.34)**	392,644	2,538,790	772	**1.18 (1.04, 1.33)**
Hodgkin's lymphoma												
without atopic eczema	2,239,775	12,601,393	310	1 (ref)	2,239,775	12,601,393	310	1 (ref)	1,588,775	9,680,745	241	1 (ref)
with atopic eczema	471,970	2,864,446	103	**1.48 (1.07, 2.04)**	471,970	2,864,446	103	**1.48 (1.07, 2.04)**	392,644	2,538,790	93	**1.54 (1.08, 2.20)**
Leukaemia												
without atopic eczema	2,239,775	12,601,393	2,749	1 (ref)	2,239,775	12,601,393	2,749	1 (ref)	1,588,775	9,680,745	2,282	1 (ref)
with atopic eczema	471,970	2,864,446	693	**1.08 (0.96, 1.23)**	471,970	2,864,446	693	**1.09 (0.96, 1.23)**	392,644	2,538,790	644	**1.07 (0.94, 1.22)**
Multiple myeloma												
without atopic eczema	2,239,775	12,601,393	1,420	1 (ref)	2,239,775	12,601,393	1,420	1 (ref)	1,588,775	9,680,745	1,178	1 (ref)
with atopic eczema	471,970	2,864,446	382	**1.09 (0.93, 1.29)**	471,970	2,864,446	382	**1.10 (0.93, 1.30)**	392,644	2,538,790	353	**1.14 (0.96, 1.37)**
Central nervous system cancers												
without atopic eczema	2,239,775	12,601,393	1,372	1 (ref)	2,239,775	12,601,393	1,372	1 (ref)	1,588,775	9,680,745	1,143	1 (ref)
with atopic eczema	471,970	2,864,446	333	**1.00 (0.84, 1.19)**	471,970	2,864,446	333	**0.99 (0.83, 1.18)**	392,644	2,538,790	305	**0.97 (0.81, 1.17)**
Meningioma												
without atopic eczema	2,239,775	12,601,393	45	1 (ref)	2,239,775	12,601,393	45	1 (ref)	1,588,775	9,680,745	36	1 (ref)
with atopic eczema	471,970	2,864,446	7	**0.91 (0.30, 2.74)**	471,970	2,864,446	7	**0.99 (0.32, 3.04)**	392,644	2,538,790	6	**1.04 (0.28, 3.82)**

© 2020 Mansfield KE et al. *JAMA Dermatology*.

Brain neoplasm												
without atopic eczema	2,239,775	12,601,393	1,298	1 (ref)	2,239,775	12,601,393	1,298	1 (ref)	1,588,775	9,680,745	1,084	1 (ref)
with atopic eczema	471,970	2,864,446	312	0.97 (0.81, 1.16)	471,970	2,864,446	312	0.97 (0.81, 1.16)	392,644	2,538,790	286	0.95 (0.78, 1.15)
Spinal cord, cranial nerve or other CNS tumours												
without atopic eczema	2,239,775	12,601,393	35	1 (ref)	2,239,775	12,601,393	35	1 (ref)	1,588,775	9,680,745	27	1 (ref)
with atopic eczema	471,970	2,864,446	15	1.95 (0.77, 4.94)	471,970	2,864,446	15	2.06 (0.79, 5.34)	392,644	2,538,790	14	2.24 (0.77, 6.57)
Melanoma												
without atopic eczema	2,239,775	12,601,393	3,699	1 (ref)	2,239,775	12,601,393	3,699	1 (ref)	1,588,775	9,680,745	3,229	1 (ref)
with atopic eczema	471,970	2,864,446	831	0.96 (0.86, 1.07)	471,970	2,864,446	831	0.96 (0.86, 1.07)	392,644	2,538,790	787	0.96 (0.86, 1.08)
Non-melanoma skin cancer												
without atopic eczema	2,170,678	12,134,899	27,610	1 (ref)	2,170,678	12,134,899	27,610	1 (ref)	1,532,737	9,281,450	24,093	1 (ref)
with atopic eczema	462,364	2,783,295	7,555	1.10 (1.06, 1.14)	462,364	2,783,295	7,555	1.11 (1.06, 1.15)	383,797	2,461,693	7,151	1.08 (1.04, 1.13)

*Estimated hazard ratios from Cox regression with current age as the underlying timescale, stratified by matched set (matched on age at cohort entry, sex, general practice, and date at cohort entry). All models fitted to individuals with complete data for all variables included in each model and from valid matched sets, including one eczema exposed individual and at least one unexposed individual without eczema.

All models implicitly adjusted for sex, date at cohort entry and practice due to stratification by matched set, and age due to underlying timescale.

Abbreviations: BMI: Body mass index; IMD: Index of multiple deprivation.

© 2020 Mansfield KE et al. *JAMA Dermatology*.

eTable 5. **Denmark:** Association (HR [99% CI]) between atopic eczema and cancer outcomes: comparing risk of cancer in those with atopic eczema to those without.

	Number	Events	Person-years at risk	Minimally adjusted HR (99% CI)[a]	Additionally adjusted for mediating variables (lifestyle-related diseases) HR (99% CI)[b]
CANCER OVERALL					
Without atopic eczema	445,673	7,275	6,358,286	Ref	Ref
With atopic eczema	44,945	736	639,121	1.05 (0.95–1.16)	1.01 (0.92–1.12)
SPECIFIC CANCER OUTCOMES					
Lung					
Without atopic eczema	445,673	642	6,358,286	Ref	Ref
With atopic eczema	44,945	89	639,121	1.45 (1.08–1.96)	1.21 (0.88–1.65)
Breast					
Without atopic eczema	226,323	1,251	3,204,171	Ref	Ref
With atopic eczema	22,826	121	322,824	0.97 (0.76–1.24)	0.97 (0.76–1.24)
Prostate					
Without atopic eczema	219,350	508	3,154,115	Ref	Ref
With atopic eczema	22,119	44	316,297	1.06 (0.70–1.60)	1.07 (0.70–1.62)
Pancreas					
Without atopic eczema	445,673	138	6,358,286	Ref	Ref
With atopic eczema	44,945	19	639,121	1.62 (0.85–3.09)	1.46 (0.74–2.86)
Hodgkin's lymphoma					
Without atopic eczema	445,673	103	6,358,286	Ref	Ref
With atopic eczema	44,945	14	639,121	1.35 (0.65–2.82)	1.27 (0.59–2.72)
Non-Hodgkin's lymphoma					
Without atopic eczema	445,673	192	6,358,274	Ref	Ref
With atopic eczema	44,945	26	639,121	1.31 (0.76–2.26)	1.26 (0.73–2.18)
Leukaemia					
Without atopic eczema	445,673	303	6,358,286	Ref	Ref
With atopic eczema	44,945	31	639,121	1.04 (0.64–1.71)	1.02 (0.62–1.68)
Multiple myeloma					
Without atopic eczema	445,673	64	6,358,286	Ref	Ref
With atopic eczema	44,945	9	639,121	1.42 (0.55–3.62)	1.42 (0.55–3.69)
Central nervous system cancers[c]					
Without atopic eczema	445,673	216	6,358,286	Ref	Ref
With atopic eczema	44,945	17	639,121	0.78 (0.41–1.50)	0.79 (0.41–1.52)
Melanoma					
Without atopic eczema	443,683	813	6,344,854	Ref	Ref
With atopic eczema	44,819	52	638,353	0.64 (0.44–0.93)	0.64 (0.44–0.93)
Non-melanoma skin cancer					
Without atopic eczema	443,683	2,404	6,344,854	Ref	Ref
With atopic eczema	44,819	276	638,353	1.17 (0.99–1.38)	1.17 (0.99–1.38)

a. Estimated based on a Cox regression model with time since index date (date of atopic eczema diagnosis in eczema exposed, and index date of matched individual with atopic eczema in the cohort without eczema) as underlying timescale, stratified by matched set to account for matching factors (sex and birth year).
b. Mediation model: Adjusted additionally for time-varying lifestyle-related diseases.
c. Unable to stratify into specific types of CNS cancers due to sparse data for subtypes of CNS cancers

© 2020 Mansfield KE et al. *JAMA Dermatology*.

eTable 6. **England:** Hazard ratios (99% CIs)* for main and sensitivity analyses (after adjusting for calendar period and IMD).

Cancer type	Analysis**	Number of individuals	PYAR	Number of events	Fully adjusted HR (99% CI)*
Overall cancer	**Main analysis**	**2,629,640**	**15,465,839**	**121,936**	**1.04 (1.02, 1.06)**
	E1. Restricting to those with 3 years follow up	1,595,384	10,908,875	96,411	1.04 (1.02, 1.07)
	E2. Restricting to those with at least one consultation in the year before index	2,071,376	12,357,987	107,475	1.01 (0.99, 1.04)
	E3. Restricting to exposed individuals with newly-diagnosed eczema 12 months before cohort entry	1,461,186	8,020,251	71,191	1.04 (1.01, 1.07)
	E4. Redefined Cohort 1 (exposed unchanged, unexposed censored when eczema algorithm met, not 1st eczema diagnosis)	2,635,915	15,491,015	121,981	1.04 (1.02, 1.06)
	E5. Redefined Cohort 2 (eczema defined as diagnostic code only)	3,323,915	20,197,998	142,065	1.03 (1.01, 1.05)
	E6. Restricting to those joining the cohort from 2006 and additionally adjusting for ethnicity	1,095,186	3,761,200	28,619	1.03 (0.98, 1.07)
	E7. Restricting to those joining the cohort from 2004 onwards	1,716,025	6,758,199	44,593	1.06 (1.02, 1.09)
	E8. Additionally adjusting for diabetes mellitus	2,629,640	15,465,839	121,936	1.04 (1.02, 1.06)
	E9. Redefined cohort without allowing 12-months post eczema diagnosis before start of follow-up	2,782,784	16,748,221	131,455	1.05 (1.03, 1.07)
	E10. Additionally adjusting for immunosuppression	2,629,640	15,465,839	121,936	1.03 (1.01, 1.06)
	E11. Additionally adjusting for high-dose oral glucocorticoid	2,629,640	15,465,839	121,936	1.02 (1.00, 1.04)
	E12. Additionally adjusted for systemic therapies	2,629,640	15,465,839	121,936	1.04 (1.02, 1.06)
	E13. Overall cancer outcome excluding any skin cancers (melanoma and non-melanoma)	2,629,640	15,465,839	117,552	1.05 (1.02, 1.07)
	Main analysis additionally adjusting for potential mediators	***1,912,269***	***12,219,535***	***105,362***	***1.02 (0.99, 1.04)***
	E15. Restricting to those with complete smoking/BMI data in valid matched sets	1,912,269	12,219,535	105,362	1.03 (1.01, 1.05)
Lung	**Main analysis**	**2,629,640**	**15,465,839**	**12,242**	**1.08 (1.01, 1.16)**
	E1. Restricting to those with 3 years follow up	1,595,384	10,908,875	9,826	1.08 (1.01, 1.16)
	E2. Restricting to those with at least one consultation in the year before index	2,071,376	12,357,987	10,845	1.06 (0.99, 1.14)
	E3. Restricting to exposed individuals with newly-diagnosed eczema 12 months before cohort entry	1,461,186	8,020,251	7,017	1.09 (1.00, 1.19)
	E4. Redefined Cohort 1 (exposed unchanged, unexposed censored when eczema algorithm met, not 1st eczema diagnosis)	2,635,915	15,491,015	12,165	1.09 (1.02, 1.16)
	E5. Redefined Cohort 2 (eczema defined as diagnostic code only)	3,323,915	20,197,998	13,950	1.04 (0.97, 1.10)
	E6. Restricting to those joining the cohort from 2006 and additionally adjusting for ethnicity	1,095,186	3,761,200	2,712	1.06 (0.92, 1.22)
	E7. Restricting to those joining the cohort from 2004 onwards	1,716,025	6,758,199	4,259	1.09 (0.97, 1.21)
	E8. Additionally adjusting for diabetes mellitus	2,629,640	15,465,839	12,242	1.08 (1.01, 1.15)
	E9. Redefined cohort without allowing 12-months post eczema diagnosis before start of follow-up	2,782,784	16,748,221	13,129	1.11 (1.04, 1.18)
	E10. Additionally adjusting for immunosuppression	2,629,640	15,465,839	12,242	1.07 (1.01, 1.15)
	E11. Additionally adjusting for high-dose oral glucocorticoid	2,629,640	15,465,839	12,242	0.99 (0.93, 1.06)
	E12. Additionally adjusted for systemic therapies	2,629,640	15,465,839	12,242	1.08 (1.01, 1.15)
	E14. Censoring at specific cancer type only (rather than any cancer)	2,811,865	16,808,985	18,487	1.08 (1.03, 1.14)
	Main analysis additionally adjusting for potential mediators	***1,912,269***	***12,219,535***	***10,297***	***1.02 (0.95, 1.10)***
	E15. Restricting to those with complete smoking/BMI data in valid matched sets	1,912,269	12,219,535	10,297	1.08 (1.01, 1.16)
Breast	**Main analysis**	**1,523,468**	**9,046,492**	**18,167**	**0.99 (0.94, 1.04)**
	E1. Restricting to those with 3 years follow up	864,967	6,132,684	13,806	0.99 (0.94, 1.05)
	E2. Restricting to those with at least one consultation in the year before index	1,319,908	7,948,191	16,283	0.98 (0.92, 1.03)
	E3. Restricting to exposed individuals with newly-diagnosed eczema 12 months before cohort entry	864,731	4,747,499	10,548	0.98 (0.92, 1.05)
	E4. Redefined Cohort 1 (exposed unchanged, unexposed censored when eczema algorithm met, not 1st eczema diagnosis)	1,523,468	9,046,492	18,167	0.99 (0.94, 1.04)
	E5. Redefined Cohort 2 (eczema defined as diagnostic code only)	1,523,468	9,046,492	18,167	0.99 (0.94, 1.04)

© 2020 Mansfield KE et al. *JAMA Dermatology*.

	E6. Restricting to those joining the cohort from 2006 and additionally adjusting for ethnicity	663,976	2,278,722	4,215	0.92 (0.82, 1.03)
	E7. Restricting to those joining the cohort from 2004 onwards	976,484	3,822,666	6,658	0.98 (0.90, 1.08)
	E8. Additionally adjusting for diabetes mellitus	1,523,468	9,046,492	18,167	0.99 (0.94, 1.04)
	E9. Redefined cohort without allowing 12-months post eczema diagnosis before start of follow-up	1,523,468	9,046,492	18,167	0.99 (0.94, 1.04)
	E10. Additionally adjusting for immunosuppression	1,523,468	9,046,492	18,167	0.99 (0.94, 1.04)
	E11. Additionally adjusting for high-dose oral glucocorticoid	1,523,468	9,046,492	18,167	0.99 (0.94, 1.04)
	E12. Additionally adjusted for systemic therapies	1,523,468	9,046,492	18,167	0.99 (0.94, 1.04)
	E14. Censoring at specific cancer type only (rather than any cancer)	1,584,389	9,493,813	20,653	0.99 (0.94, 1.04)
	Main analysis additionally adjusting for potential mediators	*1,212,321*	*7,759,033*	*16,226*	*0.98 (0.92, 1.03)*
	E15. Restricting to those with complete smoking/BMI data in valid matched sets	1,212,321	7,759,033	16,226	0.98 (0.93, 1.03)
Pancreas	Main analysis	**2,629,640**	**15,465,839**	**2,847**	**0.99 (0.86, 1.14)**
	E1. Restricting to those with 3 years follow up	1,595,384	10,908,875	2,301	1.00 (0.86, 1.16)
	E2. Restricting to those with at least one consultation in the year before index	2,071,376	12,357,987	2,530	0.96 (0.83, 1.11)
	E3. Restricting to exposed individuals with newly-diagnosed eczema 12 months before cohort entry	1,461,186	8,020,251	1,689	0.97 (0.81, 1.16)
	E4. Redefined Cohort 1 (exposed unchanged, unexposed censored when eczema algorithm met, not 1st eczema diagnosis)	2,635,915	15,491,015	2,846	1.01 (0.88, 1.16)
	E5. Redefined Cohort 2 (eczema defined as diagnostic code only)	3,323,915	20,197,998	3,230	1.01 (0.88, 1.15)
	E6. Restricting to those joining the cohort from 2006 and additionally adjusting for ethnicity	1,095,186	3,761,200	659	0.86 (0.64, 1.16)
	E7. Restricting to those joining the cohort from 2004 onwards	1,716,025	6,758,199	999	0.94 (0.74, 1.19)
	E8. Additionally adjusting for diabetes mellitus	2,629,640	15,465,839	2,847	0.99 (0.86, 1.14)
	E9. Redefined cohort without allowing 12-months post eczema diagnosis before start of follow-up	2,782,784	16,748,221	3,009	1.04 (0.91, 1.19)
	E10. Additionally adjusting for immunosuppression	2,629,640	15,465,839	2,847	0.99 (0.86, 1.13)
	E11. Additionally adjusting for high-dose oral glucocorticoid	2,629,640	15,465,839	2,847	0.96 (0.84, 1.11)
	E12. Additionally adjusted for systemic therapies	2,629,640	15,465,839	2,847	0.99 (0.86, 1.14)
	E14. Censoring at specific cancer type only (rather than any cancer)	2,816,815	16,844,624	4,362	1.03 (0.92, 1.15)
	Main analysis additionally adjusting for potential mediators	*1,912,269*	*12,219,535*	*2,385*	*0.96 (0.83, 1.11)*
	E15. Restricting to those with complete smoking/BMI data in valid matched sets	1,912,269	12,219,535	2,385	0.97 (0.83, 1.12)
Prostate	Main analysis	**1,106,172**	**6,419,346**	**14,680**	**1.06 (1.00, 1.13)**
	E1. Restricting to those with 3 years follow up	730,129	4,774,766	11,924	1.07 (1.00, 1.14)
	E2. Restricting to those with at least one consultation in the year before index	751,396	4,409,534	12,779	1.00 (0.94, 1.07)
	E3. Restricting to exposed individuals with newly-diagnosed eczema 12 months before cohort entry	595,898	3,270,365	9,000	1.05 (0.97, 1.13)
	E4. Redefined Cohort 1 (exposed unchanged, unexposed censored when eczema algorithm met, not 1st eczema diagnosis)	1,106,172	6,419,346	14,680	1.06 (1.00, 1.13)
	E5. Redefined Cohort 2 (eczema defined as diagnostic code only)	1,106,172	6,419,346	14,680	1.06 (1.00, 1.13)
	E6. Restricting to those joining the cohort from 2006 and additionally adjusting for ethnicity	431,116	1,482,112	3,653	1.04 (0.92, 1.18)
	E7. Restricting to those joining the cohort from 2004 onwards	739,541	2,935,533	5,616	1.06 (0.96, 1.17)
	E8. Additionally adjusting for diabetes mellitus	1,106,172	6,419,346	14,680	1.06 (1.00, 1.13)
	E9. Redefined cohort without allowing 12-months post eczema diagnosis before start of follow-up	1,106,172	6,419,346	14,680	1.06 (1.00, 1.13)
	E10. Additionally adjusting for immunosuppression	1,106,172	6,419,346	14,680	1.06 (1.00, 1.13)
	E11. Additionally adjusting for high-dose oral glucocorticoid	1,106,172	6,419,346	14,680	1.06 (0.99, 1.12)
	E12. Additionally adjusted for systemic therapies	1,106,172	6,419,346	14,680	1.06 (1.00, 1.13)
	E14. Censoring at specific cancer type only (rather than any cancer)	1,161,532	6,808,456	18,358	1.04 (0.99, 1.10)
	Main analysis additionally adjusting for potential mediators	*699,754*	*4,459,707*	*13,074*	*1.03 (0.97, 1.10)*
	E15. Restricting to those with complete smoking/BMI data in valid matched sets	699,754	4,459,707	13,074	1.03 (0.97, 1.10)

© 2020 Mansfield KE et al. *JAMA Dermatology*.

Non-Hodgkin's lymphoma	**Main analysis**	**2,629,640**	**15,465,839**	**3,735**	**1.20 (1.07, 1.34)**
	E1. Restricting to those with 3 years follow up	1,595,384	10,908,875	2,950	1.18 (1.04, 1.34)
	E2. Restricting to those with at least one consultation in the year before index	2,071,376	12,357,987	3,280	1.18 (1.04, 1.32)
	E3. Restricting to exposed individuals with newly-diagnosed eczema 12 months before cohort entry	1,461,186	8,020,251	2,211	1.20 (1.03, 1.39)
	E4. Redefined Cohort 1 (exposed unchanged, unexposed censored when eczema algorithm met, not 1st eczema diagnosis)	2,635,915	15,491,015	3,744	1.19 (1.06, 1.33)
	E5. Redefined Cohort 2 (eczema defined as diagnostic code only)	3,323,915	20,197,998	4,371	1.21 (1.09, 1.35)
	E6. Restricting to those joining the cohort from 2006 and additionally adjusting for ethnicity	1,095,186	3,761,200	931	1.17 (0.93, 1.47)
	E7. Restricting to those joining the cohort from 2004 onwards	1,716,025	6,758,199	1,429	1.22 (1.01, 1.47)
	E8. Additionally adjusting for diabetes mellitus	2,629,640	15,465,839	3,735	1.19 (1.06, 1.34)
	E9. Redefined cohort without allowing 12-months post eczema diagnosis before start of follow-up	2,782,784	16,748,221	4,076	1.24 (1.11, 1.38)
	E10. Additionally adjusting for immunosuppression	2,629,640	15,465,839	3,735	1.17 (1.04, 1.31)
	E11. Additionally adjusting for high-dose oral glucocorticoid	2,629,640	15,465,839	3,735	1.17 (1.04, 1.31)
	E12. Additionally adjusted for systemic therapies	2,629,640	15,465,839	3,735	1.17 (1.05, 1.32)
	E14. Censoring at specific cancer type only (rather than any cancer)	2,808,882	16,788,912	5,572	1.19 (1.09, 1.31)
	Main analysis additionally adjusting for potential mediators	*1,912,269*	*12,219,535*	*3,256*	*1.18 (1.04, 1.33)*
	E15. Restricting to those with complete smoking/BMI data in valid matched sets	1,912,269	12,219,535	3,256	1.18 (1.05, 1.33)
Hodgkin's lymphoma	**Main analysis**	**2,629,640**	**15,465,839**	**413**	**1.48 (1.07, 2.04)**
	E1. Restricting to those with 3 years follow up	1,595,384	10,908,875	294	1.57 (1.09, 2.26)
	E2. Restricting to those with at least one consultation in the year before index	2,071,376	12,357,987	333	1.45 (1.02, 2.05)
	E3. Restricting to exposed individuals with newly-diagnosed eczema 12 months before cohort entry	1,461,186	8,020,251	217	1.28 (0.81, 2.03)
	E4. Redefined Cohort 1 (exposed unchanged, unexposed censored when eczema algorithm met, not 1st eczema diagnosis)	2,635,915	15,491,015	383	1.72 (1.24, 2.40)
	E5. Redefined Cohort 2 (eczema defined as diagnostic code only)	3,323,915	20,197,998	506	1.36 (1.00, 1.84)
	E6. Restricting to those joining the cohort from 2006 and additionally adjusting for ethnicity	1,095,186	3,761,200	114	1.45 (0.79, 2.67)
	E7. Restricting to those joining the cohort from 2004 onwards	1,716,025	6,758,199	190	1.68 (1.05, 2.68)
	E8. Additionally adjusting for diabetes mellitus	2,629,640	15,465,839	413	1.49 (1.08, 2.06)
	E9. Redefined cohort without allowing 12-months post eczema diagnosis before start of follow-up	2,782,784	16,748,221	442	1.77 (1.31, 2.40)
	E10. Additionally adjusting for immunosuppression	2,629,640	15,465,839	413	1.44 (1.03, 2.00)
	E11. Additionally adjusting for high-dose oral glucocorticoid	2,629,640	15,465,839	413	1.47 (1.06, 2.03)
	E12. Additionally adjusted for systemic therapies	2,629,640	15,465,839	413	1.49 (1.08, 2.06)
	E14. Censoring at specific cancer type only (rather than any cancer)	2,814,286	16,828,009	693	1.55 (1.21, 1.99)
	Main analysis additionally adjusting for potential mediators	*1,912,269*	*12,219,535*	*334*	*1.54 (1.08, 2.20)*
	E15. Restricting to those with complete smoking/BMI data in valid matched sets	1,912,269	12,219,535	334	1.52 (1.07, 2.16)
Multiple myeloma	**Main analysis**	**2,629,640**	**15,465,839**	**1,802**	**1.10 (0.93, 1.30)**
	E1. Restricting to those with 3 years follow up	1,595,384	10,908,875	1,427	1.11 (0.93, 1.34)
	E2. Restricting to those with at least one consultation in the year before index	2,071,376	12,357,987	1,590	1.07 (0.89, 1.27)
	E3. Restricting to exposed individuals with newly-diagnosed eczema 12 months before cohort entry	1,461,186	8,020,251	1,023	1.12 (0.89, 1.40)
	E4. Redefined Cohort 1 (exposed unchanged, unexposed censored when eczema algorithm met, not 1st eczema diagnosis)	2,635,915	15,491,015	1,740	1.18 (0.99, 1.39)
	E5. Redefined Cohort 2 (eczema defined as diagnostic code only)	3,323,915	20,197,998	2,029	1.18 (1.00, 1.38)
	E6. Restricting to those joining the cohort from 2006 and additionally adjusting for ethnicity	1,095,186	3,761,200	396	1.13 (0.78, 1.63)
	E7. Restricting to those joining the cohort from 2004 onwards	1,716,025	6,758,199	632	1.10 (0.82, 1.48)
	E8. Additionally adjusting for diabetes mellitus	2,629,640	15,465,839	1,802	1.10 (0.93, 1.30)
	E9. Redefined cohort without allowing 12-months post eczema diagnosis before start of follow-up	2,782,784	16,748,221	1,916	1.20 (1.02, 1.41)

© 2020 Mansfield KE et al. *JAMA Dermatology*.

	E10. Additionally adjusting for immunosuppression	2,629,640	15,465,839	1,802	1.09 (0.92, 1.29)
	E11. Additionally adjusting for high-dose oral glucocorticoid	2,629,640	15,465,839	1,802	1.08 (0.91, 1.28)
	E12. Additionally adjusted for systemic therapies	2,629,640	15,465,839	1,802	1.09 (0.92, 1.30)
	E14. Censoring at specific cancer type only (rather than any cancer)	2,815,380	16,834,441	2,484	1.09 (0.94, 1.26)
	Main analysis additionally adjusting for potential mediators	*1,912,269*	*12,219,535*	*1,531*	*1.14 (0.96, 1.37)*
	E15. Restricting to those with complete smoking/BMI data in valid matched sets	1,912,269	12,219,535	1,531	1.14 (0.96, 1.37)
Leukaemia	Main analysis	2,629,640	15,465,839	3,442	1.09 (0.96, 1.23)
	E1. Restricting to those with 3 years follow up	1,595,384	10,908,875	2,741	1.05 (0.92, 1.21)
	E2. Restricting to those with at least one consultation in the year before index	2,071,376	12,357,987	3,045	1.04 (0.91, 1.18)
	E3. Restricting to exposed individuals with newly-diagnosed eczema 12 months before cohort entry	1,461,186	8,020,251	1,921	1.10 (0.93, 1.30)
	E4. Redefined Cohort 1 (exposed unchanged, unexposed censored when eczema algorithm met, not 1st eczema diagnosis)	2,635,915	15,491,015	3,436	1.07 (0.95, 1.21)
	E5. Redefined Cohort 2 (eczema defined as diagnostic code only)	3,323,915	20,197,998	3,966	1.08 (0.97, 1.22)
	E6. Restricting to those joining the cohort from 2006 and additionally adjusting for ethnicity	1,095,186	3,761,200	794	1.16 (0.90, 1.49)
	E7. Restricting to those joining the cohort from 2004 onwards	1,716,025	6,758,199	1,260	1.26 (1.03, 1.54)
	E8. Additionally adjusting for diabetes mellitus	2,629,640	15,465,839	3,442	1.08 (0.96, 1.22)
	E9. Redefined cohort without allowing 12-months post eczema diagnosis before start of follow-up	2,782,784	16,748,221	3,714	1.06 (0.94, 1.20)
	E10. Additionally adjusting for immunosuppression	2,629,640	15,465,839	3,442	1.05 (0.93, 1.19)
	E11. Additionally adjusting for high-dose oral glucocorticoid	2,629,640	15,465,839	3,442	1.06 (0.94, 1.20)
	E12. Additionally adjusted for systemic therapies	2,629,640	15,465,839	3,442	1.08 (0.96, 1.22)
	E14. Censoring at specific cancer type only (rather than any cancer)	2,811,125	16,805,626	4,733	1.07 (0.97, 1.19)
	Main analysis additionally adjusting for potential mediators	*1,912,269*	*12,219,535*	*2,926*	*1.07 (0.94, 1.22)*
	E15. Restricting to those with complete smoking/BMI data in valid matched sets	1,912,269	12,219,535	2,926	1.07 (0.94, 1.22)
Central nervous system cancers	Main analysis	2,629,640	15,465,839	1,705	0.99 (0.83, 1.18)
	E1. Restricting to those with 3 years follow up	1,595,384	10,908,875	1,314	1.00 (0.82, 1.20)
	E2. Restricting to those with at least one consultation in the year before index	2,071,376	12,357,987	1,463	0.97 (0.81, 1.17)
	E3. Restricting to exposed individuals with newly-diagnosed eczema 12 months before cohort entry	1,461,186	8,020,251	993	0.82 (0.64, 1.04)
	E4. Redefined Cohort 1 (exposed unchanged, unexposed censored when eczema algorithm met, not 1st eczema diagnosis)	2,635,915	15,491,015	1,728	1.02 (0.85, 1.21)
	E5. Redefined Cohort 2 (eczema defined as diagnostic code only)	3,323,915	20,197,998	2,125	0.98 (0.84, 1.15)
	E6. Restricting to those joining the cohort from 2006 and additionally adjusting for ethnicity	1,095,186	3,761,200	424	0.83 (0.57, 1.21)
	E7. Restricting to those joining the cohort from 2004 onwards	1,716,025	6,758,199	697	0.78 (0.58, 1.04)
	E8. Additionally adjusting for diabetes mellitus	2,629,640	15,465,839	1,705	0.99 (0.83, 1.18)
	E9. Redefined cohort without allowing 12-months post eczema diagnosis before start of follow-up	2,782,784	16,748,221	1,874	1.02 (0.86, 1.20)
	E10. Additionally adjusting for immunosuppression	2,629,640	15,465,839	1,705	0.99 (0.83, 1.18)
	E11. Additionally adjusting for high-dose oral glucocorticoid	2,629,640	15,465,839	1,705	0.94 (0.78, 1.12)
	E12. Additionally adjusted for systemic therapies	2,629,640	15,465,839	1,705	0.99 (0.83, 1.17)
	E14. Censoring at specific cancer type only (rather than any cancer)	2,814,917	16,833,959	2,465	1.00 (0.86, 1.15)
	Main analysis additionally adjusting for potential mediators	*1,912,269*	*12,219,535*	*1,448*	*0.97 (0.81, 1.17)*
	E15. Restricting to those with complete smoking/BMI data in valid matched sets	1,912,269	12,219,535	1,448	0.97 (0.80, 1.17)
Meningioma	Main analysis	2,629,640	15,465,839	52	0.99 (0.32, 3.04)
	E1. Restricting to those with 3 years follow up	1,595,384	10,908,875	39	0.82 (0.21, 3.16)
	E2. Restricting to those with at least one consultation in the year before index	2,071,376	12,357,987	48	0.80 (0.24, 2.66)
	E3. Restricting to exposed individuals with newly-diagnosed eczema 12 months before cohort entry	1,461,186	8,020,251	34	1.49 (0.41, 5.34)

© 2020 Mansfield KE et al. *JAMA Dermatology*.

	E4. Redefined Cohort 1 (exposed unchanged, unexposed censored when eczema algorithm met, not 1st eczema diagnosis)	2,635,915	15,491,015	52	0.65 (0.21, 2.04)
	E5. Redefined Cohort 2 (eczema defined as diagnostic code only)	3,323,915	20,197,998	57	1.05 (0.36, 3.09)
	E6. Restricting to those joining the cohort from 2006 and additionally adjusting for ethnicity	1,095,186	3,761,200	17	0.13 (0.00, 5.76)
	E7. Restricting to those joining the cohort from 2004 onwards	1,716,025	6,758,199	21	0.70 (0.09, 5.38)
	E8. Additionally adjusting for diabetes mellitus	2,629,640	15,465,839	52	0.91 (0.28, 2.91)
	E9. Redefined cohort without allowing 12-months post eczema diagnosis before start of follow-up	2,782,784	16,748,221	52	0.79 (0.27, 2.35)
	E10. Additionally adjusting for immunosuppression	2,629,640	15,465,839	52	1.02 (0.33, 3.14)
	E11. Additionally adjusting for high-dose oral glucocorticoid	2,629,640	15,465,839	52	0.75 (0.22, 2.49)
	E12. Additionally adjusted for systemic therapies	2,629,640	15,465,839	52	0.99 (0.32, 3.05)
	E14. Censoring at specific cancer type only (rather than any cancer)	2,817,490	16,849,447	102	0.78 (0.34, 1.80)
	Main analysis additionally adjusting for potential mediators	***1,912,269***	***12,219,535***	***42***	***1.04 (0.28, 3.82)***
	E15. Restricting to those with complete smoking/BMI data in valid matched sets	1,912,269	12,219,535	42	0.88 (0.26, 2.96)
Brain neoplasm	**Main analysis**	**2,629,640**	**15,465,839**	**1,610**	**0.97 (0.81, 1.16)**
	E1. Restricting to those with 3 years follow up	1,595,384	10,908,875	1,247	0.97 (0.80, 1.18)
	E2. Restricting to those with at least one consultation in the year before index	2,071,376	12,357,987	1,376	0.95 (0.79, 1.15)
	E3. Restricting to exposed individuals with newly-diagnosed eczema 12 months before cohort entry	1,461,186	8,020,251	937	0.79 (0.62, 1.02)
	E4. Redefined Cohort 1 (exposed unchanged, unexposed censored when eczema algorithm met, not 1st eczema diagnosis)	2,635,915	15,491,015	1,634	1.00 (0.84, 1.20)
	E5. Redefined Cohort 2 (eczema defined as diagnostic code only)	3,323,915	20,197,998	2,007	0.96 (0.81, 1.13)
	E6. Restricting to those joining the cohort from 2006 and additionally adjusting for ethnicity	1,095,186	3,761,200	399	0.85 (0.58, 1.24)
	E7. Restricting to those joining the cohort from 2004 onwards	1,716,025	6,758,199	657	0.77 (0.57, 1.05)
	E8. Additionally adjusting for diabetes mellitus	2,629,640	15,465,839	1,610	0.97 (0.81, 1.16)
	E9. Redefined cohort without allowing 12-months post eczema diagnosis before start of follow-up	2,782,784	16,748,221	1,773	1.00 (0.84, 1.19)
	E10. Additionally adjusting for immunosuppression	2,629,640	15,465,839	1,610	0.96 (0.80, 1.15)
	E11. Additionally adjusting for high-dose oral glucocorticoid	2,629,640	15,465,839	1,610	0.92 (0.76, 1.10)
	E12. Additionally adjusted for systemic therapies	2,629,640	15,465,839	1,610	0.96 (0.80, 1.15)
	E14. Censoring at specific cancer type only (rather than any cancer)	2,815,079	16,834,933	2,286	0.97 (0.83, 1.13)
	Main analysis additionally adjusting for potential mediators	***1,912,269***	***12,219,535***	***1,370***	***0.95 (0.78, 1.15)***
	E15. Restricting to those with complete smoking/BMI data in valid matched sets	1,912,269	12,219,535	1,370	0.95 (0.78, 1.15)
Spinal cord, cranial nerve or other CNS tumours	**Main analysis**	**2,629,640**	**15,465,839**	**50**	**2.06 (0.79, 5.34)**
	E1. Restricting to those with 3 years follow up	1,595,384	10,908,875	33	2.29 (0.76, 6.87)
	E2. Restricting to those with at least one consultation in the year before index	2,071,376	12,357,987	46	1.94 (0.74, 5.12)
	E3. Restricting to exposed individuals with newly-diagnosed eczema 12 months before cohort entry	1,461,186	8,020,251	26	1.28 (0.27, 6.13)
	E4. Redefined Cohort 1 (exposed unchanged, unexposed censored when eczema algorithm met, not 1st eczema diagnosis)	2,635,915	15,491,015	50	1.83 (0.73, 4.59)
	E5. Redefined Cohort 2 (eczema defined as diagnostic code only)	3,323,915	20,197,998	67	1.66 (0.70, 3.96)
	E6. Restricting to those joining the cohort from 2006 and additionally adjusting for ethnicity	1,095,186	3,761,200	12	0.46 (0.01, 21.96)
	E7. Restricting to those joining the cohort from 2004 onwards	1,716,025	6,758,199	23	1.07 (0.18, 6.46)
	E8. Additionally adjusting for diabetes mellitus	2,629,640	15,465,839	50	2.05 (0.77, 5.44)
	E9. Redefined cohort without allowing 12-months post eczema diagnosis before start of follow-up	2,782,784	16,748,221	56	1.86 (0.70, 4.94)
	E10. Additionally adjusting for immunosuppression	2,629,640	15,465,839	50	2.09 (0.80, 5.42)
	E11. Additionally adjusting for high-dose oral glucocorticoid	2,629,640	15,465,839	50	2.12 (0.81, 5.56)
	E12. Additionally adjusted for systemic therapies	2,629,640	15,465,839	50	2.08 (0.80, 5.40)
	E14. Censoring at specific cancer type only (rather than any cancer)	2,817,417	16,849,091	163	1.49 (0.88, 2.50)

© 2020 Mansfield KE et al. *JAMA Dermatology*.

	Main analysis additionally adjusting for potential mediators	*1,912,269*	*12,219,535*	*41*	*2.24 (0.77, 6.57)*
	E15. Restricting to those with complete smoking/BMI data in valid matched sets	1,912,269	12,219,535	41	2.23 (0.79, 6.26)
Melanoma	**Main analysis**	**2,629,640**	**15,465,839**	**4,530**	**0.96 (0.86, 1.07)**
	E1. Restricting to those with 3 years follow up	1,595,384	10,908,875	3,491	0.95 (0.84, 1.07)
	E2. Restricting to those with at least one consultation in the year before index	2,071,376	12,357,987	3,933	0.94 (0.84, 1.06)
	E3. Restricting to exposed individuals with newly-diagnosed eczema 12 months before cohort entry	1,461,186	8,020,251	2,750	0.95 (0.83, 1.10)
	E4. Redefined Cohort 1 (exposed unchanged, unexposed censored when eczema algorithm met, not 1st eczema diagnosis)	2,635,915	15,491,015	4,499	0.97 (0.87, 1.08)
	E5. Redefined Cohort 2 (eczema defined as diagnostic code only)	3,323,915	20,197,998	5,557	0.95 (0.86, 1.05)
	E6. Restricting to those joining the cohort from 2006 and additionally adjusting for ethnicity	1,095,186	3,761,200	1,216	1.03 (0.84, 1.27)
	E7. Restricting to those joining the cohort from 2004 onwards	1,716,025	6,758,199	1,881	1.04 (0.88, 1.23)
	E8. Additionally adjusting for diabetes mellitus	2,629,640	15,465,839	4,530	0.96 (0.86, 1.07)
	E9. Redefined cohort without allowing 12-months post eczema diagnosis before start of follow-up	2,782,784	16,748,221	4,923	0.96 (0.86, 1.06)
	E10. Additionally adjusting for immunosuppression	2,629,640	15,465,839	4,530	0.96 (0.86, 1.07)
	E11. Additionally adjusting for high-dose oral glucocorticoid	2,629,640	15,465,839	4,530	0.96 (0.86, 1.08)
	E12. Additionally adjusted for systemic therapies	2,629,640	15,465,839	4,530	0.96 (0.86, 1.07)
	E14. Censoring at specific cancer type only (rather than any cancer)	2,804,283	16,751,303	5,731	0.97 (0.88, 1.07)
	Main analysis additionally adjusting for potential mediators	*1,912,269*	*12,219,535*	*4,016*	*0.96 (0.86, 1.08)*
	E15. Restricting to those with complete smoking/BMI data in valid matched sets	1,912,269	12,219,535	4,016	0.95 (0.85, 1.06)
Non-melanoma skin cancer	**Main analysis**	**2,555,437**	**14,918,194**	**35,165**	**1.11 (1.06, 1.15)**
	E1. Restricting to those with 3 years follow up	1,543,545	10,479,657	27,765	1.12 (1.08, 1.17)
	E2. Restricting to those with at least one consultation in the year before index	2,004,014	11,869,621	31,453	1.06 (1.02, 1.10)
	E3. Restricting to exposed individuals with newly-diagnosed eczema 12 months before cohort entry	1,406,672	7,681,817	21,238	1.12 (1.07, 1.18)
	E4. Redefined Cohort 1 (exposed unchanged, unexposed censored when eczema algorithm met, not 1st eczema diagnosis)	2,561,652	14,942,300	34,879	1.12 (1.08, 1.16)
	E5. Redefined Cohort 2 (eczema defined as diagnostic code only)	3,248,009	19,587,023	41,030	1.08 (1.04, 1.12)
	E6. Restricting to those joining the cohort from 2006 and additionally adjusting for ethnicity	1,059,533	3,619,292	8,489	1.09 (1.01, 1.18)
	E7. Restricting to those joining the cohort from 2004 onwards	1,667,990	6,533,767	13,517	1.12 (1.05, 1.19)
	E8. Additionally adjusting for diabetes mellitus	2,555,437	14,918,194	35,165	1.11 (1.06, 1.15)
	E9. Redefined cohort without allowing 12-months post eczema diagnosis before start of follow-up	2,708,157	16,168,137	38,249	1.14 (1.10, 1.18)
	E10. Additionally adjusting for immunosuppression	2,555,437	14,918,194	35,165	1.10 (1.06, 1.14)
	E11. Additionally adjusting for high-dose oral glucocorticoid	2,555,437	14,918,194	35,165	1.10 (1.06, 1.14)
	E12. Additionally adjusted for systemic therapies	2,555,437	14,918,194	35,165	1.10 (1.06, 1.14)
	E14. Censoring at specific cancer type only (rather than any cancer)	2,716,908	16,100,796	45,742	1.11 (1.08, 1.15)
	Main analysis additionally adjusting for potential mediators	*1,851,289*	*11,743,144*	*31,244*	*1.08 (1.04, 1.13)*
	E15. Restricting to those with complete smoking/BMI data in valid matched sets	1,851,289	11,743,144	31,244	1.08 (1.04, 1.12)

* Estimated hazard ratios from Cox regression with current age as underlying timescale, stratified by matched set (matched on age at cohort entry, sex, general practice, and date at cohort entry, with matched sets including one exposed individual and at least one unexposed individual).

** Note: Full methods and justification of each sensitivity analysis are included in Supplementary eTable 1 (All analysis numbers [e.g. E1, E2 etc.] reference the sensitivity analyses in eTable 1).

PYAR: person years at risk

© 2020 Mansfield KE et al. *JAMA Dermatology*.

eTable 7. **Denmark:** Hazard ratios (99% CIs) for main and sensitivity analyses.

		Atopic eczema cohort			HR (99% CI)	
Cancer type	Analysis*	Number of individuals	Number of events	Person-years at risk	Minimally adjusted [a]	Adjusted for potential mediators [b]
Cancer overall	Main	**44,945**	**736**	**639,121**	**1.05 (0.95–1.16)**	**1.01 (0.92–1.12)**
	D1. Restricting to those with 3 years follow up	40,059	591	509,728	1.01 (0.90–1.13)	0.99 (0.88–1.11)
	D2. Additionally adjusting for immunosuppression	44,945	736	639,121	0.78 (0.70–0.87)	0.76 (0.68–0.84)
	D3. Additionally adjusting for oral glucocorticoids	44,945	736	639,121	0.96 (0.87–1.07)	0.96 (0.86–1.06)
	D4. Additionally adjusted for systemic therapies for atopic eczema	44,945	736	639,121	0.98 (0.88–1.09)	0.95 (0.86–1.06)
	D5. Without censoring persons in the cohort without atopic eczema at diagnosis of atopic eczema	44,945	736	639,121	1.05 (0.95–1.16)	1.01 (0.92–1.12)
	D6. Analysis restricting to index date on or after 1 January 1996	34,038	428	354,386	1.04 (0.91–1.19)	1.01 (0.89–1.16)
	D7a. Analysis restricting to those aged 30 or more (prior to adjustment for SES)	6,939	507	73,786	1.12 (0.99–1.27)	n/a
	D7b. Analysis additionally adjusting for socioeconomic data in those aged 30 or more	6,939	507	73,786	1.12 (0.99–1.27)	1.07 (0.95–1.21)
	D8. Analysis after excluding any skin cancer diagnoses from the overall cancer outcome definition.	44,945	683	639,121	1.10 (0.99–1.22)	1.06 (0.95–1.18)
Lung	Main	**44,945**	**89**	**639,121**	**1.45 (1.08–1.96)**	**1.21 (0.88–1.65)**
	D1. Restricting to those with 3 years follow up	40,059	68	509,728	1.37 (0.97–1.92)	1.22 (0.86–1.74)
	D2. Additionally adjusting for immunosuppression	44,945	89	639,121	1.13 (0.81–1.56)	0.93 (0.66–1.30)
	D3. Additionally adjusting for oral glucocorticoids	44,945	89	639,121	1.03 (0.75–1.41)	0.98 (0.71–1.36)
	D4. Additionally adjusted for systemic therapies for atopic eczema	44,945	89	639,121	1.15 (0.83–1.60)	0.94 (0.67–1.33)
	D5. Without censoring persons in the cohort without atopic eczema at diagnosis of atopic eczema	44,945	89	639,121	1.45 (1.08–1.96)	1.21 (0.88–1.65)
	D6. Analysis restricting to index date on or after 1 January 1996	34,038	53	354,386	1.38 (0.94–2.02)	1.24 (0.83–1.85)
	D7a. Analysis restricting to those aged 30 or more (prior to adjustment for SES)	6,939	82	73,786	1.46 (1.07–1.99)	n/a
	D7b. Analysis additionally adjusting for socioeconomic data in those aged 30 or more	6,939	82	73,786	1.48 (1.08–2.03)	1.23 (0.88–1.71)
Breast	Main	**22,826**	**121**	**322,824**	**0.97 (0.76–1.24)**	**0.97 (0.76–1.24)**
	D1. Restricting to those with 3 years follow up	20,394	100	256,993	0.94 (0.71–1.23)	0.94 (0.71–1.23)
	D2. Additionally adjusting for immunosuppression	22,826	121	322,824	0.97 (0.76–1.25)	0.97 (0.76–1.25)
	D3. Additionally adjusting for oral glucocorticoids	22,826	121	322,824	0.98 (0.76–1.26)	0.98 (0.76–1.26)
	D4. Additionally adjusted for systemic therapies for atopic eczema	22,826	121	322,824	0.96 (0.74–1.23)	0.96 (0.74–1.23)
	D5. Without censoring persons in the cohort without atopic eczema at diagnosis of atopic eczema	22,826	121	322,824	0.97 (0.76–1.24)	0.97 (0.76–1.24)
	D6. Analysis restricting to index date on or after 1 January 1996	17,425	71	182,122	1.03 (0.74–1.42)	1.03 (0.74–1.42)
	D7a. Analysis restricting to those aged 30 or more (prior to adjustment for SES)	4,034	84	44,286	0.97 (0.72–1.31)	n/a
	D7b. Analysis additionally adjusting for socioeconomic data in those aged 30 or more	4,034	84	44,286	0.96 (0.71–1.29)	0.95 (0.71–1.29)
Prostate	Main	**22,119**	**44**	**316,297**	**1.06 (0.70–1.60)**	**1.07 (0.70–1.62)**
	D1. Restricting to those with 3 years follow up	19,665	33	252,735	1.01 (0.62–1.62)	1.03 (0.64–1.68)
	D2. Additionally adjusting for immunosuppression	22,119	44	316,297	1.02 (0.66–1.57)	1.03 (0.66–1.59)
	D3. Additionally adjusting for oral glucocorticoids	22,119	44	316,297	0.93 (0.60–1.45)	0.92 (0.59–1.44)
	D4. Additionally adjusted for systemic therapies for atopic eczema	22,119	44	316,297	0.96 (0.62–1.49)	0.97 (0.62–1.51)
	D5. Without censoring persons in the cohort without atopic eczema at diagnosis of atopic eczema	22,119	44	316,297	1.05 (0.70–1.60)	1.06 (0.70–1.61)
	D6. Analysis restricting to index date on or after 1 January 1996	16,613	30	172,264	0.94 (0.57–1.55)	0.95 (0.58–1.58)
	D7a. Analysis restricting to those aged 30 or more (prior to adjustment for SES)	2,905	42	29,500	1.02 (0.67–1.56)	n/a
	D7b. Analysis additionally adjusting for socioeconomic data in those aged 30 or more	2,905	42	29,500	1.02 (0.66–1.56)	1.02 (0.66–1.57)
Pancreas	Main	**44,945**	**19**	**639,121**	**1.62 (0.85–3.09)**	**1.46 (0.74–2.86)**

© 2020 Mansfield KE et al. *JAMA Dermatology*.

	D1. Restricting to those with 3 years follow up	40,059	13	509,728	1.24 (0.58–2.68)	1.13 (0.51–2.49)
	D2. Additionally adjusting for immunosuppression	44,945	19	639,121	1.60 (0.82–3.13)	1.50 (0.75–3.01)
	D3. Additionally adjusting for oral glucocorticoids	44,945	19	639,121	1.56 (0.79–3.05)	1.47 (0.73–2.97)
	D4. Additionally adjusted for systemic therapies for atopic eczema	44,945	19	639,121	1.58 (0.80–3.09)	1.45 (0.72–2.90)
	D5. Without censoring persons in the cohort without atopic eczema at diagnosis of atopic eczema	44,945	19	639,121	1.62 (0.85–3.10)	1.46 (0.75–2.86)
	D6. Analysis restricting to index date on or after 1 January 1996	34,038	9	354,386	1.35 (0.54–3.42)	1.28 (0.49–3.34)
	D7a. Analysis restricting to those aged 30 or more (prior to adjustment for SES)	6,939	18	73,786	1.74 (0.89–3.41)	n/a
	D7b. Analysis additionally adjusting for socioeconomic data in those aged 30 or more	6,939	18	73,786	1.75 (0.89–3.44)	1.59 (0.78–3.22)
Hodgkin's lymphoma	**Main**	**44,945**	**14**	**639,121**	**1.35 (0.65–2.82)**	**1.27 (0.59–2.72)**
	D1. Restricting to those with 3 years follow up	40,059	11	509,728	1.14 (0.50–2.60)	1.05 (0.45–2.47)
	D2. Additionally adjusting for immunosuppression	44,945	14	639,121	1.28 (0.60–2.72)	1.21 (0.56–2.64)
	D3. Additionally adjusting for oral glucocorticoids	44,945	14	639,121	1.20 (0.56–2.59)	1.16 (0.53–2.56)
	D4. Additionally adjusted for systemic therapies for atopic eczema	44,945	14	639,121	1.39 (0.66–2.93)	1.32 (0.61–2.85)
	D5. Without censoring persons in the cohort without atopic eczema at diagnosis of atopic eczema	44,945	14	639,121	1.36 (0.65–2.84)	1.28 (0.60–2.74)
	D6. Analysis restricting to index date on or after 1 January 1996	34,038	11	354,386	1.94 (0.83–4.55)	1.67 (0.68–4.11)
	D7a. Analysis restricting to those aged 30 or more (prior to adjustment for SES)	6,939	5	73,786	2.82 (0.73–10.92)	n/a
	D7b. Analysis additionally adjusting for socioeconomic data in those aged 30 or more	6,939	5	73,786	2.56 (0.53–12.38)	2.52 (0.43–14.91)
Non-Hodgkin's lymphoma	**Main**	**44,945**	**26**	**639,121**	**1.31 (0.76–2.26)**	**1.26 (0.73–2.18)**
	D1. Restricting to those with 3 years follow up	40,059	23	509,728	1.39 (0.78–2.47)	1.34 (0.74–2.40)
	D2. Additionally adjusting for immunosuppression	44,945	26	639,121	0.91 (0.50–1.66)	0.88 (0.48–1.61)
	D3. Additionally adjusting for oral glucocorticoids	44,945	26	639,121	0.96 (0.53–1.73)	0.94 (0.52–1.71)
	D4. Additionally adjusted for systemic therapies for atopic eczema	44,945	26	639,121	1.24 (0.71–2.17)	1.20 (0.68–2.11)
	D5. Without censoring persons in the cohort without atopic eczema at diagnosis of atopic eczema	44,945	26	639,121	1.32 (0.77–2.27)	1.26 (0.73–2.19)
	D6. Analysis restricting to index date on or after 1 January 1996	34,038	15	354,386	1.17 (0.58–2.38)	1.14 (0.55–2.34)
	D7a. Analysis restricting to those aged 30 or more (prior to adjustment for SES)	6,939	14	73,786	1.14 (0.55–2.39)	n/a
	D7b. Analysis additionally adjusting for socioeconomic data in those aged 30 or more	6,939	14	73,786	1.13 (0.54–2.38)	1.08 (0.51–2.28)
Leukaemia	**Main**	**44,945**	**31**	**639,121**	**1.04 (0.64–1.71)**	**1.02 (0.62–1.68)**
	D1. Restricting to those with 3 years follow up	40,059	27	509,728	1.26 (0.74–2.14)	1.25 (0.73–2.14)
	D2. Additionally adjusting for immunosuppression	44,945	31	639,121	0.87 (0.52–1.46)	0.84 (0.50–1.42)
	D3. Additionally adjusting for oral glucocorticoids	44,945	31	639,121	0.98 (0.59–1.63)	0.98 (0.59–1.63)
	D4. Additionally adjusted for systemic therapies for atopic eczema	44,945	31	639,121	0.98 (0.59–1.63)	0.97 (0.58–1.61)
	D5. Without censoring persons in the cohort without atopic eczema at diagnosis of atopic eczema	44,945	31	639,121	1.04 (0.64–1.70)	1.02 (0.62–1.68)
	D6. Analysis restricting to index date on or after 1 January 1996	34,038	21	354,386	1.03 (0.56–1.87)	0.99 (0.54–1.81)
	D7a. Analysis restricting to those aged 30 or more (prior to adjustment for SES)	6,939	10	73,786	0.85 (0.36–2.03)	n/a
	D7b. Analysis additionally adjusting for socioeconomic data in those aged 30 or more	6,939	10	73,786	0.83 (0.35–2.01)	0.80 (0.33–1.96)
Multiple myeloma	**Main**	**44,945**	**9**	**639,121**	**1.42 (0.55–3.62)**	**1.42 (0.55–3.69)**
	D1. Restricting to those with 3 years follow up	40,059	6	509,728	1.12 (0.36–3.48)	1.21 (0.38–3.89)
	D2. Additionally adjusting for immunosuppression	44,945	9	639,121	0.92 (0.31–2.69)	0.92 (0.31–2.72)
	D3. Additionally adjusting for oral glucocorticoids	44,945	9	639,121	0.97 (0.35–2.67)	1.03 (0.37–2.87)
	D4. Additionally adjusted for systemic therapies for atopic eczema	44,945	9	639,121	1.24 (0.43–3.54)	1.21 (0.41–3.54)
	D5. Without censoring persons in the cohort without atopic eczema at diagnosis of atopic eczema	44,945	9	639,121	1.42 (0.55–3.62)	1.42 (0.55–3.69)
	D6. Analysis restricting to index date on or after 1 January 1996	34,038	< 5		0.70 (0.15–3.30)	0.65 (0.13–3.18)

© 2020 Mansfield KE et al. *JAMA Dermatology*.

	D7a. Analysis restricting to those aged 30 or more (prior to adjustment for SES)	6,939	8	73,786	1.37 (0.51–3.70)	n/a
	D7b. Analysis additionally adjusting for socioeconomic data in those aged 30 or more	6,939	8	73,786	1.16 (0.40–3.36)	1.20 (0.41–3.56)
Central nervous system cancers	**Main**	**44,945**	**17**	**639,121**	**0.78 (0.41–1.50)**	**0.79 (0.41–1.52)**
	D1. Restricting to those with 3 years follow up	40,059	13	509,728	0.73 (0.35–1.53)	0.73 (0.35–1.55)
	D2. Additionally adjusting for immunosuppression	44,945	17	639,121	0.75 (0.39–1.46)	0.76 (0.39–1.49)
	D3. Additionally adjusting for oral glucocorticoids	44,945	17	639,121	0.66 (0.34–1.29)	0.67 (0.34–1.32)
	D4. Additionally adjusted for systemic therapies for atopic eczema	44,945	17	639,121	0.76 (0.39–1.47)	0.76 (0.39–1.49)
	D5. Without censoring persons in the cohort without atopic eczema at diagnosis of atopic eczema	44,945	17	639,121	0.78 (0.41–1.50)	0.79 (0.41–1.52)
	D6. Analysis restricting to index date on or after 1 January 1996	34,038	6	354,386	0.61 (0.21–1.80)	0.61 (0.20–1.84)
	D7a. Analysis restricting to those aged 30 or more (prior to adjustment for SES)	6,939	5	73,786	0.58 (0.18–1.90)	n/a
	D7b. Analysis additionally adjusting for socioeconomic data in those aged 30 or more	6,939	5	73,786	0.61 (0.18–2.01)	0.66 (0.19–2.22)
Melanoma	**Main**	**44,819**	**52**	**638,353**	**0.64 (0.44–0.93)**	**0.64 (0.44–0.93)**
	D1. Restricting to those with 3 years follow up	40,059	42	509,728	0.57 (0.38–0.85)	0.57 (0.38–0.85)
	D2. Additionally adjusting for immunosuppression	44,819	52	638,353	0.63 (0.44–0.92)	0.64 (0.44–0.93)
	D3. Additionally adjusting for oral glucocorticoids	44,819	52	638,353	0.68 (0.47–0.99)	0.68 (0.47–0.99)
	D4. Additionally adjusted for systemic therapies for atopic eczema	44,819	52	638,353	0.63 (0.44–0.92)	0.64 (0.44–0.93)
	D5. Without censoring persons in the cohort without atopic eczema at diagnosis of atopic eczema	44,945	53	639,121	0.64 (0.45–0.93)	0.65 (0.45–0.93)
	D6. Analysis restricting to index date on or after 1 January 1996	34,038	34	354,386	0.75 (0.48–1.19)	0.75 (0.48–1.20)
	D7a. Analysis restricting to those aged 30 or more (prior to adjustment for SES)	6,814	20	73,041	0.70 (0.39–1.28)	n/a
	D7b. Analysis additionally adjusting for socioeconomic data in those aged 30 or more	6,814	20	73,041	0.69 (0.38–1.26)	0.70 (0.38–1.28)
Non-melanoma skin cancer	**Main**	**44,819**	**276**	**638,353**	**1.17 (0.99–1.38)**	**1.17 (0.99–1.38)**
	D1. Restricting to those with 3 years follow up	40,059	239	509,728	1.18 (0.98–1.41)	1.19 (0.99–1.42)
	D2. Additionally adjusting for immunosuppression	44,819	276	638,353	1.05 (0.89–1.25)	1.06 (0.89–1.26)
	D3. Additionally adjusting for oral glucocorticoids	44,819	276	638,353	1.12 (0.94–1.33)	1.12 (0.94–1.33)
	D4. Additionally adjusted for systemic therapies for atopic eczema	44,819	276	638,353	1.07 (0.90–1.28)	1.08 (0.91–1.28)
	D5. Without censoring persons in the cohort without atopic eczema at diagnosis of atopic eczema	44,945	285	639,121	1.18 (1.01–1.40)	1.19 (1.01–1.40)
	D6. Analysis restricting to index date on or after 1 January 1996	34,038	162	354,386	1.07 (0.86–1.33)	1.07 (0.87–1.33)
	D7a. Analysis restricting to those aged 30 or more (prior to adjustment for SES)	6,814	212	73,041	1.29 (1.07–1.57)	n/a
	D7b. Analysis additionally adjusting for socioeconomic data in those aged 30 or more	6,814	212	73,041	1.29 (1.06–1.56)	1.29 (1.06–1.57)

Abbreviations: SES, socioeconomic status

a. Estimated based on a Cox regression model with time since index date (date of atopic eczema diagnosis in eczema exposed, and index date of matched individual with atopic eczema in the cohort without eczema) as underlying timescale, stratified by matched set to account for matching factors (sex and birth year) and adjusted for any additional variables in the sensitivity analysis. Reference group is cohort without atopic eczema.

b. Estimated based on a Cox regression model with time since index date (date of atopic eczema diagnosis in eczema exposed, and index date of matched individual with atopic eczema in the cohort without eczema) as underlying timescale, stratified by matched set to account for matching factors (sex and birth year) and adjusting for time-varying lifestyle-related diseases as well as any additional variables in the sensitivity analysis. Reference group is cohort without atopic eczema.

*Note: Full methods and justification of each sensitivity analysis are included in Supplementary eTable 1 (All analysis numbers [e.g. D1, D2 etc.] reference the sensitivity analyses in eTable 1).

© 2020 Mansfield KE et al. *JAMA Dermatology*.

eTable 8. England: Association (HR [99% CI]*) between severity of atopic eczema and cancer outcomes.

		Minimally adjusted				Adjusted for IMD and calendar period				Additionally adjusted for potential mediators (BMI, smoking and harmful alcohol use)			
		Number	Person years at risk	Events	Hazard ratio (99% CI)*	Number	Person years at risk	Events	Hazard ratio (99% CI)*	Number	Person years at risk	Events	Hazard ratio (99% CI)*
ANY CANCER													
	No atopic eczema	2,239,775	12,601,393	97,534	1 (reference)	2,239,775	12,601,393	97,534	1 (reference)	1,588,775	9,680,745	82,672	1 (reference)
	Mild eczema	319,226	1,714,072	12,491	1.01 (0.98, 1.03)	319,226	1,714,072	12,491	1.01 (0.98, 1.04)	260,597	1,490,487	11,597	0.99 (0.96, 1.02)
	Moderate eczema	167,142	983,533	10,218	1.07 (1.03, 1.10)	167,142	983,533	10,218	1.06 (1.03, 1.10)	146,972	897,747	9,525	1.03 (1.00, 1.07)
	Severe eczema	30,934	166,841	1,693	1.21 (1.12, 1.31)	30,934	166,841	1,693	1.21 (1.12, 1.31)	26,485	150,556	1,568	1.16 (1.07, 1.26)
SPECIFIC CANCER OUTCOMES													
Lung (C34)													
	No atopic eczema	2,239,775	12,601,393	9,715	1 (reference)	2,239,775	12,601,393	9,715	1 (reference)	1,588,775	9,680,745	7,992	1 (reference)
	Mild eczema	319,226	1,714,072	1,233	1.02 (0.93, 1.11)	319,226	1,714,072	1,233	1.02 (0.93, 1.12)	260,597	1,490,487	1,130	0.99 (0.89, 1.11)
	Moderate eczema	167,142	983,533	1,108	1.13 (1.02, 1.25)	167,142	983,533	1,108	1.12 (1.01, 1.23)	146,972	897,747	1,010	1.03 (0.92, 1.16)
	Severe eczema	30,934	166,841	186	1.37 (1.08, 1.75)	30,934	166,841	186	1.38 (1.08, 1.76)	26,485	150,556	165	1.22 (0.91, 1.63)
Breast (C50)													
	No atopic eczema	1,301,074	7,335,404	14,699	1 (reference)	1,301,074	7,335,404	14,699	1 (reference)	1,014,328	6,161,511	12,959	1 (reference)
	Mild eczema	190,670	1,044,193	1,939	0.94 (0.88, 1.01)	190,670	1,044,193	1,939	0.95 (0.88, 1.02)	167,751	969,670	1,815	0.93 (0.86, 1.00)
	Moderate eczema	95,409	568,239	1,310	1.04 (0.95, 1.13)	95,409	568,239	1,310	1.04 (0.95, 1.13)	86,765	535,235	1,245	1.04 (0.95, 1.14)
	Severe eczema	18,020	98,657	219	1.08 (0.87, 1.33)	18,020	98,657	219	1.07 (0.87, 1.33)	16,178	92,617	207	1.07 (0.86, 1.34)
Prostate (C61)													
	No atopic eczema	938,701	5,265,989	11,687	1 (reference)	938,701	5,265,989	11,687	1 (reference)	574,251	3,518,451	10,232	1 (reference)
	Mild eczema	128,556	669,879	1,554	1.13 (1.04, 1.23)	128,556	669,879	1,554	1.13 (1.04, 1.23)	92,843	520,811	1,480	1.09 (1.00, 1.19)
	Moderate eczema	71,733	415,295	1,269	0.99 (0.90, 1.09)	71,733	415,295	1,269	0.99 (0.91, 1.09)	60,205	362,506	1,206	0.98 (0.89, 1.07)
	Severe eczema	12,914	68,184	170	1.01 (0.79, 1.30)	12,914	68,184	170	1.01 (0.79, 1.30)	10,307	57,940	156	0.96 (0.74, 1.26)
Pancreas (C25)													
	No atopic eczema	2,239,775	12,601,393	2,280	1 (reference)	2,239,775	12,601,393	2,280	1 (reference)	1,588,775	9,680,745	1,872	1 (reference)
	Mild eczema	319,226	1,714,072	281	0.90 (0.74, 1.09)	319,226	1,714,072	281	0.90 (0.74, 1.09)	260,597	1,490,487	256	0.88 (0.71, 1.09)
	Moderate eczema	167,142	983,533	256	1.13 (0.92, 1.39)	167,142	983,533	256	1.13 (0.92, 1.39)	146,972	897,747	228	1.10 (0.88, 1.37)
	Severe eczema	30,934	166,841	30	0.88 (0.49, 1.59)	30,934	166,841	30	0.88 (0.49, 1.60)	26,485	150,556	29	0.77 (0.42, 1.43)
Hodgkin's lymphoma (C81)													
	No atopic eczema	2,239,775	12,601,393	310	1 (reference)	2,239,775	12,601,393	310	1 (reference)	1,588,775	9,680,745	241	1 (reference)
	Mild eczema	319,226	1,714,072	51	1.33 (0.86, 2.05)	319,226	1,714,072	51	1.31 (0.85, 2.04)	260,597	1,490,487	45	1.43 (0.88, 2.34)
	Moderate eczema	167,142	983,533	41	1.62 (0.95, 2.75)	167,142	983,533	41	1.63 (0.96, 2.78)	146,972	897,747	39	1.62 (0.92, 2.83)
	Severe eczema	30,934	166,841	11	2.07 (0.68, 6.27)	30,934	166,841	11	2.08 (0.69, 6.31)	26,485	150,556	9	2.05 (0.55, 7.59)
Non-Hodgkin's lymphoma (C82-C86)													
	No atopic eczema	2,239,775	12,601,393	2,910	1 (reference)	2,239,775	12,601,393	2,910	1 (reference)	1,588,775	9,680,745	2,484	1 (reference)
	Mild eczema	319,226	1,714,072	381	1.06 (0.90, 1.25)	319,226	1,714,072	381	1.06 (0.90, 1.25)	260,597	1,490,487	361	1.05 (0.89, 1.24)

© 2020 Mansfield KE et al. *JAMA Dermatology*.

	Moderate eczema	167,142	983,533	352	1.23 (1.04, 1.47)	167,142	983,533	352	1.24 (1.04, 1.48)	146,972	897,747	325	1.20 (1.00, 1.45)
	Severe eczema	30,934	166,841	92	2.08 (1.42, 3.04)	30,934	166,841	92	2.08 (1.42, 3.04)	26,485	150,556	86	2.14 (1.43, 3.20)
Leukaemia (C91-C95)													
	No atopic eczema	2,239,775	12,601,393	2,749	1 (reference)	2,239,775	12,601,393	2,749	1 (reference)	1,588,775	9,680,745	2,282	1 (reference)
	Mild eczema	319,226	1,714,072	345	1.03 (0.87, 1.22)	319,226	1,714,072	345	1.03 (0.87, 1.23)	260,597	1,490,487	321	1.01 (0.84, 1.22)
	Moderate eczema	167,142	983,533	298	1.12 (0.93, 1.36)	167,142	983,533	298	1.12 (0.93, 1.36)	146,972	897,747	276	1.10 (0.90, 1.34)
	Severe eczema	30,934	166,841	50	1.30 (0.80, 2.10)	30,934	166,841	50	1.30 (0.80, 2.10)	26,485	150,556	47	1.33 (0.80, 2.20)
Multiple myeloma (C90)													
	No atopic eczema	2,239,775	12,601,393	1,420	1 (reference)	2,239,775	12,601,393	1,420	1 (reference)	1,588,775	9,680,745	1,178	1 (reference)
	Mild eczema	319,226	1,714,072	193	0.99 (0.78, 1.25)	319,226	1,714,072	193	0.99 (0.78, 1.26)	260,597	1,490,487	176	1.05 (0.82, 1.35)
	Moderate eczema	167,142	983,533	168	1.23 (0.95, 1.60)	167,142	983,533	168	1.23 (0.95, 1.59)	146,972	897,747	156	1.24 (0.95, 1.64)
	Severe eczema	30,934	166,841	21	1.17 (0.59, 2.34)	30,934	166,841	21	1.21 (0.60, 2.41)	26,485	150,556	21	1.34 (0.66, 2.71)
Central nervous system cancers													
	No atopic eczema	2,239,775	12,601,393	1,372	1 (reference)	2,239,775	12,601,393	1,372	1 (reference)	1,588,775	9,680,745	1,143	1 (reference)
	Mild eczema	319,226	1,714,072	180	0.99 (0.78, 1.24)	319,226	1,714,072	180	0.99 (0.78, 1.24)	260,597	1,490,487	165	0.95 (0.74, 1.22)
	Moderate eczema	167,142	983,533	133	0.99 (0.74, 1.30)	167,142	983,533	133	0.97 (0.73, 1.28)	146,972	897,747	123	0.98 (0.73, 1.32)
	Severe eczema	30,934	166,841	20	1.19 (0.58, 2.42)	30,934	166,841	20	1.18 (0.58, 2.43)	26,485	150,556	17	1.12 (0.50, 2.52)
Melanoma (C43)													
	No atopic eczema	2,239,775	12,601,393	3,699	1 (reference)	2,239,775	12,601,393	3,699	1 (reference)	1,588,775	9,680,745	3,229	1 (reference)
	Mild eczema	319,226	1,714,072	457	0.96 (0.83, 1.11)	319,226	1,714,072	457	0.96 (0.83, 1.12)	260,597	1,490,487	428	0.96 (0.82, 1.12)
	Moderate eczema	167,142	983,533	334	0.98 (0.82, 1.16)	167,142	983,533	334	0.98 (0.83, 1.17)	146,972	897,747	320	0.99 (0.83, 1.18)
	Severe eczema	30,934	166,841	40	0.77 (0.47, 1.24)	30,934	166,841	40	0.78 (0.48, 1.25)	26,485	150,556	39	0.83 (0.50, 1.35)
Non-melanoma skin cancer (C44)													
	No atopic eczema	2,170,678	12,601,393	27,610	1 (reference)	2,170,678	12,601,393	27,610	1 (reference)	1,532,737	9,281,450	24,093	1 (reference)
	Mild eczema	313,258	1,670,216	3,973	1.11 (1.06, 1.17)	313,258	1,670,216	3,973	1.11 (1.06, 1.17)	255,088	1,448,809	3,780	1.10 (1.04, 1.16)
	Moderate eczema	162,784	951,670	3,038	1.06 (1.00, 1.12)	162,784	951,670	3,038	1.06 (1.00, 1.13)	142,913	867,434	2,869	1.04 (0.98, 1.11)
	Severe eczema	30,081	161,408	544	1.31 (1.13, 1.51)	30,081	161,408	544	1.31 (1.13, 1.51)	25,702	145,450	502	1.24 (1.06, 1.44)

*Estimated hazard ratios from Cox regression with current age as the underlying timescale, stratified by matched set (matched on age at cohort entry, sex, general practice, and date at cohort entry). All models fitted to individuals with complete data for all variables included in each model and from valid matched sets, including one eczema exposed individual and at least one unexposed individual without eczema.

All models implicitly adjusted for sex, date at cohort entry and practice due to stratification by matched set, and age due to underlying timescale.

© 2020 Mansfield KE et al. *JAMA Dermatology*.

eTable 9. England: Association (HR [99% CI]*) between atopic eczema disease activity and cancer outcomes.

	Fully adjusted (adjusted for IMD and calendar period)				Additionally adjusted for mediating variables (harmful alcohol use, smoking and BMI)			
Cancer outcome	Number	Person years at risk	Events	Hazard ratio (99% CI)*	Number	Person years at risk	Events	Hazard ratio (99% CI)*
ANY CANCER								
Without atopic eczema	2,239,775	12601393	97,534	1 (ref)	1,588,775	9,680,745	82,672	1 (ref)
Eczema – never active	121,509	767,005	4,403	1.03 (0.98, 1.08)	84,344	617,445	4,039	1.00 (0.95, 1.05)
Eczema – moderately active	245,906	1,504,217	12,613	1.01 (0.98, 1.04)	216,406	1,376,624	11,843	0.99 (0.96, 1.02)
Eczema – very active	104,555	593,224	7,386	1.11 (1.07, 1.15)	91,894	544,721	6,808	1.07 (1.03, 1.12)
SPECIFIC CANCER OUTCOMES								
Lung								
Without atopic eczema	2,239,775	12,601,393	9,715	1 (ref)	1,588,775	9,680,745	7,992	1 (ref)
Eczema – never active	121,509	767,005	430	1.02 (0.88, 1.19)	84,344	617,445	384	0.98 (0.82, 1.18)
Eczema – moderately active	245,906	1,504,217	1,215	0.99 (0.91, 1.09)	216,406	1,376,624	1,124	0.95 (0.85, 1.06)
Eczema – very active	104,555	593,224	882	1.28 (1.14, 1.44)	91,894	544,721	797	1.18 (1.03, 1.35)
Breast								
Without atopic eczema	1,301,074	7,335,404	14,699	1 (ref)	1,014,328	6,161,511	12,959	1 (ref)
Eczema – never active	65,955	433,345	715	0.97 (0.87, 1.09)	53,938	393,566	674	0.97 (0.86, 1.09)
Eczema – moderately active	148,971	922,492	1,885	0.97 (0.91, 1.05)	135,790	871,316	1,787	0.96 (0.89, 1.04)
Eczema – very active	61,584	355,251	868	1.03 (0.93, 1.15)	55,106	332,639	806	1.02 (0.91, 1.14)
Prostate								
Without atopic eczema	938,701	5,265,989	11,687	1 (ref)	574,251	3,518,451	10,232	1 (ref)
Eczema – never active	55,554	333,659	507	1.10 (0.95, 1.27)	30,405	223,877	481	1.06 (0.91, 1.23)
Eczema – moderately active	96,935	581,725	1,625	1.09 (1.00, 1.18)	80,612	505,297	1,552	1.06 (0.97, 1.15)
Eczema – very active	42,971	237,973	861	0.99 (0.88, 1.11)	36,788	212,082	809	0.97 (0.86, 1.09)
Pancreas								
Without atopic eczema	2,239,775	12,601,393	2,280	1 (ref)	1,588,775	9,680,745	1,872	1 (ref)
Eczema – never active	121,509	767,005	92	1.00 (0.72, 1.39)	84,344	617,445	78	0.87 (0.60, 1.26)
Eczema – moderately active	245,906	1,504,217	274	0.90 (0.74, 1.10)	216,406	1,376,624	250	0.90 (0.73, 1.11)
Eczema – very active	104,555	593,224	201	1.14 (0.90, 1.45)	91,894	544,721	185	1.12 (0.87, 1.45)
Non-Hodgkin's lymphoma								
Without atopic eczema	2,239,775	12,601,393	2,910	1 (ref)	1,588,775	9,680,745	2,484	1 (ref)
Eczema – never active	121,509	767,005	147	1.07 (0.83, 1.38)	84,344	617,445	138	1.07 (0.82, 1.41)
Eczema – moderately active	245,906	1,504,217	407	1.08 (0.92, 1.26)	216,406	1,376,624	387	1.06 (0.90, 1.25)
Eczema – very active	104,555	593,224	271	1.58 (1.28, 1.94)	91,894	544,721	247	1.53 (1.23, 1.91)
Hodgkin's lymphoma								
Without atopic eczema	2,239,775	12,601,393	310	1 (ref)	1,588,775	9,680,745	241	1 (ref)
Eczema – never active	121,509	767,005	16	0.96 (0.45, 2.02)	84,344	617,445	10	0.76 (0.30, 1.92)
Eczema – moderately active	245,906	1,504,217	59	1.59 (1.03, 2.44)	216,406	1,376,624	56	1.75 (1.10, 2.79)
Eczema – very active	104,555	593,224	28	1.83 (0.95, 3.52)	91,894	544,721	27	1.88 (0.93, 3.78)
Multiple myeloma								
Without atopic eczema	2,239,775	12,601,393	1,420	1 (ref)	1,588,775	9,680,745	1,178	1 (ref)
Eczema – never active	121,509	767,005	70	0.97 (0.66, 1.41)	84,344	617,445	65	1.03 (0.69, 1.54)
Eczema – moderately active	245,906	1,504,217	196	1.06 (0.84, 1.34)	216,406	1,376,624	183	1.10 (0.86, 1.41)
Eczema – very active	104,555	593,224	116	1.29 (0.94, 1.76)	91,894	544,721	105	1.32 (0.95, 1.85)
Leukaemia								
Without atopic eczema	2,239,775	12,601,393	2,749	1 (ref)	1,588,775	9,680,745	2,282	1 (ref)
Eczema – never active	121,509	767,005	134	1.07 (0.82, 1.40)	84,344	617,445	124	1.07 (0.80, 1.43)
Eczema – moderately active	245,906	1,504,217	348	1.06 (0.89, 1.26)	216,406	1,376,624	324	1.02 (0.84, 1.22)
Eczema – very active	104,555	593,224	211	1.15 (0.92, 1.44)	91,894	544,721	196	1.15 (0.91, 1.46)
Central nervous system cancers								
Without atopic eczema	2,239,775	12,601,393	1,372	1 (ref)	1,588,775	9,680,745	1,143	1 (ref)
Eczema – never active	121,509	767,005	79	1.30 (0.91, 1.86)	84,344	617,445	73	1.37 (0.93, 2.00)
Eczema – moderately active	245,906	1,504,217	172	0.92 (0.72, 1.17)	216,406	1,376,624	156	0.88 (0.68, 1.13)
Eczema – very active	104,555	593,224	82	0.90 (0.63, 1.28)	91,894	544,721	76	0.89 (0.61, 1.30)
Melanoma								
Without atopic eczema	2,239,775	12601393	3,699	1 (ref)	1,588,775	9,680,745	3,229	1 (ref)
Eczema – never active	121,509	767,005	158	0.90 (0.70, 1.16)	84,344	617,445	145	0.88 (0.68, 1.15)
Eczema – moderately active	245,906	1,504,217	460	0.94 (0.81, 1.09)	216,406	1,376,624	434	0.93 (0.80, 1.08)
Eczema – very active	104,555	593,224	213	1.05 (0.84, 1.30)	91,894	544,721	208	1.11 (0.88, 1.39)
Non-melanoma skin cancer								
Without atopic eczema	2,170,678	12134899	27,610	1 (ref)	1,532,737	9,281,450	24,093	1 (ref)
Eczema – never active	120,516	753,459	1,266	1.07 (0.98, 1.17)	83,466	604,623	1,195	1.06 (0.96, 1.16)
Eczema – moderately active	240,842	1,460,412	4,080	1.10 (1.05, 1.16)	211,641	1,334,628	3,894	1.08 (1.02, 1.14)
Eczema – very active	101,006	569,423	2,209	1.14 (1.06, 1.22)	88,690	522,442	2,062	1.11 (1.03, 1.20)

*Estimated hazard ratios from Cox regression with current age as the underlying timescale, stratified by matched set (matched on age at cohort entry, sex, general practice, and date at cohort entry). All models fitted to individuals with complete data for all variables included in each model and from valid matched sets, including one eczema exposed individual and at least one unexposed individual without eczema.

© 2020 Mansfield KE et al. *JAMA Dermatology*.

All models implicitly adjusted for sex, date at cohort entry and practice due to stratification by matched set, and age due to underlying timescale.

© 2020 Mansfield KE et al. *JAMA Dermatology*.

eTable 10. England: Adjusted hazard ratios (99% CIs) for the association between atopic eczema and cancer, stratified by sex (adjusted for calendar period and IMD).

Outcome	Number of individuals	PYAR	Number of events	Fully adjusted HR (99% CI)	Interaction p-value
Cancer overall					<0.01
Males	1,106,172	6,419,346	59,667	1.08 (1.05, 1.11)	
Females	1,523,468	9,046,492	62,269	1.01 (0.98, 1.04)	
Lung					0.22
Males	1,106,172	6,419,346	6,675	1.11 (1.02, 1.22)	
Females	1,523,468	9,046,492	5,567	1.05 (0.95, 1.15)	
Pancreas					0.88
Males	1,106,172	6,419,346	1,259	0.97 (0.79, 1.20)	
Females	1,523,468	9,046,492	1,588	1.00 (0.83, 1.20)	
Non-Hodgkin's lymphoma					0.39
Males	1,106,172	6,419,346	1,888	1.16 (0.98, 1.36)	
Females	1,523,468	9,046,492	1,847	1.25 (1.06, 1.46)	
Hodgkin's lymphoma					0.17
Males	1,106,172	6,419,346	200	1.72 (1.10, 2.70)	
Females	1,523,468	9,046,492	213	1.24 (0.77, 1.98)	
Leukaemia					0.10
Males	1,106,172	6,419,346	1,891	1.01 (0.85, 1.20)	
Females	1,523,468	9,046,492	1,551	1.18 (0.99, 1.41)	
Multiple myeloma					0.02
Males	1,106,172	6,419,346	909	1.30 (1.03, 1.64)	
Females	1,523,468	9,046,492	893	0.92 (0.72, 1.18)	
Central nervous system cancers					0.44
Males	1,106,172	6,419,346	822	0.94 (0.73, 1.21)	
Females	1,523,468	9,046,492	883	1.04 (0.82, 1.32)	
Melanoma					0.31
Males	1,106,172	6,419,346	1,887	1.00 (0.85, 1.19)	
Females	1,523,468	9,046,492	2,643	0.92 (0.80, 1.07)	
Non-melanoma skin cancer					0.57
Males	1,070,929	6,163,356	17,134	1.10 (1.04, 1.16)	
Females	1,484,508	8,754,838	18,031	1.11 (1.06, 1.17)	

*Estimated hazard ratios from Cox regression with current age as the underlying timescale, stratified by matched set (matched on age at cohort entry, sex, general practice, and date at cohort entry). All models fitted to individuals with complete data for all variables included in each model and from valid matched sets, including one eczema exposed individual and at least one unexposed individual without eczema.

All models implicitly adjusted for sex, date at cohort entry and practice due to stratification by matched set, and age due to underlying timescale.

P-values from likelihood ratio tests comparing models with and without an interaction term between atopic eczema and sex.

© 2020 Mansfield KE et al. *JAMA Dermatology*.

eTable 11. Denmark: Adjusted hazard ratios (99% CIs) for the association between atopic eczema and cancer, stratified by sex.

		Atopic eczema cohort			HR (99% CI)	
Cancer type	Sex	Number of individuals	Number of events	Person-years at risk	Minimally adjusted [a]	Adjusted for potential mediators [b]
Cancer overall	Female	22,826	413	322,824	0.98 (0.86–1.12)	0.95 (0.83–1.09)
	Male	22,119	323	316,297	1.15 (0.99–1.34)	1.10 (0.95–1.29)
Lung	Female	22,826	44	322,824	1.32 (0.86–2.00)	1.17 (0.75–1.81)
	Male	22,119	45	316,297	1.62 (1.06–2.48)	1.27 (0.81–2.00)
Pancreas	Female	22,826	10	322,824	1.43 (0.59–3.46)	1.45 (0.58–3.63)
	Male	22,119	9	316,297	1.89 (0.73–4.91)	1.56 (0.58–4.20)
Hodgkin's lymphoma	Female	22,826	< 5*	suppressed*	0.67 (0.18–2.52)	0.63 (0.16–2.47)
	Male	22,119	10	316,297	2.29 (0.92–5.68)	2.40 (0.90–6.35)
Non-Hodgkin's lymphoma	Female	22,826	8	322,824	0.80 (0.31–2.08)	0.83 (0.32–2.18)
	Male	22,119	18	316,297	1.83 (0.94–3.58)	1.68 (0.85–3.34)
Leukaemia	Female	22,826	14	322,824	1.24 (0.59–2.58)	1.21 (0.58–2.55)
	Male	22,119	17	316,297	0.92 (0.48–1.79)	0.92 (0.47–1.80)
Multiple myeloma	Female	22,826	5	322,824	1.97 (0.54–7.22)	2.09 (0.52–8.42)
	Male	22,119	< 5*	suppressed*	1.05 (0.27–4.15)	1.08 (0.27–4.36)
Central nervous system cancers	Female	22,826	10	322,824	0.88 (0.37–2.06)	0.91 (0.38–2.16)
	Male	22,119	7	316,297	0.68 (0.25–1.85)	0.71 (0.26–1.97)
Melanoma	Female	22,758	33	322,373	0.56 (0.35–0.89)	0.56 (0.35–0.89)
	Male	22,061	19	315,980	0.85 (0.46–1.58)	0.84 (0.45–1.56)
Non-melanoma skin cancer	Female	22,758	157	322,373	1.05 (0.84–1.30)	1.06 (0.85–1.32)
	Male	22,061	119	315,980	1.37 (1.06–1.77)	1.38 (1.06–1.79)

a. Estimated based on a Cox regression model with time since index date as underlying timescale, stratified by matched set to account for matching factors (sex, birth year).
b. Mediation model: Adjusted additionally for time-varying lifestyle-related diseases.

*To preserve patient confidentiality, where numbers of events are less than 5, we have not presented person years at risk.

© 2020 Mansfield KE et al. *JAMA Dermatology*.

eTable 12. England: Adjusted hazard ratios (99% CIs) for the association between atopic eczema stratified by current age (adjusted for calendar period and IMD).

Cancer type	Number of individuals	PYAR	Number of events	Fully adjusted HR (99% CI)	Interaction p-value
Cancer overall					0.87
18-44	1,524,499	6,791,178	6,635	1.06 (0.97, 1.15)	
45-64	925,479	4,941,016	31,917	1.05 (1.01, 1.09)	
65+	674,417	3,733,645	83,384	1.04 (1.01, 1.07)	
Lung					0.96
18-44	1,524,499	6,791,178	117	1.18 (0.61, 2.27)	
45-64	925,479	4,941,016	2,447	1.08 (0.93, 1.24)	
65+	674,417	3,733,645	9,678	1.08 (1.01, 1.17)	
Breast					0.94
18-44	913,290	4,045,058	1,818	0.97 (0.82, 1.15)	
45-64	524,176	2,857,985	8,090	0.99 (0.91, 1.07)	
65+	376,205	2,143,449	8,259	0.99 (0.91, 1.07)	
Prostate					0.06
18-44	611,209	2,746,120	17	2.67 (0.48, 14.88)	
45-64	401,303	2,083,031	2,644	1.18 (1.03, 1.34)	
65+	298,212	1,590,196	12,019	1.03 (0.97, 1.11)	
Pancreas					0.47
18-44	1,524,499	6,791,178	29	0.74 (0.16, 3.52)	
45-64	925,479	4,941,016	574	1.10 (0.82, 1.47)	
65+	674,417	3,733,645	2,244	0.96 (0.82, 1.13)	
Non-Hodgkin's lymphoma					0.03
18-44	1,524,499	6,791,178	273	1.43 (0.95, 2.17)	
45-64	925,479	4,941,016	1,050	1.38 (1.13, 1.69)	
65+	674,417	3,733,645	2,412	1.10 (0.95, 1.27)	
Hodgkin's lymphoma					0.53
18-44	1,524,499	6,791,178	179	1.27 (0.76, 2.12)	
45-64	925,479	4,941,016	113	1.49 (0.83, 2.70)	
65+	674,417	3,733,645	121	1.86 (0.98, 3.51)	
Leukaemia					0.10
18-44	1,524,499	6,791,178	201	1.57 (0.98, 2.52)	
45-64	925,479	4,941,016	769	1.11 (0.86, 1.42)	
65+	674,417	3,733,645	2,472	1.05 (0.90, 1.21)	
Multiple myeloma					0.39
18-44	1,524,499	6,791,178	24	0.96 (0.22, 4.08)	
45-64	925,479	4,941,016	411	1.30 (0.93, 1.82)	
65+	674,417	3,733,645	1,367	1.04 (0.85, 1.27)	
Central nervous system cancers					0.98
18-44	1,524,499	6,791,178	241	0.95 (0.58, 1.56)	
45-64	925,479	4,941,016	551	0.99 (0.74, 1.34)	
65+	674,417	3,733,645	913	0.99 (0.77, 1.25)	
Melanoma					0.60
18-44	1,524,499	6,791,178	777	1.05 (0.81, 1.36)	
45-64	925,479	4,941,016	1,524	0.92 (0.76, 1.11)	
65+	674,417	3,733,645	2,229	0.96 (0.82, 1.13)	
Non-melanoma skin cancer					0.19
18-44	1,521,283	6,775,546	1,696	1.11 (0.93, 1.31)	
45-64	903,715	4,816,337	9,366	1.06 (0.99, 1.14)	
65+	610,788	3,326,310	24,103	1.12 (1.07, 1.18)	

*Estimated hazard ratios from Cox regression with current age as the underlying timescale, stratified by matched set (matched on age at cohort entry, sex, general practice, and date at cohort entry). All models fitted to individuals with complete data for all variables included in each model and from valid matched sets, including one eczema exposed individual and at least one unexposed individual without eczema.

All models implicitly adjusted for sex, date at cohort entry and practice due to stratification by matched set, and age due to underlying timescale.

P-values from likelihood ratio tests comparing models with and without an interaction term between atopic eczema and age band.

© 2020 Mansfield KE et al. *JAMA Dermatology*.

eTable 13. Denmark: Adjusted hazard ratios (99% CIs) for the association between atopic eczema and cancer, stratified by age.

		Atopic eczema cohort			HR (99% CI)	
Cancer type	Age	Number of individuals	Number of events	Person-years at risk	Minimally adjusted [a]	Adjusted for potential mediators [b]
Cancer overall	< 18	31,772	125	473,641	0.96 (0.76–1.23)	0.95 (0.75–1.21)
	18-44	9,875	255	137,104	0.97 (0.81–1.14)	0.94 (0.79–1.12)
	45-64	2,418	240	23,326	1.12 (0.94–1.34)	1.07 (0.89–1.28)
	65+	880	116	5,051	1.21 (0.93–1.58)	1.15 (0.88–1.50)
Lung	< 18	31,772	< 5	*suppressed	0.67 (0.05–9.68)	0.84 (0.06–12.55)
	18-44	9,875	23	137,104	1.48 (0.83–2.65)	1.14 (0.60–2.14)
	45-64	2,418	42	23,326	1.32 (0.86–2.03)	1.07 (0.68–1.69)
	65+	880	23	5,051	1.84 (1.00–3.38)	1.57 (0.83–2.95)
Breast	< 18	14,427	9	213,744	1.04 (0.42–2.58)	1.08 (0.43–2.70)
	18-44	6,653	60	93,513	0.87 (0.61–1.23)	0.87 (0.61–1.23)
	45-64	1,298	39	12,834	1.05 (0.68–1.65)	1.06 (0.68–1.66)
	65+	448	13	2,733	1.25 (0.57–2.74)	1.25 (0.57–2.77)
Prostate	< 18	17,345	<5	*suppressed	–	–
	18-44	3,222	6	43,591	1.17 (0.38–3.61)	1.09 (0.35–3.43)
	45-64	1,120	20	10,504	0.92 (0.50–1.69)	0.91 (0.49–1.68)
	65+	432	18	2,318	1.19 (0.61–2.31)	1.23 (0.63–2.41)
Pancreas	< 18	31,772	0	–	–	–
	18-44	9,875	10	137,104	2.34 (0.93–5.87)	2.18 (0.83–5.71)
	45-64	2,418	6	23,326	1.07 (0.35–3.28)	1.02 (0.32–3.25)
	65+	880	< 5	*suppressed	1.92 (0.35–10.40)	1.88 (0.28–12.66)
Hodgkin's lymphoma	< 18	31,772	6	473,641	0.84 (0.28–2.50)	0.83 (0.27–2.54)
	18-44	9,875	6	137,104	2.36 (0.72–7.70)	2.15 (0.55–8.34)
	45-64	2,418	< 5	*suppressed	2.00 (0.11–35.95)	1.67 (0.08–36.37)
	65+	880	< 5	*suppressed	4.16 (0.18–97.60)	7.89 (0.12–507.08)
Non-Hodgkin's lymphoma	< 18	31,772	10	473,641	2.19 (0.89–5.39)	2.26 (0.91–5.62)
	18-44	9,875	8	137,104	1.08 (0.41–2.83)	0.88 (0.32–2.41)
	45-64	2,418	6	23,326	1.09 (0.36–3.36)	1.06 (0.34–3.33)
	65+	880	< 5	*suppressed	0.84 (0.12–5.81)	1.03 (0.13–7.89)
Leukaemia	< 18	31,772	17	473,641	1.09 (0.56–2.11)	1.11 (0.57–2.15)
	18-44	9,875	7	137,104	1.44 (0.50–4.11)	1.44 (0.49–4.19)
	45-64	2,418	< 5	*suppressed	0.34 (0.05–2.21)	0.30 (0.04–1.95)
	65+	880	5	5,051	1.49 (0.38–5.79)	1.53 (0.35–6.69)
Multiple myeloma	< 18	31,772	0	–	–	–
	18-44	9,875	< 5	*suppressed	1.04 (0.15–7.23)	0.85 (0.10–7.28)
	45-64	2,418	< 5	*suppressed	1.59 (0.39–6.50)	2.08 (0.48–9.05)
	65+	880	< 5	*suppressed	1.66 (0.31–8.89)	2.34 (0.33–16.60)
Central nervous system cancers	< 18	31,772	9	473,641	0.94 (0.38–2.32)	0.83 (0.33–2.10)
	18-44	9,875	< 5	*suppressed	0.40 (0.09–1.85)	0.44 (0.09–2.02)
	45-64	2,418	< 5	*suppressed	1.11 (0.28–4.39)	1.06 (0.25–4.50)
	65+	880	0	–	–	–
Melanoma	< 18	31,772	17	473,641	0.65 (0.34–1.25)	0.66 (0.34–1.26)
	18-44	9,858	26	137,006	0.60 (0.36–1.01)	0.61 (0.36–1.02)
	45-64	2,374	8	22,997	0.83 (0.32–2.15)	0.83 (0.32–2.18)
	65+	815	< 5	*suppressed	0.42 (0.03–5.91)	0.65 (0.04–10.22)
Non-melanoma skin cancer	< 18	31,772	15	473,641	0.64 (0.32–1.27)	0.63 (0.32–1.26)
	18-44	9,858	128	137,006	1.19 (0.94–1.52)	1.20 (0.94–1.53)
	45-64	2,374	92	22,997	1.26 (0.94–1.69)	1.29 (0.96–1.73)
	65+	815	41	4,709	1.21 (0.77–1.89)	1.19 (0.75–1.88)

a. Estimated based on a Cox regression model with time since index date as underlying timescale, stratified by matched set to account for matching factors.
b. Mediation model: Adjusted additionally for time-varying lifestyle-related diseases.

© 2020 Mansfield KE et al. *JAMA Dermatology*.

*To preserve patient confidentiality, where numbers of events are less than 5, we have not presented person years at risk.

© 2020 Mansfield KE et al. *JAMA Dermatology*.

eTable 14. England: Adjusted hazard ratios (99% CIs) for the association between atopic eczema stratified by asthma (adjusted for calendar period and IMD).

Cancer type	Number of individuals	PYAR	Number of events	Fully adjusted HR (99% CI)	Interaction p-value
Cancer overall					0.02
No asthma	2,245,438	12,989,140	102,928	1.04 (1.02, 1.07)	
Asthma	456,262	2,476,699	19,008	0.99 (0.91, 1.08)	
Lung					0.74
No asthma	2,245,438	12,989,140	9,682	1.04 (0.96, 1.13)	
Asthma	456,262	2,476,699	2,560	1.03 (0.81, 1.32)	
Breast					0.02
No asthma	1,300,365	7,555,986	15,369	1.00 (0.94, 1.07)	
Asthma	270,450	1,490,506	2,798	0.86 (0.69, 1.06)	
Prostate					0.72
No asthma	945,073	5,433,153	12,627	1.04 (0.97, 1.12)	
Asthma	185,812	986,193	2,053	1.15 (0.86, 1.55)	
Pancreas					0.57
No asthma	2,245,438	12,989,140	2,418	0.98 (0.83, 1.15)	
Asthma	456,262	2,476,699	429	1.04 (0.55, 1.97)	
Non-Hodgkin's lymphoma					0.53
No asthma	2,245,438	12,989,140	3,154	1.15 (1.00, 1.31)	
Asthma	456,262	2,476,699	581	1.31 (0.79, 2.18)	
Hodgkin's lymphoma					<0.01
No asthma	2,245,438	12,989,140	349	1.15 (0.77, 1.71)	
Asthma	456,262	2,476,699	64	3.65 (0.82, 16.25)	
Leukaemia					0.78
No asthma	2,245,438	12,989,140	2,956	1.10 (0.95, 1.26)	
Asthma	456,262	2,476,699	486	1.10 (0.61, 1.99)	
Central nervous system cancers					0.51
No asthma	2,245,438	12,989,140	1,435	1.01 (0.82, 1.24)	
Asthma	456,262	2,476,699	270	2.21 (1.01, 4.83)	
Melanoma					0.84
No asthma	2,245,438	12,989,140	3,904	0.97 (0.85, 1.10)	
Asthma	456,262	2,476,699	626	0.89 (0.55, 1.46)	
Non-melanoma skin cancer					0.04
No asthma	2,179,600	12,514,815	30,137	1.12 (1.07, 1.17)	
Asthma	444,802	2,403,379	5,028	1.06 (0.89, 1.26)	

*Estimated hazard ratios from Cox regression with current age as the underlying timescale, stratified by matched set (matched on age at cohort entry, sex, general practice, and date at cohort entry). All models fitted to individuals with complete data for all variables included in each model and from valid matched sets, including one eczema exposed individual and at least one unexposed individual without eczema.

All models implicitly adjusted for sex, date at cohort entry and practice due to stratification by matched set, and age due to underlying timescale.

P-values from likelihood ratio tests comparing models with and without an interaction term between atopic eczema and asthma.

NB: Models for multiple myeloma, and subtypes of CNS cancers were underpowered and are therefore omitted from this table.

© 2020 Mansfield KE et al. *JAMA Dermatology*.

eTable 15. **Denmark:** Adjusted hazard ratios (99% CIs) for the association between atopic eczema and cancer, stratified by asthma.

		Atopic eczema cohort			HR (99% CI)	
Cancer type	Asthma	Number of individuals	Number of events	Person-years at risk	Minimally adjusted [a]	Adjusted for potential mediators [b]
Cancer overall	No	35,724	596	503,067	1.03 (0.92–1.15)	1.00 (0.90–1.12)
	Yes	9,221	140	136,054	0.73 (0.29–1.84)	0.76 (0.27–2.10)
Lung	No	35,724	73	503,067	1.43 (1.03–1.99)	1.29 (0.91–1.81)
	Yes	9,221	16	136,054	–	–
Breast	No	18,776	100	263,142	0.96 (0.73–1.25)	0.95 (0.73–1.25)
	Yes	4,050	21	59,682	1.50 (0.14–15.75)	0.78 (0.01–54.77)
Prostate	No	16,948	30	239,925	0.86 (0.52–1.41)	0.85 (0.52–1.40)
	Yes	5,171	14	76,372	–	–
Pancreas	No	35,724	16	503,067	1.66 (0.82–3.38)	1.48 (0.71–3.10)
	Yes	9,221	< 5	*suppressed	–	–
Hodgkin's lymphoma	No	35,724	11	503,067	1.33 (0.58–3.06)	1.36 (0.58–3.17)
	Yes	9,221	< 5	*suppressed	–	–
Non-Hodgkin's lymphoma	No	35,724	19	503,067	1.13 (0.60–2.11)	1.09 (0.58–2.05)
	Yes	9,221	7	136,054	–	–
Leukaemia	No	35,724	28	503,067	1.17 (0.69–1.97)	1.15 (0.68–1.95)
	Yes	9,221	< 5	*suppressed	0.39 (0.02–7.74)	0.62 (0.03–14.99)
Multiple myeloma	No	35,724	7	503,067	1.48 (0.51–4.31)	1.54 (0.51–4.60)
	Yes	9,221	< 5	*suppressed	–	–
Central nervous system cancers	No	35,724	16	503,067	0.94 (0.48–1.85)	0.92 (0.46–1.81)
	Yes	9,221	< 5	*suppressed	–	–
Melanoma	No	35,619	37	502,420	0.54 (0.35–0.84)	0.54 (0.35–0.84)
	Yes	9,200	15	135,945	–	–
Non-melanoma skin cancer	No	35,619	232	502,420	1.14 (0.95–1.37)	1.15 (0.96–1.38)
	Yes	9,200	44	135,945	0.76 (0.07–8.12)	–

a. Estimated based on a Cox regression model with time since index date as underlying timescale, stratified by matched set to account for matching factors.
b. Mediation model: Adjusted additionally for time-varying lifestyle-related diseases.

*To preserve patient confidentiality where numbers of events are less than 5 we have not presented person years at risk.

© 2020 Mansfield KE et al. *JAMA Dermatology*.

eTable 16. Denmark: Codes used to define atopic eczema.

	ICD-8 code	ICD-10 code	Procedure codes in the Danish National Patient Registry	ATC code	Other codes	Notes
Atopic eczema overall	"691"	"DL20"				

Note: All subcodes of specified codes are included unless otherwise stated.

© 2020 Mansfield KE et al. *JAMA Dermatology*.

eTable 17. **Denmark:** Codes used to define outcomes (cancer overall and specific cancer outcomes).

	ICD-7	ICD-10
Cancer overall (excluding non-melanoma skin cancer)[a]	”140” ”141” ”142” ”143” ”144” ”145” ”146” ”147” ”148” ”149” ”150” ”151” ”152” ”153” ”154” ”155” ”156” ”157” ”158” ”159” ”160” ”161” ”162” ”163” ”164” ”165” ”166” ”167” ”168” ”169” ”170” ”171” ”172” ”173” ”174” ”175” ”176” ”177” ”178” ”179” ”180” ”181” ”182” ”183” ”184” ”185” ”186” ”187” ”188” ”189” ”190” ”192” ”193” ”194” ”195” ”196” ”197” ”198” ”199” ”200” ”201” ”202” ”203” ”27559” ”204” ”205” ”206” ”207”	”DC00” ”DC01” ”DC02” ”DC03” ”DC04” ”DC05” ”DC06” ”DC07” ”DC08” ”DC09” ”DC10” ”DC11” ”DC12” ”DC13” ”DC14” ”DC15” ”DC16” ”DC17” ”DC18” ”DC19” ”DC20” ”DC21” ”DC22” ”DC23” ”DC24” ”DC25” ”DC26” ”DC27” ”DC28” ”DC29” ”DC30” ”DC31” ”DC32” ”DC33” ”DC34” ”DC35” ”DC36” ”DC37” ”DC38” ”DC39” ”DC40” ”DC41” ”DC42” ”DC43” ”DC45” ”DC46” ”DC47” ”DC48” ”DC49” ”DC50” ”DC51” ”DC52” ”DC53” ”DC54” ”DC55” ”DC56” ”DC57” ”DC58” ”DC59” ”DC60” ”DC61” ”DC62” ”DC63” ”DC64” ”DC65” ”DC66” ”DC67” ”DC68” ”DC69” ”DC70” ”DC71” ”DC72” ”DC73 ”DC74” ”DC75” ”DC76” ”DC77” ”DC78” ”DC79” ”DC80” ”DC81” ”DC82” ”DC83” ”DC84” ”DC85” ”DC88” ”DC90” ”DC96” ”DC91” ”DC92” ”DC93” ”DC94” ”DC95” except codes for cutaneous lymphoma (”C826” ”C840” ”C841” ”C848” ”C863” ”C866” ”C884B”)
Lung		“DC34”
Breast		“DC50”
Prostate		“DC61”
Pancreatic		“DC25”
Skin		
Melanoma	”190”	“DC43”
Keratinocyte cancers	”191”	“DC44” if any of the following morphology codes: ”80903” ”80913” ”80923” ”80933” ”80973” ”81233” ”80513” ”80523” ”80703” ”80713” ”80723” ”80733” ”80743” ”80753” ”80763” ”80943” ”80953”
Haematological		All codes below
Lymphoma		“DC81” “DC82” “DC83” “DC84” “DC85” “DC86” “DC88” except codes for cutaneous lymphoma (”C826” ”C840” ”C841” ”C848” ”C863” ”C866” ”C884B”)
Multiple myeloma		“DC90”
Leukaemia		“DC91” “DC92” “DC93” “DC94” “DC95”
Central nervous system		All codes below
Meningioma		“DC70”
Cancer of the brain, hypophysis, corpus pineale and ductus craniopharyngealis		“DC71”
Spinal cord, cranial nerve or other central nervous system tumours		“DC72”

Note: We included all subcodes of specified codes unless otherwise stated. ICD-7 codes were used for excluding previous cancer and are thus listed for cancer overall and skin cancers.
[a]For exclusions of persons with previous cancer, we excluded all those with these codes except non-melanoma skin cancer. However, in analyses of keratinocyte skin cancer specifically, we excluded those with previous history of non-melanoma skin cancer (ICD-7: 191; ICD-10: C44) as well.
Furthermore, in order to explore the impact of potential ascertainment bias of skin cancers, we conducted a sensitivity analysis by repeating the all-cancer analysis after excluding all skin cancer (non-melanoma and melanoma skin cancer) from the outcome definition.

© 2020 Mansfield KE et al. *JAMA Dermatology*.

eTable 18. **Denmark:** Codes used to define covariables (confounders and mediators).

	ICD-8	ICD-10	Other codes in the Danish National Patient Registry	ATC	Categories/notes
Age					<18<br>18–44<br>45–64<br>≥65 years
Sex					Men<br>Women
Calendar period					1982–1999<br>2000–2004<br>2005–2009<br>2010–2015
Various lifestyle-related diseases (as proxy for unhealthy lifestyle)					
Chronic obstructive pulmonary disease	"491" "492"	"DJ41" "DJ42" "DJ43" "DJ44"			
Hyperlipidaemia or treatment of hyperlipidaemia	"27200"	"DE780"		"C10"	
Hypertension or antihypertensive treatment	"400" "401" "402" "403" "404"	"DI10" "DI11" "DI12" "DI13" "DI14" "DI15" "DI674"		"C02" "C03" "C07" "C08" "C09"	
Alcohol-related conditions	"291" "303" "57109" "57110" "57710" "979" "980"	"DF10" "DG312" "DG621" "DG721" "DI426" "DK292" "DK700" "DK703" "DK860" "DR780" "DT510" "DT519" "DZ721"		"N07BB01"	
Ischemic heart disease	"410" "411" "412" "413" "414"	"DI20" "DI21"<br>"DI22" "DI23" "DI24" "DI25"<br>"DT823D" "DT823E"	Procedure/surgery codes:<br>"30009" "30019" "30029" "30039" "30049" "30059" "30069" "30079" "30089" "30099" "30109" "30119" "30120" "30129" "30139" "30149" "30159" "30169" "30179" "30189" "30199" "KFNA" "KFNB" "KFNC" "KFND" "KFNE" "KFNH20" "KFNG" "KFNF"		
Hospital-diagnosed obesity	"277"	"DE65" "DE66"			
Type II diabetes	"250"	"DE11" "DO241"		"A10B" (excluding "A10BE01")	Any type II diabetes ICD code or second prescription for oral antidiabetic.
Socioeconomic status (education and social registries from Statistics Denmark)					For use in a sensitivity analysis among those aged 30 years or older at index date.
Highest attained educational level (primary education, secondary education, or higher education)					• Short term education (7-10 years) if afsp1e code = 10<br>• Medium term education (11-12 years) if afsp1e code 20<= & <40<br>• Long term education (13+ years) if afsp1e code 40<= & <90<br>• Missing if afsp1e has any other value
Partnership status (married/cohabitating vs. single)					Based on variables EFALLE (table BEF, during 1986-) and C_faelle_id (table FAIN, 1980-1985), which are generated by Statistics Denmark using an algorithm that identifies

© 2020 Mansfield KE et al. *JAMA Dermatology*.

					persons who are married or cohabitating.[a] Those who have non-missing data on these variables were recorded as in a partnership (=1) and remaining patients are considered single (=0)
Gross personal income					Categorized based on quartiles of variables PERINDKIALT_13 (during 1987-2016) and PERINDKIALT (1980-1986).
Immunosuppression (immunosuppressive disorders and drugs, excluding oral glucocorticoids)	”07983” ”28401” ”28402” ”28408” ”28409” ”75830” “203”<br>”204” ”205” ”206” ”207” ”200” ”201” ”202”	”DB20” ”DB21” ”DB22” ”DB23” ”DB24” ”DF024” ”DZ21” ”DT860” “DZ948C”<br><br>“DZ948” (if not “DZ948A” “DZ948B” or “DZ948C” and if coded as a B-diagnosis or additional diagnosis together with one of the following A-diagnoses “DC770” “DC81” “DC82” “DC83” “DC84” “DC85” “DC86” “DC87” “DC88” “DC89” “DC90” “DC91” “DC92” “DC93” “DC94” “DC95” “DC96”, “DD45” “DD46” “DD47” “DD5” “DD6” “DD7”<br>“DD80” “DD81” “DD82” “DD83” “DD84” “DD85” “DD87” “DD88” “DD89” “DT860” “DT860A” “DT888N”)<br><br>”DD611” ”DD612” ”DD613” ”DD618” ”DD619” ”DD81” ”DD820” ”DD821” ”DD821A” ”DD822” ”DD83” “DC90” ”DC91” ”DC92” ”DC93” ”DC94” ”DC95” ”DC81” ”DC82” ”DC83” ”DC84” ”DC85” ”DC86” ”DC88” ”DC96”	Procedure codes: ”BOQE” “BOQF” “BOHJ” “BWG” “BWHB”<br><br>Or<br><br>Any ATC code for “L01” “L04” “V02CA01” or “V02CA02” used as additional code in the Patient Registry	“L01” “L04” “V02CA01” “V02CA02”	When examining specific outcomes of myeloma, lymphoma and leukaemia the relevant codes (ICD-8: “203” ”204” ”205” ”206” ”207”<br>”200” ”201” ”202”; ICD-10: “DC90” ”DC91” ”DC92” ”DC93” ”DC94” ”DC95” ”DC81” ”DC82” ”DC83” ”DC84” ”DC85” ”DC86” ”DC88” ”DC96”) were excluded from the code list.
Use of oral glucocorticoids				”H02AB” together with DOSFORM “TAB” or “TABMOD” (identifying tablets)	
Systemic treatment for eczema (cyclosporine, azathioprine, mycophenolate, methotrexate, dupilumab)			Procedure codes: “BOHJ18B8” “BWHB83” “BWHA115” “BOHJ20” “BOHJ22”	“L04AX01” “L01BA01” “L04AX03” “L04AD01” “L04AA06” “D11AH05”	
Asthma	“493”	“DJ45” “DJ46”			

Note: All subcodes of specified codes were included unless otherwise stated; all types of contacts (inpatient, outpatient and emergency) and both primary and secondary diagnoses were considered. We used admission/prescription/record date for all variables. All variables were time-updated, except socioeconomic variables.
[a]Documentation available in Danish at:
https://www.dst.dk/da/Statistik/dokumentation/Times/forskningsservice/efalle
https://www.dst.dk/da/Statistik/dokumentation/Times/cpr-oplysninger/c-faelle-id

© 2020 Mansfield KE et al. *JAMA Dermatology*.
